# Metabolomics Based on UPLC-MS/MS Revealed the Metabolic Differences Among Four Species of Rhododendrons in Linzhi, Xizang

**DOI:** 10.3390/metabo16040226

**Published:** 2026-03-30

**Authors:** Ziqin Zhang, Sheng Kang, Mi Chen, Mudan Sang, Bingxin Lv, Yaao Pan, Zhenyu Chang

**Affiliations:** Key Laboratory of Veterinary Clinical Medicine of Xizang Autonomous Region, College of Animal Science, Xizang Agricultural and Animal Husbandry University, Linzhi 860000, China; m15025037546@163.com (Z.Z.); 18391656405@163.com (S.K.); cm2026cm@163.com (M.C.); 18143229571@163.com (M.S.); lvbingxin0729@163.com (B.L.); 17812054325@163.com (Y.P.)

**Keywords:** Metabolites, *Rhododendron triflorum*, *Rhododendron faucium*, *Rhododendron nivale*, *Rhododendron strigillosum*, UPLC-MS/MS

## Abstract

Although the genus Rhododendron is globally distributed and rich in bioactive constituents, the metabolomic landscapes of most species remain unexplored, hampering elucidation of their adaptive strategies and pharmaceutical potential. Objectives: This study sought to construct comprehensive metabolic atlases of four representative yet understudied Rhododendron species—*R. triflorum*, *R. faucium*, *R. nivale*, and *R. strigillosum*—and to quantify inter-specific metabolic divergence by UPLC-MS/MS-based, widely targeted metabolomics. Methods: The petals of four Rhododendron species were freeze-dried, pulverised, and extracted with 70% methanol (containing an internal standard). Metabolites were separated on an SB-C18 column (2.1 × 100 mm, 1.8 µm) using a 0–95% acetonitrile gradient (flow rate 0.35 mL min^−1^, 40 °C) and analysed by tandem mass spectrometry. Reliable quantification was ensured by molecular weight database matching, ion source standardisation, and quality control (QC), achieving a coefficient of variation (CV) < 15%. Principal component analysis (PCA) and optimised partial least squares discriminant analysis (OPLS-DA) were performed on standardised data with unit variance. Results: A total of 3705 metabolites were confidently identified, dominated by flavonoids (870), terpenoids (572), phenolic acids (394), and amino-acid derivatives (332). PCA and OPLS-DA models revealed clear species-specific clustering (R^2^Y ≥ 0.98, Q^2^ ≥ 0.95; permutation test *p* < 0.01). Comparative analysis yielded 1495 significantly differential metabolites; *R. triflorum* exhibited the highest cumulative abundance, followed by *R. faucium*, *R. nivale*, and *R. strigillosum.* KEGG enrichment highlighted “metabolic pathways” as the most significantly over-represented, together with flavonoid biosynthesis, phenylpropanoid metabolism, and terpenoid backbone biosynthesis. Conclusions: The study delivers the first high-coverage metabolomic reference for four neglected Rhododendron species, evidencing profound inter-specific metabolic differentiation centred on flavonoids, terpenoids, and phenolic acids. The data provide a robust foundation for understanding molecular adaptation to alpine environments and for accelerating targeted drug discovery from Rhododendron resources.

## 1. Introduction

Linzhi, situated in southeastern Xizang along the northern bank of the Yarlung Tsangpo River, experiences a dramatic elevation gradient influenced by the coupling of warm, moist air currents from the Indian Ocean with the Himalayan terrain. This creates an extreme vertical zone characterised by intense ultraviolet radiation, low oxygen levels, and alternating aridity and torrential rainfall [1]. Such conditions have fostered an exceptionally rich diversity of rhododendron species (Rhododendron). With approximately 180 species distributed across altitudes ranging from 1000 to 5800 m, it ranks among China’s most diverse regions for rhododendrons. This abundance holds significant value for maintaining the stability of Xizang’s fragile alpine ecosystems, while the variety of tree forms, floral colours, flower shapes, and blooming periods confer exceptional ornamental merit [2,3]. Despite the Linzhi region’s diverse rhododendron flora, current research primarily focuses on resource surveys and taxonomy. Studies on metabolic adaptation differentiation among species within shared habitats and the diversity of secondary metabolites remain limited. The metabolic adaptations of rhododendrons within the unique habitats of Xizang’s Linzhi region remain largely unexplored.

This study selected four species of rhododendron from Linzhi, Xizang, which flower at the same time but in different locations: *Rhododendron triflorum*, *Rhododendron faucium*, *Rhododendron nivale*, and *Rhododendron strigillosum*. *R*. *triflorum* (Hereinafter referred to as *R. triflorum, R. faucium, R. nivale, and R. strigillosum*) is an evergreen shrub of the Rhododendron genus within the Ericaceae family, reaching heights of 1–4 m. It bears 2–3 pale yellow flowers in terminal clusters, flowering from May to June, and is primarily distributed in the Hengduan Mountains of the Eastern Himalayas [4]. *R*. *faucium* is an evergreen shrub or small tree reaching 1.5–6.5 m in height. Its inflorescences bear 5–10 bell-shaped, pink corollas with conspicuous purple spots on the throat. Flowering occurs from April to June. It is distributed in the forest margins of southeastern Xizang at elevations of 2600–3800 m [5]. *R*. *nivale* is an evergreen shrub reaching 1–4 m in height. Its inflorescences are terminal, bearing 10–20 funnel-shaped, bell-like flowers that are white or pale pink with purplish-red throat spots. Flowering occurs from June to July. It is one of the signature species of the high-altitude Hengduan Mountains–Himalayan zone [6]. *R*. *strigillosum* is an evergreen shrub to small tree, 2–7 m tall, bearing terminal inflorescences of approximately 12 bell-shaped flowers. The corolla is red or white with purple spots at the throat, flowering from April to June. It is primarily distributed at elevations of 2050–3800 m in eastern Xizang and western Sichuan [7]. A review of the domestic and international literature reveals that these four rhododendron species currently represent gaps in the metabolomics literature [8,9]. Therefore, employing extensively targeted metabolomics based on Ultra-Performance Liquid Chromatography and tandem mass spectrometry (UPLC-MS/MS) to systematically elucidate the complex metabolic diversity of rhodo-dendrons could expand applications within this field.

UPLC-MS/MS high-throughput targeted metabolomics technology offers the advantages of high sensitivity, high resolution, and broad coverage [10]. This study aims to employ this technique for the systematic analysis of metabolite profiles in four Rhododendron species from the Linzhi region of Xizang. It seeks to identify inter-species differential metabolites and explore their metabolic pathways, thereby advancing our understanding of metabolic diversity within the Rhododendron genus and its adaptive mechanisms to environmental conditions. Furthermore, this research provides scientific foundations for cultivar selection and the development of medicinal plant resources.

## 2. Materials and Methods

### 2.1. Sample Collection

The *R. triflorum* was collected at 3155 m in Linzhi City, Xizhang Autonomous Region (29°39′50.209″ N, 94°18′20.448″ E), at approximately 10:00 am. The *R. faucium*, *R. nivale*, and *R. strigillosum* were collected at approximately 3680 m in Kasimu Village, Linzhi City, Xizhang Autonomous Region (29°32′28.152″ N, 94°29′35.419″ E), at around 11:00 am. At least fifteen specimens of each rhododendron species were collected, with all specimens comprising fresh, open flowers bearing stamens. Each rhododendron species was randomly divided into three replicates, designated as −1, −2, and −3, respectively.

### 2.2. Reagents and Instruments

Methanol (1.06007.4008: HPLC-MS grade, Merck), acetonitrile (5011403: HPLC-MS grade, Shanghai Xingke High-Purity Solvent Co., Ltd., Shanghai, China), formic acid (695076-100ML: HPLC-MS grade, Aladdin Industrial Corporation, Shanghai, China), and ultrapure water (18.2 MΩ·cm, 25 °C) were prepared using a Milli-Q water purification system (Millipore, Burlington, MA, USA).

The instruments used included a ball mill (MM 400, Retsch GmbH, Haan, Germany), Centrifuge (5424R, Eppendorf, Hamburg, Germany), constant-temperature metal homogeniser (MU-G02-0448, Hangzhou Mio Instruments Co., Ltd., Hangzhou, China), analytical balance (MS105DM, Mettler Toledo Instruments Limited, Zurich, Switzerland), centrifugal vacuum evaporator (CentriVap, LABCONCO, Kansas, MO, USA), vortex mixer (VORTEX-5, Kyllin-Bell, Haimen, China), ultrasonic cleaner (KQ5200E, Kunshan Ultrasonic Instrument Co., Ltd., Kunshan, China), pipette (Research plus, Eppendorf, Hamburg, Germany), automated liquid handler (Biomek i5, Beckman Coulter, Brea, CA, USA), and a sealing machine (Mini HES, Monad, Suzhou, China). Ultra-Performance Liquid Chromatography was employed (Sciex ExionLC™ AD, SCIEX, Framingham, MA, USA) (maximum working pressure: 1300 bar; Carryover: < 0.0015%) alongside high-resolution mass spectrometry (ZenoTOF™ 8600 System, SCIEX, Framingham, MA, USA), yielding a sensitivity improvement of 10× over previous generation and an acquisition rate up to 858 Hz.

### 2.3. Sample Extraction

Four samples of rhododendrons were placed in a freeze dryer (Scientz-100F) for vacuum freeze-drying for 63 h. They were then ground (30 Hz, 1.5 min) to powder form using a ball mill (MM 400) with zirconia milling balls 3 mm in diameter. A measure of 30 mg of the sample powder was weighed using an electronic balance (MS105DΜ), and 1500 μL of −20 °C pre-cooled 70% (*v*/*v*) methanolic aqueous internal standard solution was added to it (less than 30 mg added at the rate of 1500 μL extractant per 30 mg sample). The internal standard (IS) stock solution (1000 μg/mL) was prepared by dissolving 1 mg of IS in a 1 mL volumetric flask and diluting to volume with 70% (*v*/*v*) methanol. The IS working solution (250 μg/mL) was prepared by diluting the stock solution with 70% (*v*/*v*) methanol. The 1000 μg/mL stock solution was further diluted with 70% methanol to prepare a 250 μg/mL internal standard solution. The mixture was incubated at 4 °C with vortexing for 30 s every 30 min (6 times total). After centrifugation at 13,800× *g* for 3 min at 4 °C, the supernatant was filtered through a 0.22 μm nylon membrane and transferred to glass HPLC vials sealed with screw caps fitted with PTFE/silicone septa for UPLC-MS/MS analysis.

### 2.4. UPLC Conditions and ESI-Q TRAP-MS/MS

The sample extracts were analysed using a UPLC-ESI-MS/MS system (UPLC, ExionLC™ AD, https://sciex.com.cn/ (accessed on 3 September 2025)) and tandem mass spectrometry system (https://sciex.com.cn/). The analytical conditions were as follows. UPLC: column, Agilent SB-C18 (1.8 µm, 2.1 mm × 100 mm; Agilent Technologies, Inc., Santa Clara, CA, USA). The mobile phase consisted of solvent A, pure water with 0.1% (*v*/*v*) formic acid, and solvent B, acetonitrile with 0.1% (*v*/*v*) formic acid. Sample measurements were performed with a gradient programme that employed the starting conditions of 95% A and 5% B. Within 9 min, a linear gradient to 5% A and 95% B was programmed, and a composition of 5% A and 95% B was kept for 1 min. Subsequently, a composition of 95% A and 5.0% B was adjusted within 1.1 min and kept for 2.9 min. The flow velocity was set as 0.35 mL min^−1^. The column oven was set to 40 °C. The injection volume was 2 μL. The effluent was coupled to an ESI-triple quadrupole-linear ion trap (QTRAP) mass spectrometer for analysis.

The ESI source operation parameters were as follows: source temperature 500 °C; ion spray voltage (IS) 5500 V (positive ion mode)/−4500 V (negative ion mode); ion source gas I (GSI), gas II (GSII), curtain gas (CUR) were set at 50, 60, and 25 psi, respectively (all nitrogen, N_2_); the collision-activated dissociation(CAD) was high with 99.99% pure nitrogen. QQQ scans were acquired as MRM experiments with collision gas (nitrogen) set to medium. DP (declustering potential) and CE (collision energy) for individual MRM transitions were done with further DP and CE optimisation. A specific set of MRM transitions was monitored for each period according to the metabolites eluted within this period.

### 2.5. Qualitative and Quantitative Analysis of Metabolites

Based on the MWDB database (Version 2025, MetWare Biotechnology Co., Ltd., Wuhan, China) constructed from Wuhan Metware’s in-house metabolic database, qualitative analysis of compounds was performed using secondary spectral information (MS/MS spectra, including fragment ions and retention time). The mass accuracy tolerance for precursor ion matching was set at ±5 ppm. For metabolite identification, three confidence levels were applied according to spectral match scores and fragment numbers: Level 1 (score > 0.7, at least 3 matched fragment ions), Level 2 (score 0.5–0.7, at least 2 matched fragment ions), and Level 3 (consistent Q1, Q3, RT, DP, and CE with database entries). During analysis, isotopic signals; duplicate signals from K_+_, Na_+_, and NH_4+_ ions; and duplicate signals from fragment ions belonging to other larger molecular weight compounds were removed using Metware’s proprietary software. Fragment ions from higher molecular weight compounds were ascertained based on the annotation rules of the MWDB and the *m*/*z* correspondence between fragment and parent ions.

All four rhododendron samples underwent metabolite quantification analysis using the multiple reaction monitoring (MRM) mode on a triple quadrupole mass spectrometer. Initially, the quadrupole screened precursor ions (parent ions) of target compounds, excluding ions corresponding to other molecular weight substances, to preliminarily eliminate interference. Following collision-induced ionisation in the collision cell, precursor ions fragmented into numerous fragment ions. These fragments were then filtered through the triple quadrupole to select desired characteristic fragment ions, yielding distinct chromatographic peaks after subsequent processing. After obtaining metabolite mass spectrometry data for different samples, peak area integration was performed for all chromatographic peaks. Mass spectrometry peaks for the same metabolite across different samples underwent integration correction [11].

### 2.6. Data Quality Control and Statistical Analysis

The mass spectrometry data were processed using software Analyst 1.6.3 (AB SCIEX Pte. Ltd., Framingham, MA, USA). The sample extracts were mixed by combining an equal volume of each individual sample extract to prepare quality control (QC) samples for analysing the repeatability of the samples under the same processing method. During instrumental analysis, one QC sample was individually injected after every ten regular samples to monitor the repeatability of the analytical process. Pearson’s correlation coefficient r, as an evaluation index of biological repeat correlation, was calculated using the built-in cor function of R software(4.5.2). The Empirical Cumulative Distribution Function (ECDF) was used to analyse the frequency of CV (Coefficient of Variation) of substances less than the reference value (20%) to indicate the stability of the experimental data. After ensuring repeatability and stability, the metabolite content data were processed using unit variance scaling (UV) (unit variance scaling, i.e., autoscaling: mean-centred followed by division by the unit variance), and Principal component analysis (PCA) and cluster analysis were conducted using R software (www.r-project.org/). PCA plots and clustering heatmaps were generated from the processed data.

## 3. Results

### 3.1. Morphological Characteristics of Four Rhododendron Species

This study selected four azalea cultivars exhibiting significant phenotypic differences—*R. triflorum*, *R. faucium*, *R.nivale*, and *R. strigillosum* (in subsequent charts, they shall be abbreviated as Rtri, Rfau, Rniv, and Rstr, respectively)—to perform differential metabolite analysis.

**Figure 1:**
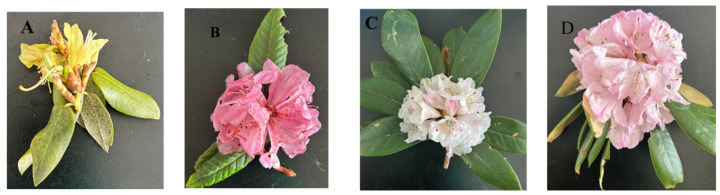
Morphology of four types of rhododendron: (**A**) *R. triflorum*; (**B**) *R. faucium*; (**C**) *R. nivale*; (**D**) *R. strigillosum.*

The flowers of *R. triflorum* are campanulate-funnelform with slightly undulate margins, predominantly pale yellow to lemon yellow, presenting a fresh and bright appearance. The stamens extend beyond the corolla, featuring slender filaments and dark brown to purplish-black anthers that form a striking contrast with the flower colour, rendering high distinctiveness. Typically, three flowers cluster at the branch apices, hence the specific epithet “*triflorum*” (Figure 1A). The flowers of *R. faucium* are broadly funnelform, with 5 rounded and spreading corolla lobes, presenting a full and expanded overall shape and a diameter of approximately 5–7 cm. The corolla is pale pink to rose pink externally, and the throat (base of the corolla tube) bears purple spots—hence the common name “throat-spotted rhododendron”. The stamens are of unequal lengths, with pinkish-white filaments and dark brown anthers; the pistil stigma slightly protrudes beyond the corolla, with a green apex that forms a cold-warm colour contrast with the throat spots (Figure 1B). The flowers of *R. nivale* are campanulate-funnelform with slightly undulate margins, showing radial symmetry overall. The flower stalks are 3–3.5 cm long, and the corolla is white or pale pink; some petals have purple-red spots or radial veins at the base. The filament bases are pubescent, the anthers are yellowish-brown, and the pistil slightly extends beyond the corolla with a capitate stigma. The corymbose terminal inflorescences usually bear 3–6 densely clustered flowers (Figure 1C). The flowers of *R. strigillosum* are long, funnelform, slightly tubulose-campanulate, with obtuse lobes and slightly undulate-curled margins. The flower diameter is approximately 4–5 cm, belonging to the medium to large-sized type. The corolla is pink to rose red overall, with purple spots on the throat. The filaments are slender with minute hairs at the base, the anthers are dark brown, and the pistil is slightly longer than the stamens with a green capitate stigma. The terminal racemose-umbellate inflorescences contain 12 flowers (Figure 1D).

### 3.2. Metabolomic Data Quality Assessment

**Figure 2:**
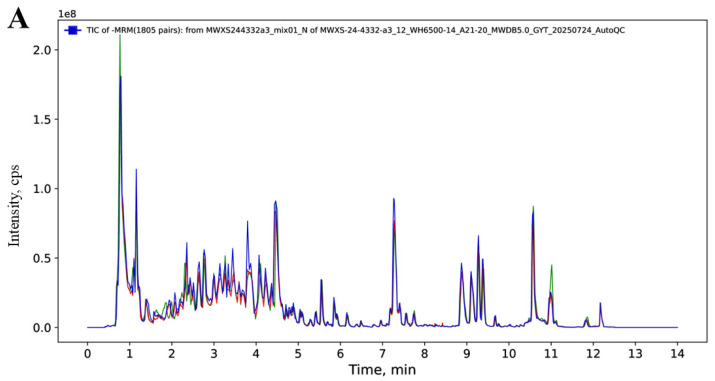

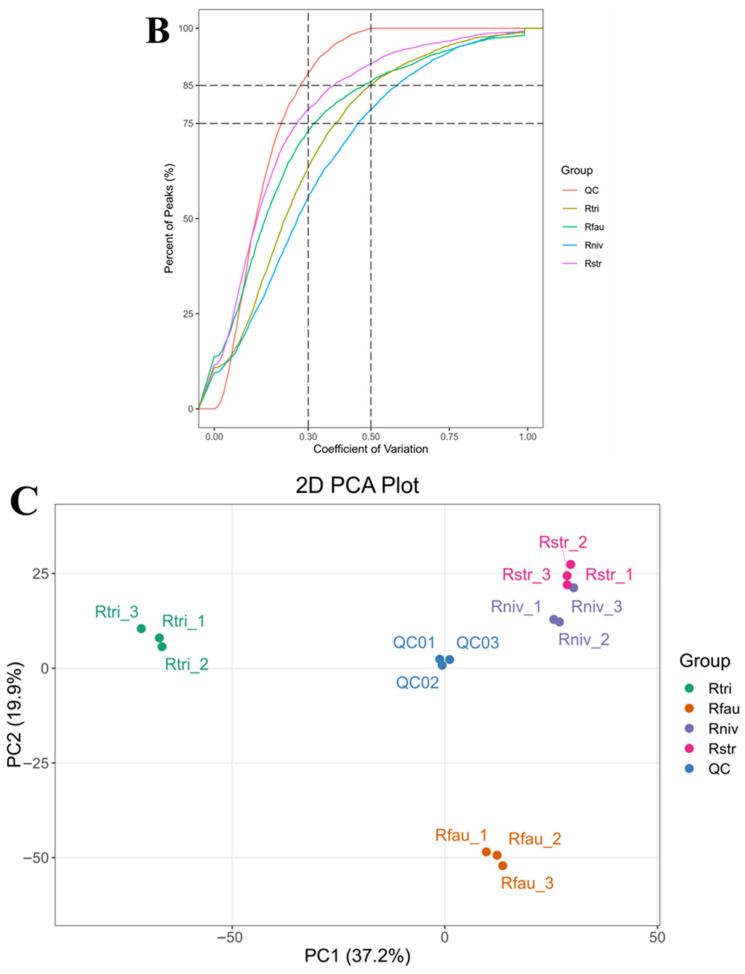
Data quality assessment. (**A**) Representative overlay of total ion chromatograms (TICs) from QC samples acquired in positive ion mode (3 injections: the first, middle, and last QC runs of the analytical sequence), demonstrating high instrumental stability throughout the sequence. (**B**) CV distribution plots for each sample group, where QC samples exhibit a high proportion of CV values, with stable experimental data. (**C**) Principal component analysis scatterplot of mass spectrometry data for samples and quality control samples indicates low variation within groups and differences between groups.

To ensure that all data is stable and reliable, strict quality control is carried out, and the total ion current (TIC) plots of the mixed QC samples show high signal intensity, stable baseline, and good peak shapes throughout the whole analysis process (Figure 2A). The high degree of overlap among QC TICs indicated excellent instrumental stability throughout the data acquisition process.

The reproducibility of metabolite detection was quantitatively assessed by calculating the Coefficient of Variation (CV) for all metabolites in the QC samples. A higher percentage of QC samples with lower CV values represents more stable experimental data. As shown in Figure 2B, the percentage of substances with CVs less than 0.5 for QC samples is higher than 75%, indicating that the experimental data are stable and reproducible.

Samples (including QC samples) were analysed by PCA to understand the overall metabolite differences between groups of samples and the magnitude of variability between samples within a group. The PCA results showed a trend in metabolome separation between groups, suggesting that metabolomes differed within the group of samples [12]. As shown in Figure 2C, the QC samples were far away from the other sample groups and tightly clustered in the PCA score plot, which reflected a stable analytical system and supported the reliability of subsequent differential statistics.

### 3.3. Qualitative and Quantitative Verification of Metabolites 

**Figure 3:**
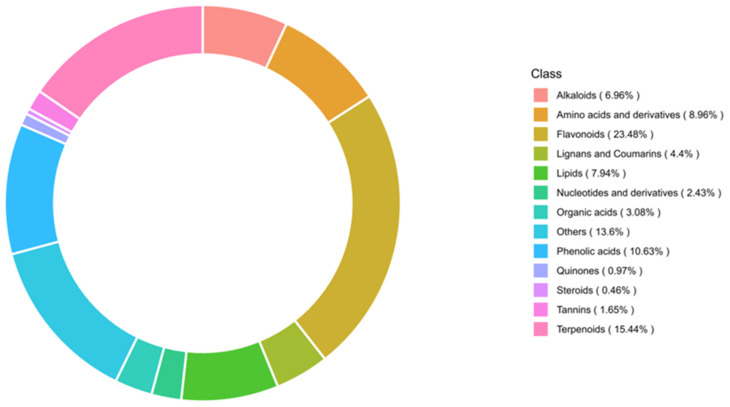
Metabolite category composition circular diagram.

To gain a clearer understanding of the metabolic patterns in four rhododendron species, primary and secondary metabolites in the samples were identified using the MWDB (Metware Database) via UPLC-MS/MS. Metabolite identification was based on MS/MS spectral matching and retention time alignment with the MWDB, and only metabolites with a coefficient of variation (CV) < 20% in QC samples were retained. A total of 3705 metabolites were detected, including 870 flavonoids, 572 terpenoids, 394 phenolic acids, 332 amino acids and derivatives, 294 lipids, 258 alkaloids, 163 lignans and coumarins, 114 organic acids, 90 nucleotides and derivatives, 61 tannins, 36 quinones, 17 steroids, and 504 other classes. The percentage distribution of each substance is shown in Figure 3. The most abundant detected flavonoid metabolites included chalcones, flavones, dihydroflavones, dihydroflavonols, anthocyanins, flavones, flavonols, flavanols, isoflavones, and other flavonoids (Table 1).

### 3.4. Metabolomic Profiling Reveals Systematic Differences Between Four Rhododendron Samples

**Figure 4:**
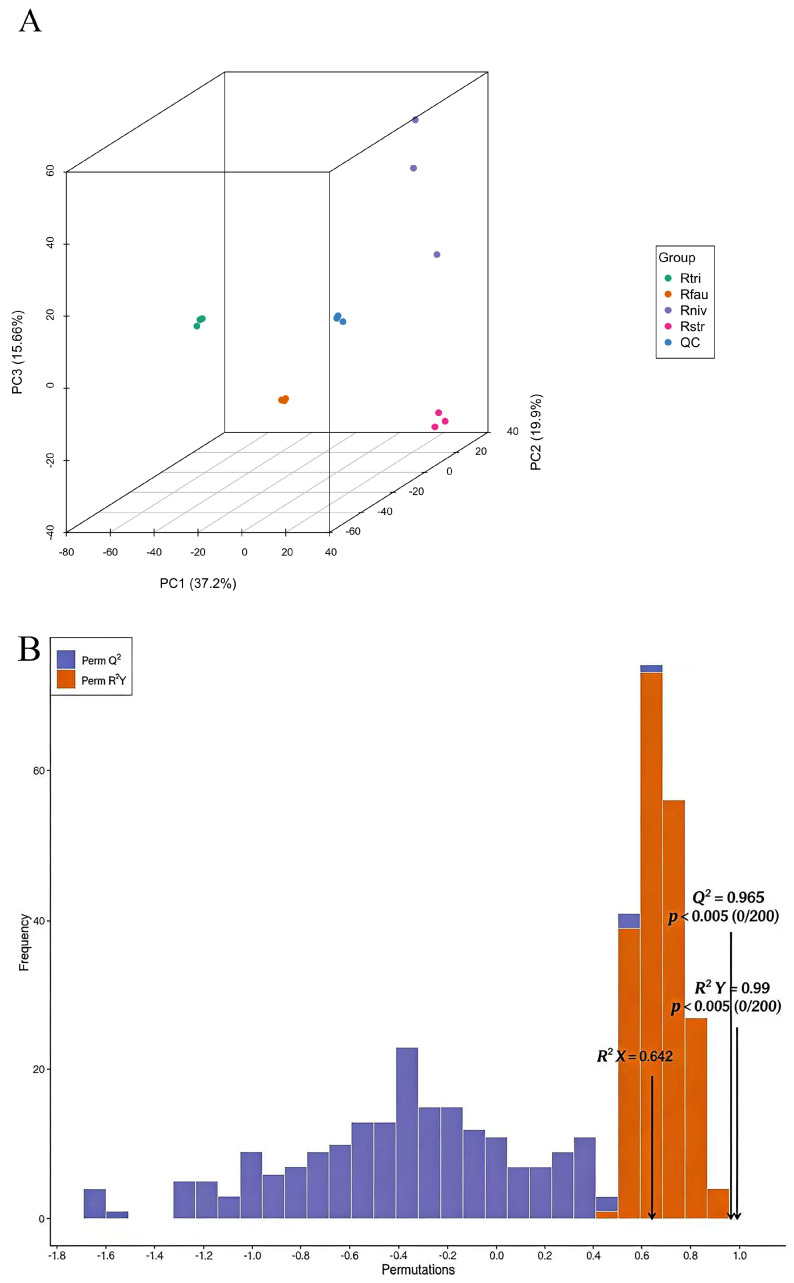
Systematic differences among four species of rhododendron. (**A**) Three-dimensional score plots of principal component analysis for each group. (**B**) OPLS-DA validation plot, *p* < 0.005 indicates optimal model.

To investigate the interrelationships between phenotypic variations in four rhododendron cultivars, an unsupervised PCA was first conducted. This study extracted principal components PC1, PC2, and PC3, accounting for 39.1%, 21.01%, and 16.35% of the variance, respectively. The analysis revealed tightly clustered replicate samples, indicating excellent experimental reproducibility and high reliability. A distinct separation trend was observed among the azalea cultivars, indicating differences in their overall metabolic profiles (Figure 4A).

To further identify variables with lower relevance, supervised orthogonal partial least squares discriminant analysis (OPLS-DA) was employed to distinguish overall differences among the four rhododendron metabolites (validated by 7-fold cross-validation and 200 permutation tests), with results presented in Figure 4B. Variables were screened based on the variable importance in the projection (VIP) values from the OPLS-DA model. The metabolites of *R.triflorum*, *R. faucium*, *R. nivale*, and *R. strigillosum* were distinctly separated, indicating significant differences in metabolic profiles across sample groups. The model quality parameters R^2^ and Q^2^ both exceeded 0.9, confirming the current OPLS-DA analysis model as stable and reliable with robust predictive capability, thereby facilitating the identification of potential differential metabolites.

### 3.5. Screening and Characterisation of Differential Metabolites

**Figure 5:**
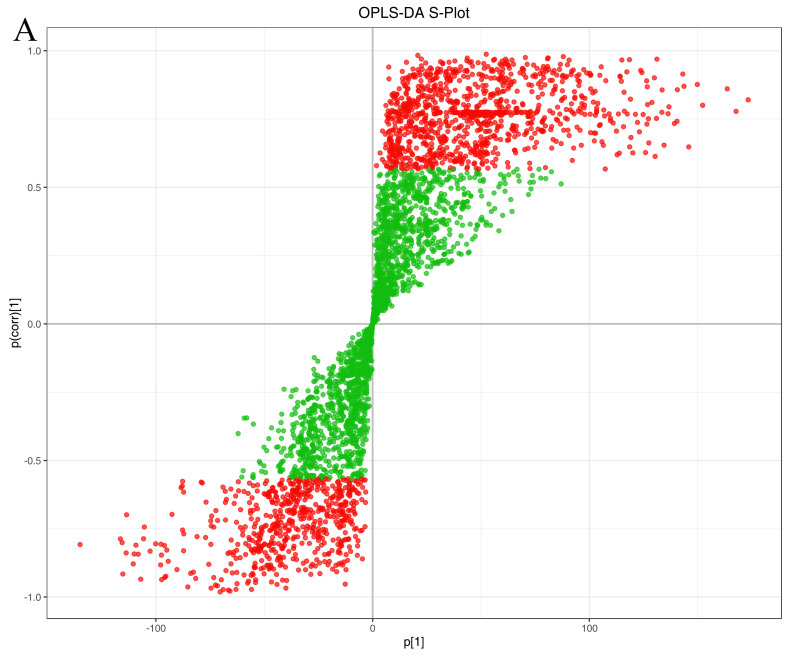

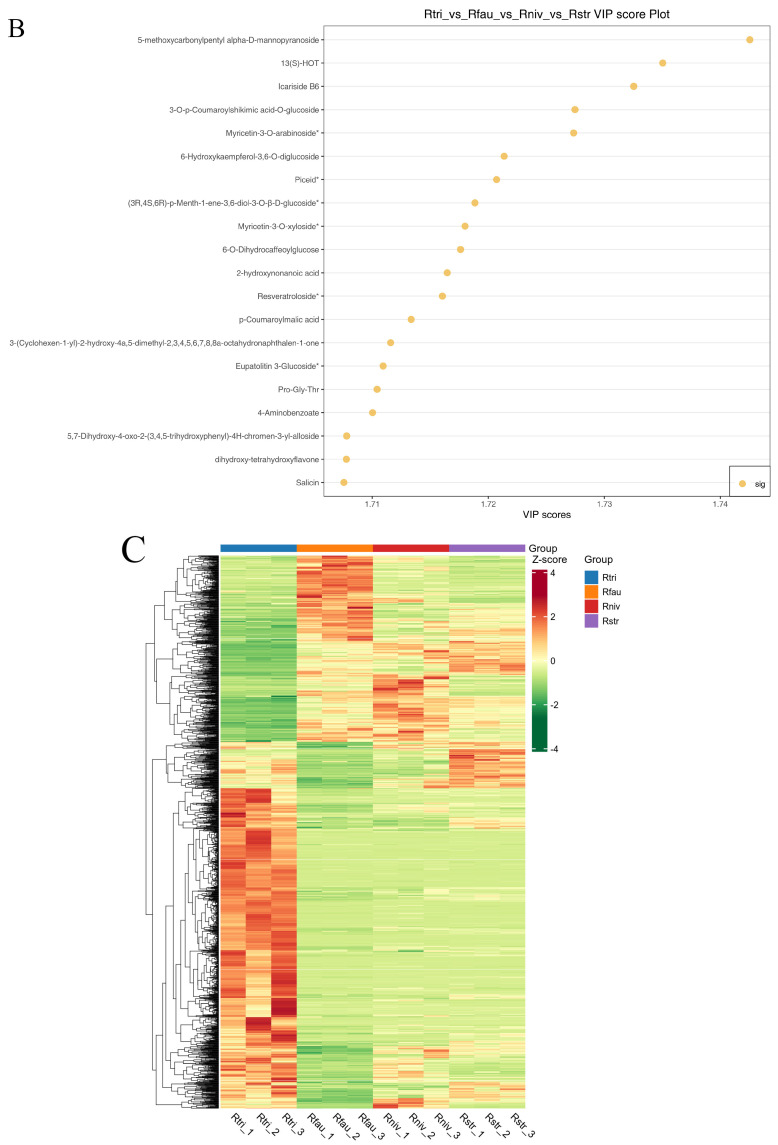

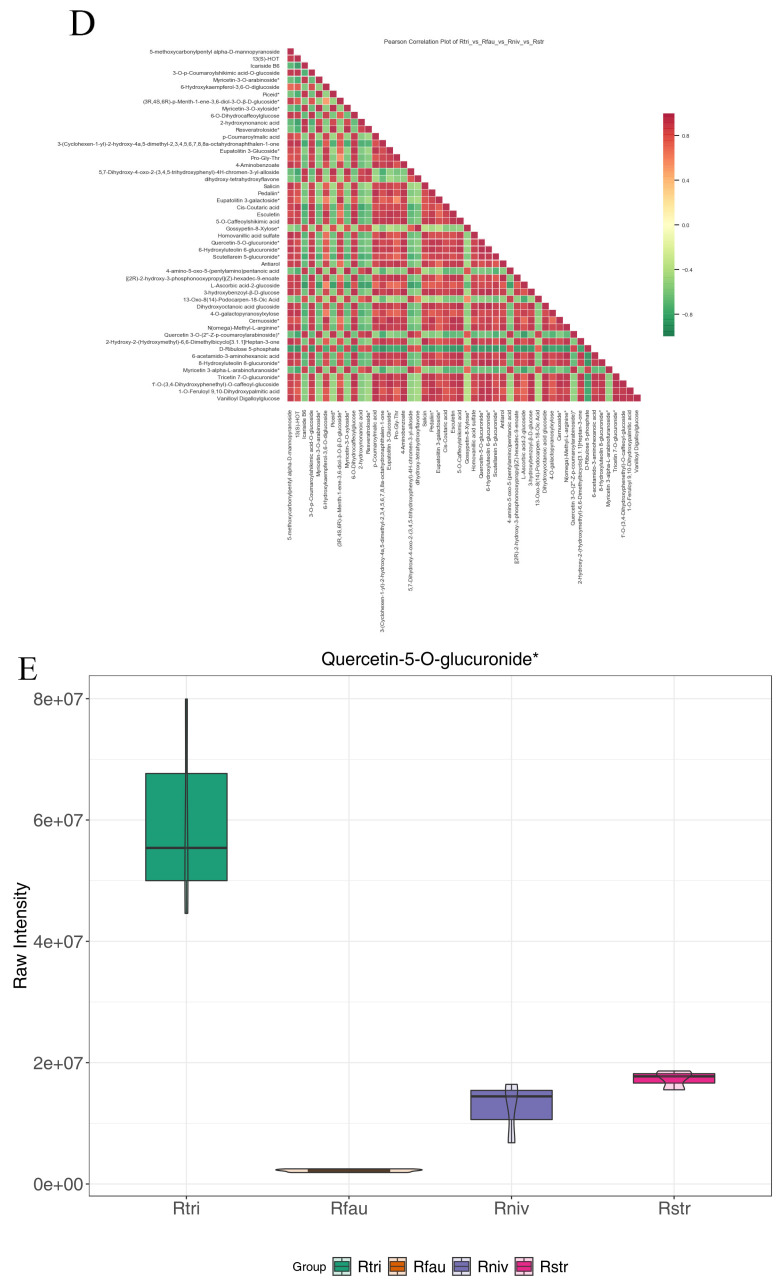

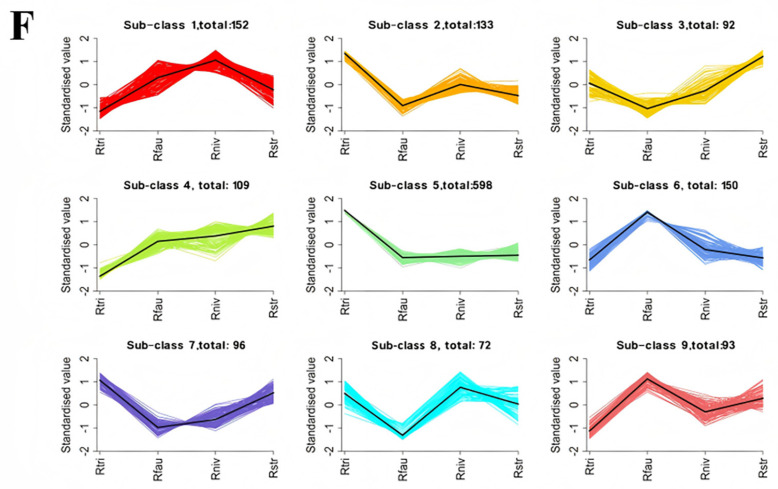
Screening and characterisation of differential metabolites. (**A**) S-plot diagram of OPLS-DA, where the horizontal axis represents the covariance between principal components and metabolites, and the vertical axis denotes the correlation coefficient between principal components and metabolites. Metabolites closer to the upper right and lower left corners indicate more significant differences. Red dots signify metabolites with VIP > 1, while green dots denote those with VIP ≥ 1. (**B**) VIP value plot of differentially expressed metabolites. (**C**) Clustering heatmap of differentially expressed metabolites, where red denotes high abundance and green denotes low abundance. (**D**) Correlation heatmap of differentially expressed metabolites, with varying colours representing the magnitude of Pearson’s correlation coefficient r. Red indicates strong positive correlation, green indicates strong negative correlation, and deeper colours denote higher absolute values of the correlation coefficient between samples. (**E**) Violin plot of differentially expressed metabolites. (**F**) K-means clustering diagram of differentially expressed metabolites. Nine subclasses of metabolite expression were identified. The black line shows the mean standardized expression trend for each subclass, while the colored lines represent individual metabolite expression trajectories within the subclass. The asterisks (*) following metabolite names indicate isomeric compounds that cannot be distinguished by tandem mass spectrometry (MS/MS). Structural annotations are based on accurate mass matching and MS/MS fragmentation patterns, but the precise isomeric form is not resolved.

In comparative metabolomics analyses involving multiple groups, accurately identifying and interpreting key variables responsible for inter-group differences constitutes the core process for discovering potential biomarkers. The relationship between covariance [p(1)] and correlation [p(corr)] in the OPLS-DA S-plot (Figure 5A) reflects the contribution of variables to the model and their correlation with principal components, enabling the visual identification of variables that significantly contribute to classification differences with high reliability. This facilitates efficient biomarker identification, enhances model interpretability and validation standards, and provides crucial leads for subsequent targeted validation and functional mechanism studies.

To establish reliable criteria for identifying differentially expressed metabolites, the VIP scores from the OPLS-DA model were combined with univariate statistical significance. Metabolites meeting the stringent threshold of VIP > 1 from the OPLS-DA model and *p* < 0.05 in one-way ANOVA followed by Tukey’s HSD post hoc test were deemed significantly altered. For multiple testing correction, the *p*-values were adjusted using the false discovery rate (FDR) method, and only metabolites with FDR < 0.05 were regarded as significantly different. The OPLS-DA model was validated with 200 permutation tests; the Q2 *p*-value was 0.02 (*p* < 0.05), indicating the model was robust, and no overfitting was observed. Comparisons were conducted among metabolites from the groups of *R. triflorum*, *R. faucium*, *R. nivale*, and *R. strigillosum*, revealing 1495 differentially abundant metabolites (40.35% of the total 3705 annotated metabolites) across the four rhododendron species. These included 338 flavonoids, 249 terpenoids, 177 phenolic acids, 119 amino acids and derivatives, 100 lipids, 117 alkaloids, 66 lignans and coumarins, 42 organic acids, 30 nucleotides and derivatives, 23 tannins, 13 quinones, 9 steroids, and 212 other classes. This reveals extensive metabolic reprogramming.

For the differentially expressed metabolites identified based on screening criteria in each subgroup comparison, the top 20 metabolites with the highest VIP values from the OPLS-DA model were selected for visualisation (Figure 5B). This figure clearly highlights key discriminating factors, such as 5-methoxycarbonylpentyl alpha-D-mannopyranoside and 13(S)-HOT, which possess the highest VIP scores, indicating their crucial role in driving the separation effect observed in the multivariate model.

Through unsupervised hierarchical clustering analysis, the abundance patterns of 1495 differentially expressed metabolites were visualised as a heatmap (Figure 5C). The global view revealed significant differences in metabolite content among the four rhododendron species, whilst the three replicates within each group showed minimal variation. Concurrently, cluster analysis of all metabolites revealed that *R. triflorum* exhibited the highest metabolite content, with over two-thirds of its metabolites surpassing levels in other species. This was followed by *R. Faucium*, *R. nivale*, and *R. strigillosum*.

Different metabolites exhibit synergistic or mutually exclusive relationships. Correlation analysis aids in quantifying the metabolic proximity between significantly different metabolites, thereby facilitating a deeper understanding of their mutual regulatory interactions during biological state transitions. To investigate potential interactions and synergistic regulatory mechanisms among differentially expressed metabolites in the four rhododendron species, this study employed Pearson correlation analysis on metabolites identified through screening criteria (Figure 5D). The resulting correlation matrix revealed significant positive correlations among metabolites within the same co-expression cluster in the heatmap, indicating their participation in shared biosynthetic pathways. Conversely, metabolites from opposing clusters frequently exhibited negative correlations, suggesting potential metabolic trade-offs.

To visually illustrate and compare the differences in abundance distribution among four rhododendron metabolites, violin plots were employed to further detail the distribution and statistical characteristics of key differentially expressed metabolites. Figure 5E reveals markedly distinct abundance profiles for quercetin-5-O-glucuronide across the four rhododendron species. The *R. triflorum* cohort exhibited the highest median intensity, followed by *R. strigillosum*, whilst *R. nivale* demonstrated lower signal intensity and a narrower distribution. Notably, *R. faucium* exhibited the broadest density distribution, spanning nearly the entire dynamic range, indicating substantial inter-individual variability. These divergent distribution patterns suggest quercetin-5-O-glucuronide may serve as a discriminating metabolite for the four rhododendron species, warranting further statistical validation.

To investigate the relative abundance trends of metabolites across four rhododendron species, all samples were screened to identify 1495 differentially expressed metabolites. These underwent unit variance scaling (UV) processing before K-means clustering analysis (Figure 5F). The results revealed nine distinct subclasses through K-means clustering. Subclass 5 (*n* = 598, approximately 46% of total metabolites) emerged as the absolute dominant theme, indicating the presence of a core metabolic phenotype exhibiting a pronounced “one dominant, multiple auxiliary” structure. The absence of distinct stratification in the figure indicates that K-means successfully partitioned the data into discrete, non-overlapping modules without empty sets or isolated clusters. The contour coefficient distribution was balanced (mean = 0.43), demonstrating robust clustering stability.

### 3.6. Functional Annotation and Pathway Analysis of Differentially Expressed Metabolites

To interpret the biological significance of the 1495 differentially expressed metabolites identified, this study employed systematic functional annotation and pathway enrichment analysis to precisely pinpoint specific biochemical pathways and processes undergoing significant reprogramming across four rhododendron cultivars. Functional annotation of all differential metabolites using the KEGG [13] and MetMap [14] databases revealed that the variations in azalea metabolites involved multiple metabolic pathways. A total of 128 pathways were statistically screened, with the highest enrichment observed in metabolic pathways (36.71%), biosynthesis of secondary metabolites (21.27%), and biosynthesis of cofactors (6.33%). Classifying these 128 pathways revealed that 37 belonged to the MetMap database and 91 to the KEGG database. At the primary classification level, 86 pathways fell under metabolism, 3 under environmental information processing, 1 under genetic information processing, and 1 under cellular processes.

**Figure 6:**
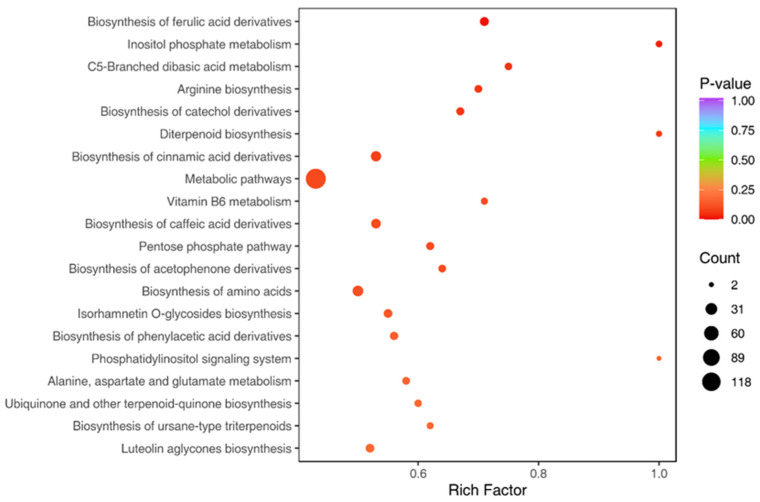
Differentially enriched metabolic pathway enrichment plot. Metabolic pathway names were derived from the KEGG (116.1) and MetMap™ (MetMap500) databases. Dot colour indicates the magnitude of the *p*-value (redder dots denote more significant enrichment), and dot size represents the number of differentially enriched metabolites. The rich factor is defined as the ratio of differentially expressed metabolites to the total annotated metabolites within each pathway.

To further identify the most significantly affected metabolic pathways and elucidate the functional significance of differentially expressed metabolites, pathway enrichment analysis was conducted using hypergeometric tests to calculate *p*-values. As depicted in the pathway enrichment diagram (Figure 6), the top 20 pathways selected by *p*-value are visualised, with the “Metabolic pathways” pathway exhibiting the most significant enrichment. This indicates that these alterations represent coordinated metabolic shifts within a specific biological context, rather than random fluctuations.

## 4. Discussion

### 4.1. Metabolic Diversity and Ecological Implications

In this study, a metabolomic approach was employed to comparatively analyse the differences in metabolites among four Rhododendron species, namely *R. triflorum*, *R. faucium*, *R. nivale*, and *R. strigillosum*. Widely targeted metabolomic analysis was performed using UPLC-MS/MS technology, leading to the identification of a total of 3705 metabolites, which were classified into 13 categories. These included flavonoids (870 compounds), terpenoids (572 compounds), phenolic acids (394 compounds), amino acids and their derivatives (332 compounds), lipids (294 compounds), alkaloids (258 compounds), lignans and coumarins (163 compounds), organic acids (114 compounds), and nucleotides and their derivatives (90 compounds), among others. Among these metabolites, 1495 exhibited significant differences across the four Rhododendron species, reflecting inherent biochemical variations. Such differences may be associated with species-specific genetic backgrounds, niche differentiation, or variations in environmental adaptability.

The metabolic diversity of the four species of rhododendrons is likely not only a consequence of taxonomic divergence but also a manifestation of ongoing ecological adaptation strategies. The severe environmental stress in the Xizang region, such as intense ultraviolet rays, fluctuating temperatures, and hypoxia at high altitudes, can cause significant differences in the metabolism of plants [15,16]. Li-Juan Deng et al. conducted non-targeted metabolic analysis of *U. hyperborea* and *U. dioica* from different locations on the Qinghai–Xizang Plateau and found that the metabolic differences between them were mainly reflected in the content of carbohydrates and phenylpropanol. The differential metabolites of the same species at different altitudes were mainly enriched in carbon metabolic pathways and lipid metabolic pathways. These findings indicate that the high-altitude adaptation mechanisms of sympatric species differ, providing new insights into adaptive metabolic strategies [17]. Significant differences in abundant flavonoid and terpenoid metabolites may represent divergent evolutionary schemes in response to these environmental challenges. For example, Guoqi Xu et al. analysed *Meconopsis horridula*, a unique species on the Qinghai–Xizang Plateau, using transcriptome, metabolome, and microbiome analyses. This work revealed a high-altitude UV-B adaptation strategy centred on flavonoid biosynthesis, and improved our understanding of UV-B tolerance in alpine plants. It also provides potential resources for crop improvement [18]. In their study on *Codonopsis pilosula* var. modesta, Nannf. (CPM), Zi-Xia Wang et al. found that CPM could significantly up-regulate the gene expression levels of seven key enzymes in the triterpenoid biosynthesis pathway. The ecological adaptation significance of triterpene accumulation under the combined stress of low temperature and drought on the plateau was revealed for the first time [19]. Therefore, the metabolic differentiation phenomenon presented in this article may support the niche differentiation and coexistence mechanisms of these Rhododendron species in this biodiversity hotspot area.

An important limitation in interpreting metabolic differences should be noted: *R. triflorum* was collected from a site with a distinct elevation, while *R. faucium*, *R. nivale*, and *R. strigillosum* were sampled from nearby locations with similar environmental conditions. These four species therefore did not experience fully shared macroenvironments, and such elevation differences mainly influence comparisons involving *R. triflorum*. Consequently, the observed metabolic divergence in *R. triflorum* may reflect both species-specific characteristics and adaptive responses to different elevation-related environments, which should be taken into account when comparing metabolic profiles between *R. triflorum* and the other three species.

### 4.2. Key Differential Metabolites and Metabolic Pathways

In-depth analysis of differential metabolites can reveal the specific biochemical mechanisms underlying the observed diversity. Rhododendron species contain diverse classes of metabolites, and analysis of their principal compounds reveals their significant roles in morphology and physiology [20,21].

Flavonoids, as the most abundant and diverse metabolites in Rhododendron species, play a significant role in anti-inflammatory, antioxidant, and UV defence mechanisms [22]. Myricetin 4 (Myr) exerts an inhibitory effect on photoaging in mouse skin and human keratinocyte (HaCaT) cells. Myr enhances antioxidant stress activity in both skin and cells, reduces oxidative product levels, and suppresses NF-κB signalling pathway activity, thereby achieving anti-photoaging effects on the skin [23,24]. The glycoside form of Myricetin, Myricetin 3-O-β-galactopyranoside, has a protective effect on UVA-induced skin cell damage by inhibiting the MAPK/AP-1 pathway and activating the TGFβ/Smad signalling pathway, thereby slowing down collagen degradation and inflammatory responses [25]. Quercetin and kaempferol, among flavonoids, can also influence the colour of plants by regulating pigmentation. In purple tea leaves, co-pigmentation of related derivatives is the key factor determining leaf colour variation. Their synthesis is synergistically regulated by structural genes (e.g., FLS, DFR) and corresponding regulatory networks. It indicates that the polyhydroxyflavonoid metabolic flow competes with anthocyanins for substrates, directly affecting the depth of colour [26]. The unique floral fragrance of Rhododendron species stems from the abundance of terpene volatiles. Yi Qin et al. found in their studies on the fragrant Rhododendron yunjin (YJ) and the odourless ‘Nova Zembla’ (NW) that (-)-myrtenol, linalool, α-pinene, myrtenyl acetate, and terpineol are the characteristic sweet-scented terpenes of YJ. The structural gene RfFDPS has been confirmed as the “switch” for monoterpene-sesquiterpene distribution. Overexpression reduces monoterpenes and increases sesquiterpenes, while silencing is the opposite. This has, for the first time, revealed the metabolism–gene bilayer regulatory mechanism of terpene floral fragrance in rhododendrons [27]. Yang Guoxia et al. investigated the petals of *Rhododendron yunliensis* and *Rhododendron nuva* at different developmental stages. They found that the expression levels of TPS1, TPS4, TPS9, TPS10, TPS12, and TPS13 in R. yunliensis were significantly positively correlated with terpene content, suggesting that these six genes may contribute to regulating its floral fragrance [28].

Metabolic pathways are reaction chains within cells formed by multiple enzymatic reactions connected in a specific sequence, which can convert starting substances into end products and simultaneously generate or consume energy (such as ATP, NADPH, etc.). As the most enriched pathways among the four rhododendron plants, they play a crucial role in regulating primary and secondary metabolism. Research has found that the key enzyme gene RfHMGR1 of the MVA pathway in rhododendron (*R. fortunei*) is highly expressed in the petals. Its overexpression increases the content of linalol (the main component of floral fragrance) in the transgenic plants by 2.3 times, confirming that this pathway directly determines the intensity of floral fragrance [29]. Sulin Wen et al. systematically mapped the synthetic spectrum of secondary metabolites of Rhododendron bailiense in Guizhou at the genomic level for the first time. By integrating metabolomics and comparative genomics, they identified multiple key pathways, providing a theoretical basis for drug development [30].

### 4.3. Research Value and Future Research Directions

The diverse metabolites and key biomarkers identified in this study hold certain practical value, providing a preliminary reference for the identification of the four tested rhododendron species—*R.triflorum*, *R. faucium*, *R. nivale*, and *R. strigillosum*—based on their metabolic profiles under the tested growth conditions, while also offering a reference for relevant research on species identification within the Rhododendron genus. The identified key flavonoids and terpenoids offer direct translational prospects for the horticultural industry, serving as markers for breeding programmes aimed at enhancing floral colour saturation or developing novel floral fragrances. Moreover, the discovery of potent antioxidants accumulated with species specificity and potential novel metabolites opens new avenues for bioprospecting. These compounds represent ideal research subjects for potential applications in pharmaceuticals, nutritional supplements, or natural cosmetics, adding economic incentives for safeguarding this precious biodiversity [31].

Future research directions must adopt an integrated multi-omics strategy. Simultaneous sequencing of transcriptomes and metabolomes for these species is crucial for identifying key regulatory genes and transcription factors governing metabolic landscapes. Functional validation through gene knockout or heterologous expression will be a pivotal step in definitively establishing the biosynthetic pathways of these distinctive metabolites and their ecological functions. This integrated research approach will shift our understanding of Xizangan Rhododendron species from correlational studies towards causal revelations, comprehensively unlocking their genetic and biochemical mysteries.

## 5. Conclusions

Widely targeted metabolomics analysis in this study confirmed distinct species-specific metabolic features among four Rhododendron species from Linzhi, Xizang, with significant interspecific differences in metabolite compositions—most notably in flavonoid and terpenoid biosynthesis pathways. These differences not only realised effective discrimination of the four species but also revealed that genetic background, rather than the similar macro-environment, may play a more dominant role in shaping metabolic profiles, acknowledging that the influence of local microenvironment could not be completely distinguished due to the current sampling scheme. The differential accumulation of key metabolites is directly linked to physiological functions such as anti-inflammation, antioxidant activity, and ultraviolet protection, underscoring the adaptive importance of metabolic variations. This research deepens the understanding of the chemical basis for biodiversity and environmental adaptation of Rhododendron in the Qinghai–Xizang Plateau, provides valuable chemotaxonomic resources, and lays a foundation for future applications, including horticultural breeding of novel ornamental traits and exploration of high-value natural products with industrial potential.

## Figures and Tables

**Table 1:** Flavonoid metabolite secondary classification.

Secondary Classification of Flavonoids	Quantity	Percentage Share (%)
Chalcone	45	5.17
Aurones	15	1.72
Flavanones	72	8.28
Flavanonols	15	1.72
Anthocyanidins	45	5.17
Flavones	224	25.75
Flavonols	307	35.29
Flavanols	62	7.13
Isoflavones	41	4.71
Other Flavonoids	44	5.06

## Data Availability

The data that support the findings of this study are available within the article and its Supplementary Materials. No new data have been deposited in a public database, and all relevant data are presented in the main text and Supplementary Files, as no prior publication or external data sharing has been conducted for this study.

## Supplementary Materials

The following supporting information can be downloaded at: https://www.mdpi.com/article/10.3390/metabo16040226/s1, Table S1: All detected metabolites information; Table S2: K-means Clustering Analysis of All Metabolites; Table S3: Differentially Accumulated Metabolites Information.
