# Supplementary material for: Metabolomics Based on UPLC-MS/MS Revealed the Metabolic Differences Among Four Species of Rhododendrons in Linzhi, Xizang
Source: Metabolites. 2026 Mar 30;16(4):226. doi: 10.3390/metabo16040226 (PMC13117825; doi:10.3390/metabo16040226)
Supplement: Supplementary file 1 [file metabolites-16-00226-s001.zip › Supplementary documents/Supplementary References/2.Geng Y (2010) Investigation and taxonomic study on Rhododendron resources in Linzhi Prefecture, Xizang..pdf]

西藏林芝地区杜鹃花属植物资源考察及分类学考证

耿玉英
(中国科学院植物研究所，北京 100093)

摘　要：《中国植物志》记录西藏自然分布的杜鹃花种类有180多种，其中有许多种为当地特有。尤其是西藏东南部是中国杜鹃花分布最丰富的地区之一，与云南、四川共同形成世界杜鹃花分布中心。文章是对林芝地区部分杜鹃花资源的考察记录，同时也对部分种类的分类问题提出讨论。

关键词：西藏；林芝地区；多雄拉；杜鹃花

西藏是拥有中国杜鹃花属(*Rhododendron*)植物种类最多的地区之一，独特的地理位置和气候条件提供了特别丰富的植物区系，也为杜鹃花生长提供最佳生境，与云南、四川共同形成世界杜鹃花分布中心。据《中国植物志》记载，分布在西藏的杜鹃花植物有180多种，主要分布在东南部，它们中有许多为当地特有种，这对研究中国乃至喜马拉雅地区杜鹃花属植物都有十分重要的意义。

英国人弗兰德.金登-沃德(Frank Kingdon-Ward，1885～1958)是在西藏的较早植物采集者之一，在他跨越45年、22次的采集探险旅行中，曾多次涉足我国西藏东南部，采集了大量的杜鹃花标本和种子，原产西藏东南部的朱砂杜鹃(*R.cinnabarinum*)和毛喉杜鹃(*R.cephalanthum*)及黄杯杜鹃(*R.wardii*)都是他最先发现并采集。与其同期或稍后期在西藏有大量杜鹃花采集的是罗兰·库珀(Roland Edgar Cooper，1890～1962)以及兰德·勒德洛(Frank Ludlow，1885～1972)和乔治·谢里夫(George Sherriff，1898～1967)，他们的采集都无疑为西方园林提供了大量的杜鹃花资源，也是分类学家们新种描述的重要依据，在我国有广泛分布的黄杯杜鹃(*R.wardii*)和分布于西藏的广口杜鹃(*R.ludlowii*)、红钟杜鹃(*R.sherriffii*)等就是后人为了纪念他们分别以其姓氏命名的。在《中国植物志》的编写中他们的采集也是重要的依据。

至20世纪50年代，我国植物分类学者对西藏杜鹃花的采集有过一些记录，但大规模进行是中国科学院在上世纪70年代组织的青藏高原考察，丰富的材料成为今天我国有关学者研究的重要材料。但由于部分种类标本质量和数量、一些在干标本不复存在的特征等问题；同时也由于在自然条件下，杜鹃花多少具"群居"的特性，几种乃至数10种生长在一个相对窄小的地域，同亚组间、同组间甚至不同组间的杂交时常发生，产生了众多"似是而非"的中间类型，这也为正确的鉴定和分类研究带来了一些困难，需要进一步的野外考察和研究。本文是对于林芝地区部分杜鹃花资源的考证以及对其中的几种分类问题提出讨论。

与70年代林芝的建设规模相比，当年部分标本采集地已经永远被埋藏在城市建设的混凝土下，但在林芝附近及周围仍然不乏丰富的杜鹃花种类，主要有雪山杜鹃(*R aganniphum* var.*aganniphum*,色季拉山东西坡，彩图1)及其变种裂毛雪山杜鹃(*Raganniphum* var.*schizopeplum*，色季拉山等，彩图2)，钟花杜鹃(*R.campanulatum*,色季拉山东坡，雪坝山等)，光蕊杜鹃(*R.coryanum*,色季拉山东坡)，鳞腺杜鹃(*R.lepidotum*,农牧学院后山等)，蜿蜒杜鹃(*R.bulu*,比日神山等，彩图3)，猴斑杜鹃(*R.faucium*,鲁浪等，彩图4)，草莓花杜鹃(*R.fragariiflorum*，色季拉山东坡)，硬毛杜鹃(*R.hirtipes*,色季拉山东、西坡等)，鲁浪杜鹃(*R.lulangense*,色季拉山东、西坡等,彩图5)，疏花杜鹃(*R.dignabile*,色季拉山东坡)，雪层杜鹃(*R.nivale* var.*nivale*，彩图6)，林芝杜鹃(*R.nyingchiense*，彩图7)，山育杜鹃(*R.oreotrephes*,色季拉山东坡，彩图8)，多变杜鹃(*R.selense* var.*selense*,色季拉山西坡等，彩图9)，林芝光柱杜鹃(*R.tanastylum* var.*lingzhiense*,色季拉山东坡，彩图10)，三花杜鹃(*R.triflorum* var.*triflorum*,色季拉山东、西坡等，彩图11-13)，柳条杜鹃(*R.virgatum*,色季拉山东披)，黄杯杜鹃(*R.wardii* var.*wardii*,色季拉山东、西坡等，彩图14)。

地处林芝地区的多雄拉山是世界上矮小杜鹃花种类最丰富区域。弗兰德·金登曾描述此地是由杜鹃花覆盖的"仙境"。山顶终年积雪、潮湿、大雾笼罩，是杜鹃花最理想的生境。在多雄拉北坡从海拔3200～3600m，分布有丰富的三花杜鹃、猴斑杜鹃和薄毛紫玉盘杜鹃(见彩图15)，3600～4000m几乎被矮小杜鹃花覆盖(见彩图16、彩图17)；主要种类有草莓花杜鹃(*R.fragariiflorum*)，矮小杜鹃(*R.pumilum*)，美被杜鹃(*R.calostrotum*，彩图18)，小叶美被杜鹃(*R.calostrotum* var.*calciphilum*)，毛喉杜鹃(*R.cephalanthum*)，毛冠杜鹃(*R.laudandum* var.laudandum，彩图19)及变种疏毛冠杜鹃(*R.laudandum* var.*temoense*)，工布杜鹃(*R.kongboense*)，髯花杜鹃(*R.anthopogon*，彩图

作者简介：耿玉英（1957—），女，高级工程师；主要从事中国杜鹃花迁地保护和分类学研究。

项目来源：国家科技基础条件平台工作重点项目(2003DKA3N023，2004DKA30510，2005DKA21401)——Plant Specimen Digitization and Chinese Virtual Herbarium Establishment(2003DKA3N023，2004DKA30510，2005DKA21401)。

中国知网 https://www.cnki.net

20)，单花杜鹃(*R.uniflorum*)。

紫背杜鹃(*R.forrestii*，彩图21)是根据西方著名采集者乔治·福雷斯特(*G.Forrest*)姓氏而定名，这是弗兰德·金登在多雄拉最喜爱杜鹃花，为矮小的灌木，株高6～25cm左右，相对于植株的大小而言，该种的叶和花都较大。花序有花1～2朵，深红色、管状钟形和腊质的花冠具有极高的观赏性，是西方园林许多杂交种的亲本，与紫背杜鹃混生有大量的云雾杜鹃(*R.chamaethomsonii*)，在无花期几乎两种没有区别，有研究者曾将后者作为紫背杜鹃的同种异物而归并，但其植株生长稍高，20～60cm，花序有花2～4(5)朵，花色除深红色还有深粉红色等区别，在这里植株较高大的种类还有淡钟杜鹃(*R.lanatoides*，彩图22)，盘萼杜鹃(*R.parmulatum*，彩图23)，火红杜鹃(*R.neriiflorum*)以及藏布雅容杜鹃(*R.charitopes subsp. Tsangpoense*，彩图24)，株高在50cm～1m。盘萼杜鹃出现较大的变异，尤其在花冠的颜色粉红色、白色但先端粉红色或白色，花冠内面的斑点的变化是仅在基部扩展至中部至整个上部花瓣内面，这种现象出现在同一植株的不同花序。

弯果杜鹃(*R.campylocarpum* var.*campylocarpum*，彩图25)主要分布在多雄拉的北坡，从海拔3600～3900m，其黄色、淡黄色，偶而出现的白色花朵几乎覆盖了大部分山坡，随海拔由低至高变化，植株高度从仅有几十厘米至2m以上，其形态特征与黄杯杜鹃相似，但花柱洁净无腺体亦无毛可以区别；《中国植物志》记载本种花色仅有黄色，国外文献记载有白色花，这在多雄拉也有发现；变种美丽弯果杜鹃(*R.campylocarpum* subsp.*Caloxanthum*)与原变种的区别仅是花丝无毛，在多雄拉分布较原变种稍高，多在3800～3900m以上，植株更矮小。

《中国植物志》记录毛枝杜鹃亚属有6种，多雄拉分布有3种，即弯月杜鹃(*R.mekongense*，彩图26)及其变种红线弯月杜鹃(*R.mekongense* var.*rubrolineatum*)，糙毛杜鹃(*R.trichocladum*)和显绿杜鹃(*R.viridescens*，模式产地)。

多雄拉南坡(那格至汉密)是大叶杜鹃花的天堂，茂密的裸子植物林下，大叶类的杜鹃花成为优势种群，它们多为高大灌木或乔木，主要有大叶杜鹃(*R.grande*，彩图27)，凸尖杜鹃(*R.sinogrande*，彩图28)，墨脱杜鹃(*R.montroseanum*)，翘首杜鹃(*R.protistum*)，优秀杜鹃(*R.praestans*)，夺目杜鹃(*R.arizelum*)，粘毛杜鹃(*R.glischrum*，彩图29)，长粗毛杜鹃(*R.crinigerum*)，小灌木状的管花杜鹃(*R.keysii*，彩图30)，多生在乔木树干上的泡泡叶杜鹃(*R.edgeworthii*)，及矮小灌木绿柱短花杜鹃(*R.brachyanthum* subsp. *Hypolepidotum*)。

以上仅是林芝地区部分杜鹃花资源的记录，对于西藏东南部尤其是地处印度边界地区的植物区系远没有像在我国其他地区那样进行过非常彻底的采集研究，对于考察中发现的一些分类问题，将在下面提出讨论：

(1)《中国植物志》记载三花杜鹃亚组的种类在我国有23种，仅有两种分布至西藏，即山育杜鹃和三花杜鹃，后者是西藏东南部最常见的种类，分布海拔2500～3700m，在野外的鉴别特征主要是：常绿小灌木，茎光滑，紫红色或浅褐色，皮薄片状脱落；叶片多为卵圆形或长圆形，背面灰白色或淡绿色，花序顶生，2～3(4)花，花冠阔漏斗状钟形，有时近碟形，淡黄色，淡黄色–淡粉红色，上部裂片内面具较花瓣更深色的斑点(见彩图11–13)。

我国有关学者根据其花序多花的特征，于1982年描述新变种云南三花杜鹃(*R.triflorum* var.*multiflorum*)，后者花序具3～5(或更多)花；1937年，英国人P.Hutchison根据弗兰德·金登–沃德在西藏东南部采集的材料描述变种红色三花杜鹃(*R.triflorum* var.*mahoganii*)，与原变种的区别是花冠内斑点为紫红色；由于本种花色变化的连续性，红色三花杜鹃不被多数学者接受，但在西方园林界中仍以“Mahoganii Group”作为与黄色花植株的区别。在野外三花杜鹃花序有3～5花常见，仔细观察发现花序有花的多少与花冠内斑点的颜色有一定联系：花冠为淡黄色、斑点为淡绿色或黄绿色，花序多为3花；斑点为淡紫色或淡红紫色花序为4～5或多花。云南三花杜鹃的模式标本和原文都没有提及花色的问题，单纯的花序多花不能作为与原变种的区别特征，另外红色三花杜鹃原文没有指定模式标本，为不合格发表，因此也是一个不合法名。

(2)鲁浪杜鹃是我国学者于1986年描述的新种，原作者将其作为银叶杜鹃亚组的成员。原文没有提及果特征，但在标本馆(PE)几份采自同地的果期标本(易77168，李渤生等6205)叶形及被毛特征与指定的鲁浪杜鹃模式标本特征相同，但果的大小、弯曲似镰刀的特征都与镰果杜鹃亚组种类所具有的特征相似；查看鲁浪杜鹃的模式标本，其叶形态、毛被颜色和特征以及花各部分特征都与薄毛紫玉盘杜鹃(*R.uvariifolium* var.*griseum*)更相似，后者也是根据弗兰德·金登在西藏东南部采集的标本描述，后来的研究中有学者已经将其作为紫玉盘杜鹃(*R.uvariifolium* var. *uvariifolium*)的同物异名归并(《中国植物志》接受这种归并)，但由于其叶形状和叶背毛被的区别，部分学者仍作为接受名；从薄毛紫玉盘杜鹃的模式标本野外观察，与原变种存在明显的区别，而鲁浪杜鹃(见彩图5)与薄毛紫玉盘杜鹃(见彩图15)的联系不足作为种间区别，可能是其同物异名。

林芝杜鹃是根据采自林芝雪坝山的标本而描述，在其模式产地和色季拉山东、西坡海拔3600～4700m都有分布，本种叶形态特征和花色、花各部特征都与毛冠杜鹃(*R.laudandum*)相似，与变种疏毛冠杜鹃(*R.laudandum* var. *temoense*)更近，与前者的区别是花色较淡，通常为白色或淡粉红色，子房无毛；与后者的区别仅仅是叶芽鳞早落。疏毛冠杜鹃是根据弗兰德·金登在西藏多雄拉采集的材料描述。在多雄拉毛冠杜鹃及其变种疏毛冠杜鹃都有分布，后者在开花期叶芽鳞的宿存情况有极大的变化，在同株不同小枝上有或无宿存的叶芽鳞存在，因此林芝杜鹃(见彩图7)与疏毛冠杜鹃的的关系需要考证(见彩图19)。

(3)硬毛杜鹃(见彩图31、彩图32)为西藏东南部特有种，也是由弗兰德·金登在1924年从西藏南部发现并采集。1930年Tagg作为新种描述种时没有指明模式标本，随后于1931

中国知网 https://www.cnki.net

年正式发表。根据植株(尤其是在幼枝和叶柄等部位)密被腺状刚毛的特征，被归于具有相似特征的硬刺杜鹃系的粘毛杜鹃亚系(Barbatum Series, Subseries Glischrum，即后来的粘毛杜鹃亚组subsect. Glischra)，后来有研究者因为其叶形更接近多变杜鹃系(Series Selensia)将其移入多变杜鹃亚组(subsect. Selensia)，《中国植物志》接受这一变动。但在我看来，除了花序少花外(硬毛杜鹃有花4～5朵，而粘毛杜鹃亚组的种类花都在6朵以上)，硬毛杜鹃植株各部的特征更近粘毛杜鹃亚组，相对于叶形毛被特征更具有分类意义，建议将其保留在粘毛杜鹃亚组。

(4)管花杜鹃(*R. keysii*，彩图30)是喜马拉雅地区一常见种，花序3～5花，常常是多花序簇生枝顶或近顶部，花冠长管状，厚肉质，橙红色、肉红色或深红色，1937年Cowan依据罗兰·库珀及兰德·勒德洛在西藏东南部采集的标本描述新种肉红杜鹃(*R. igneum*)，与管花杜鹃的主要区别是叶较薄，长约3～7cm，叶背面鳞片相距稍疏(相距为其直径的2倍，而在管花杜鹃叶长为4～10，有时达15cm，叶背面鳞片相距为其直径的1倍左右)及花序少花(每花序2～3花，多花序生枝顶)等，随后作者本人将其归作管花杜鹃的同物异名；1986年我国学者根据青藏考察队采自墨脱那格的标本描述新种薄叶管花杜鹃(*R. tenuifolium*)，其与管花杜鹃的主要区别是叶较大而薄，叶背面鳞片稍疏(相距为其直径的1～2倍)等，野外观察同一植株叶片大小和质地、叶背面鳞片的疏密及花部被毛的特征都有变化，三种的分类地位值得考虑。

(5)猴斑杜鹃(*R. faucium Chamb*，彩图4)是1980年根据在英国爱丁堡皇家植物园栽培植株而描述的新种，是由兰德·勒德洛和乔治·谢里夫等从西藏林芝县采集(《中国植物志》记录模式标本采自西藏波密，但标本记录采集地为"Pome Province, Layoting, Tongyuk Chu"，是指今林芝县的东久乡，因此本种的模式产地应该为林芝县)。在猴斑杜鹃发表前，相关材料(标本和活植物)都被定名为粉果杜鹃(*R. hylaeum*)，在现有的标本中二者仍被混淆。两种的茎光滑，淡褐色或灰褐色，皮片状脱落，叶背面毛被等都相似，但猴斑杜鹃仅限分布在西藏东南部，叶较粉果杜鹃小，子房密被腺毛等特征与之区别明显，而后者叶较大，基部多为圆形，子房洁净无毛，云南西北部、缅甸东北部及相邻的西藏东南部。原文及《中国植物志》记录猴斑杜鹃的花色为乳白色或粉红色，而粉果杜鹃的花色更深，但在模式采集者的描述表明猴斑杜鹃也有深色的花冠，这在多雄拉得到了证实(见彩图22)。

由于杜鹃花丰富多样性和其在自然条件下极易杂交的特性，还不能对考察中感到疑问的所有种类或标本在这里全部提出讨论。这一地区还有许多区域需要进一步的考察研究，相信一定会有更多、更有意义的发现。

【注】 文中彩图见封二、封三、封底。

参考文献：

[1] 方瑞征. 云南杜鹃花属新分类群[J]. 云南植物研究, 1982, 4(3):249-253.

[2] Fang Rui-zheng. Flora Reipublicae Popularis Sinicae[M]. Beijing:Sciense Press, 1999.

[3] Fang Rui-zheng, Yang Han-bi. Flora of China[M]. Beijing: Sciense Press, 2005.

[4] Hu Lin-Zhen, Fang Ming-Yuan. Flora Reipublicae Popularis Sinicae[M]. Beijing:Sciense Pres, 1994.

[5] Hu Lin-Zhen, Fang Ming-Yuan. Flora of China[M]. Beijing: Sciense Press, 2005.

[6] 闵天禄. 滇藏常绿杜鹃亚属的修订[J]. 云南植物研究, 1984, 6(2):141-171.

[7] Chamberlain D. F. A revision of Rhododendron, II. subgenus Hymenanthes. Notes from the Royal Botanic Garden Edinburgh, 1982, 39:209-486.

[8] Cullen, J. A Revision of Rhododendron 1. sbuges Rhododendron sections Rhododendron&Pogonanthum. Notes from the Royal Botanic Garden Edinburgh, 1980, 39:1-207.

[9] Davidian, H. H. A Review of Rhododendrons in their series VII. The Triflorum series, Rhododendron and Camellia Yearbook (The Royal Horticultural Sociaety), 1962, 17: 164-165.

[10] Stevenson, J. B. The species of Rhododendron[M]. The Rhododendron Society Edinburgh, 1930.

Rhododendrons Notes in Linzhi

GENG Yu-ying

Abstract: Tibet is the one of the most important places for Chinese *Rhododendrons*. More than 180 species are recorded in the "Flora of China", most in the south east. This is one of the most significant areas for the study of Chinese and Himalayan Species. This paper is a report of a trip to Linzhi Prefecture in S E Tibet, and discussion of some taxonomic anomalies.

Key words: Tibet; Linzhi prefecture; Doxiong La; *Rhodendrons*

中国知网 https://www.cnki.net

耿玉英《西藏林芝地区杜鹃花属植物资源考察及分类学考证》彩图

（正文见 36–38 页）

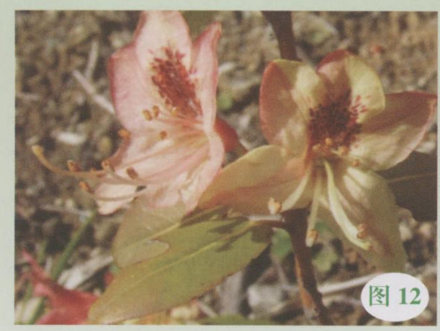
图 12

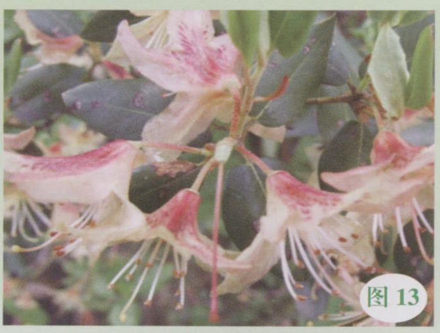
图 13

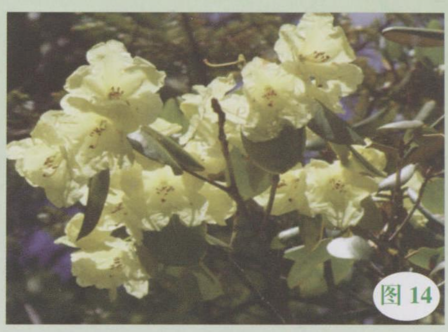
图 14

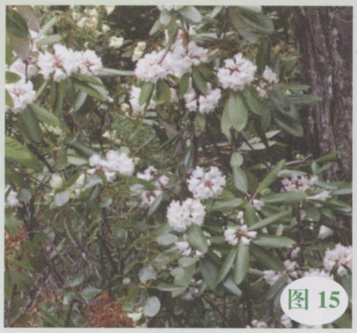
图 15

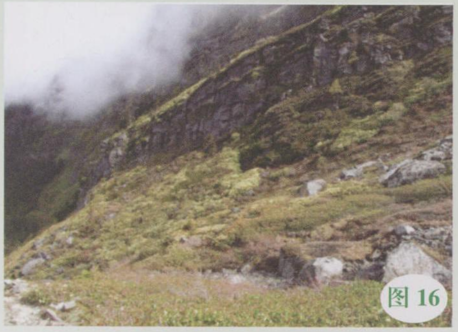
图 16

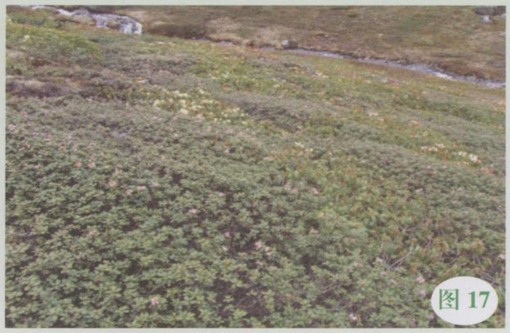
图 17

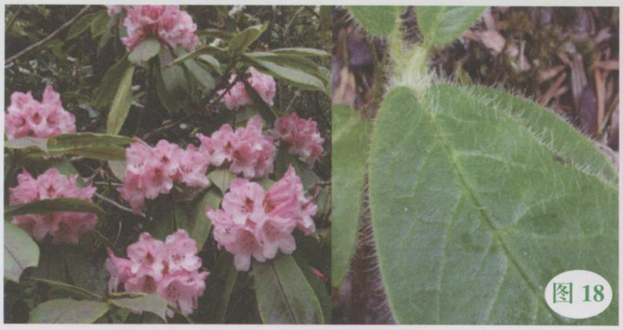
图 18

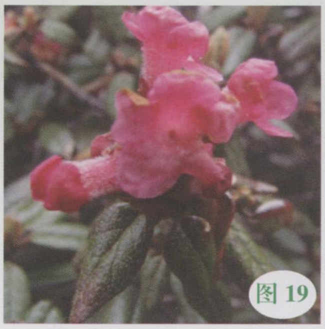
图 19

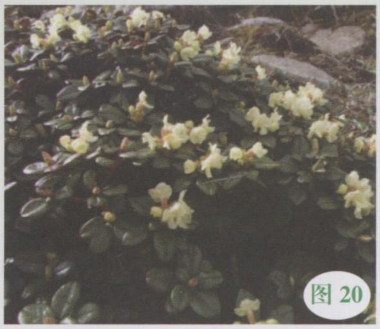
图 20

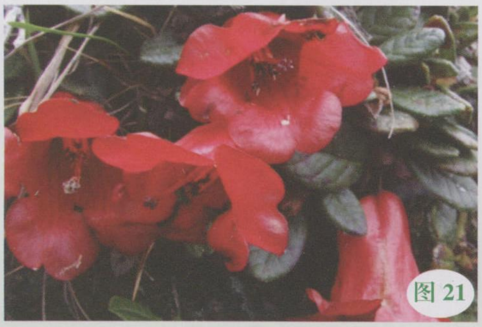
图 21

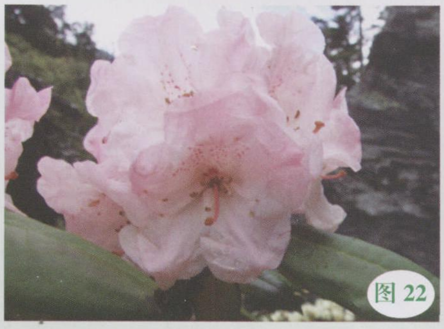
图 22

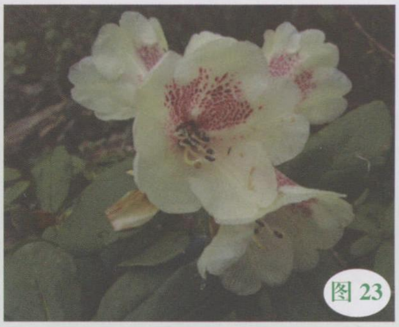
图 23

图 12：三花杜鹃，示花淡黄色带粉红色和淡粉红色带淡黄色，米林派镇；

图 13：三花杜鹃，示花序 4~5 或多花，林芝比日神山；

图 14：黄杯杜鹃，林芝色季拉山东坡；图 15：紫玉盘变种，米林派镇；

图 16：多雄拉北坡的杜鹃花之一；图 17：多雄拉北坡的杜鹃花之二；

图 18：粘毛杜鹃，多雄拉南坡（墨脱那格）；图 19：毛冠杜鹃，示花冠管外密被白色柔毛，多雄拉北坡；

图 20：髯花杜鹃，多雄拉北坡；图 21：紫背杜鹃，多雄拉北坡；

图 22：淡钟杜鹃，多雄拉南坡；图 23：盘萼杜鹃，示花冠颜色及斑点的变化，多雄拉北坡；

中国知网 https://www.cnki.net

耿玉英《西藏林芝地区杜鹃花属植物资源考察及分类学考证》彩图

（正文见 36–38 页）

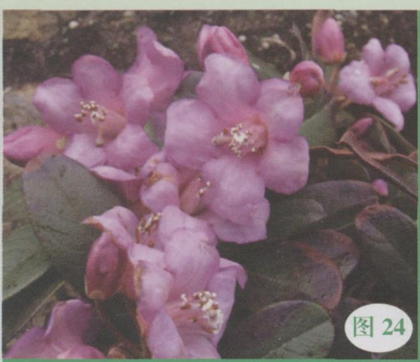
图 24

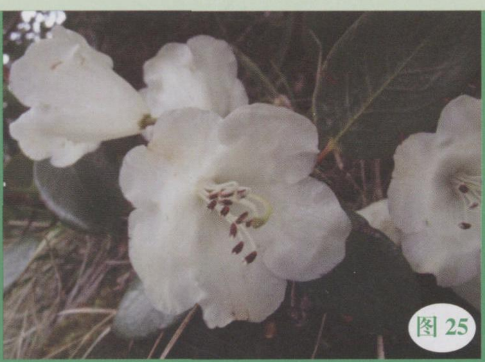
图 25

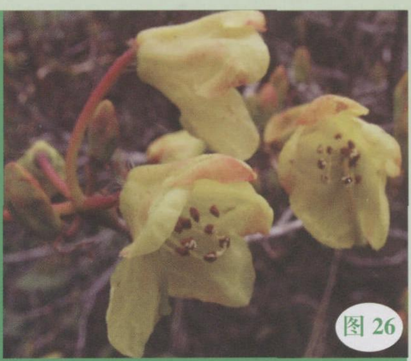
图 26

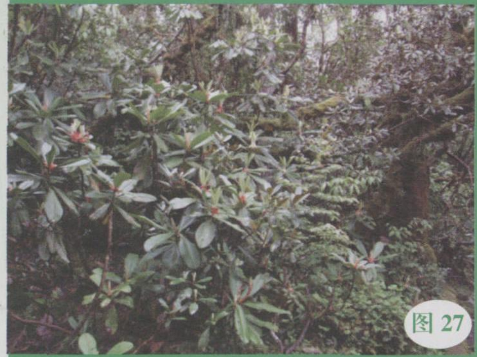
图 27

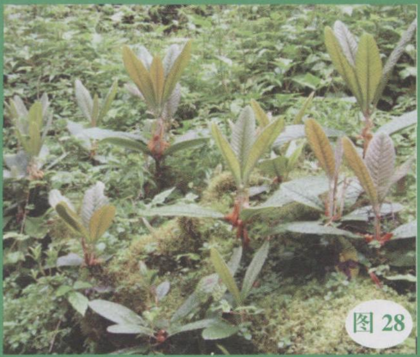
图 28

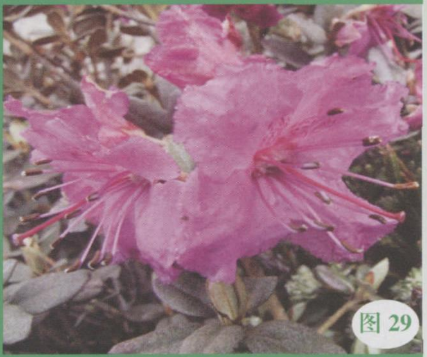
图 29

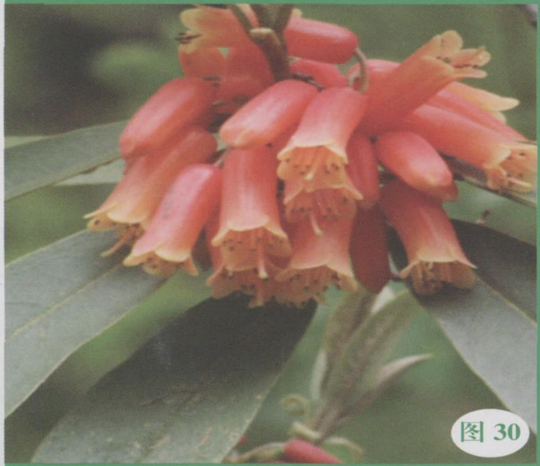
图 30

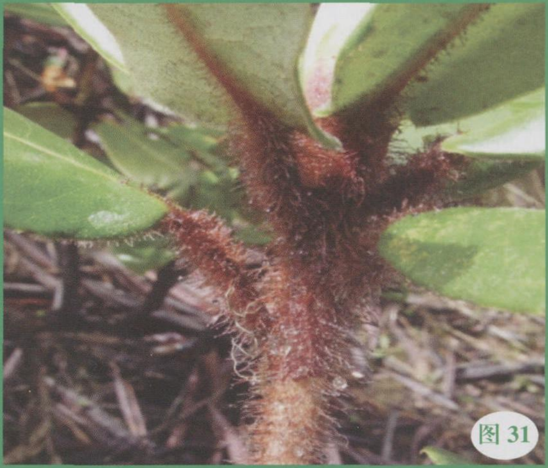
图 31

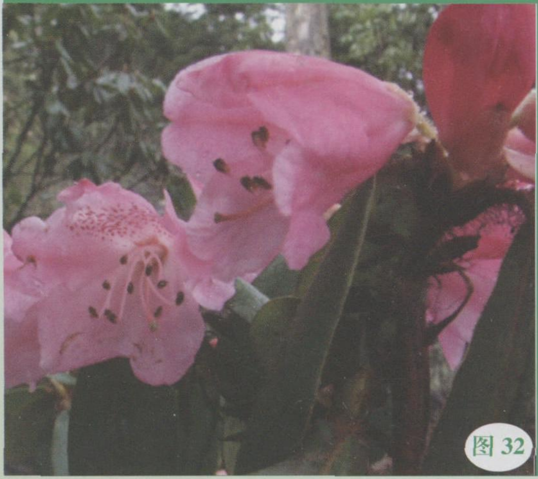
图 32

图 24：藏布雅容杜鹃，多雄拉南坡；

图 25：弯果杜鹃，示罕见的白色花，多雄拉北披；

图 26：弯月杜鹃，多雄拉南坡；

图 27：裸子植物林下的大叶杜鹃，多雄拉南坡（墨脱那格）；

图 28：凸尖杜鹃，示小苗，多雄拉南坡（墨脱那格）；

图 29：美被杜鹃，多雄拉北坡；

图 30：管花杜鹃，多雄拉南坡（墨脱那格）；

图 31：硬毛杜鹃，自林芝色季拉山西坡示，小枝和叶柄及叶下表面中脉密被腺状刚毛；

图 32：硬毛杜鹃，自林芝色季拉山西坡，示花。

中国知网 https://www.cnki.net

耿玉英《西藏林芝地区杜鹃花属植物资源考察及分类学考证》彩图

（正文见 36–38 页）

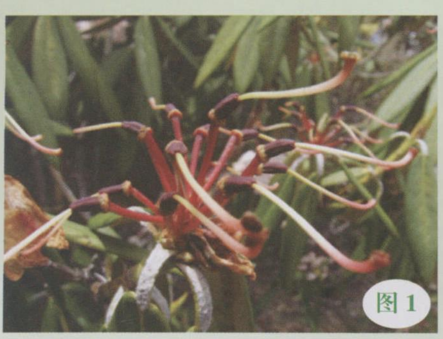
图 1

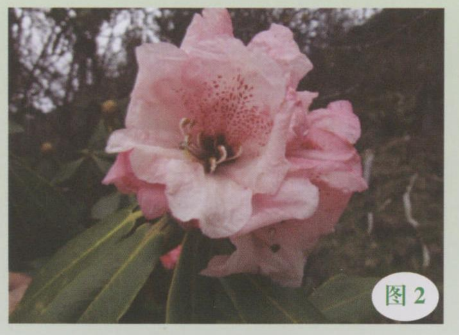
图 2

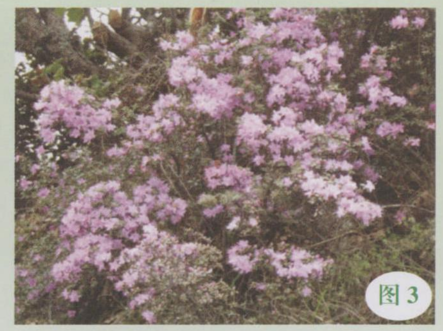
图 3

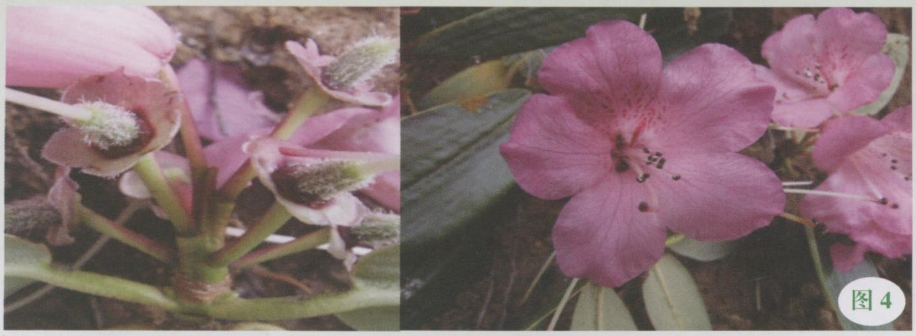
图 4

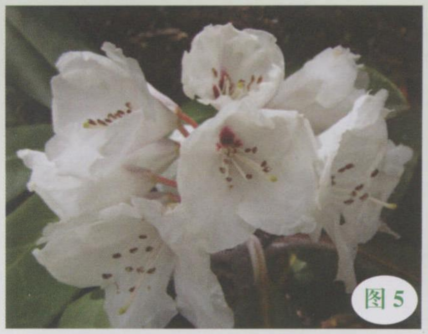
图 5

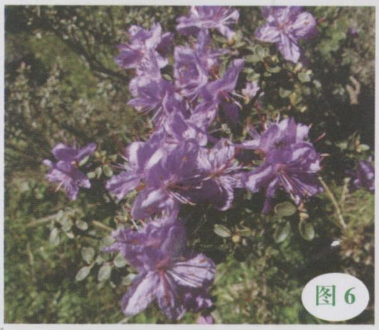
图 6

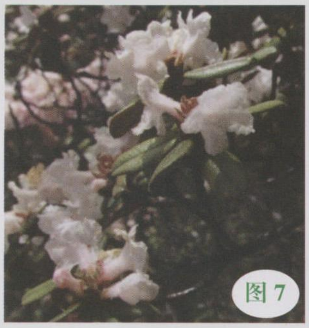
图 7

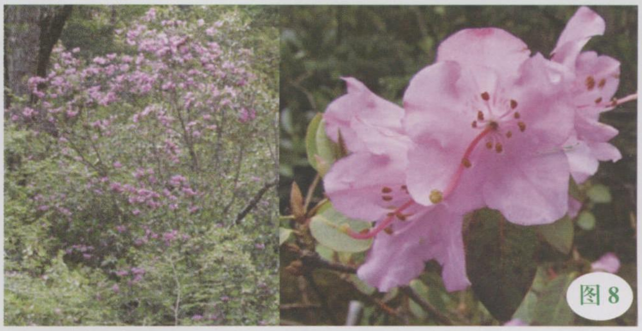
图 8

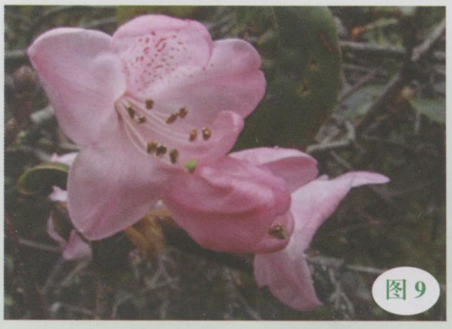
图 9

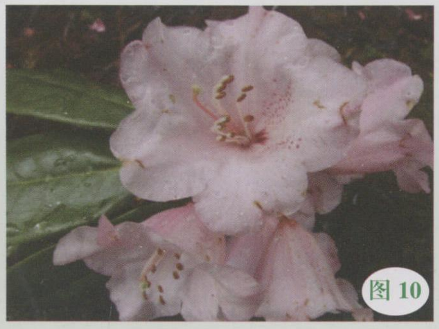
图 10

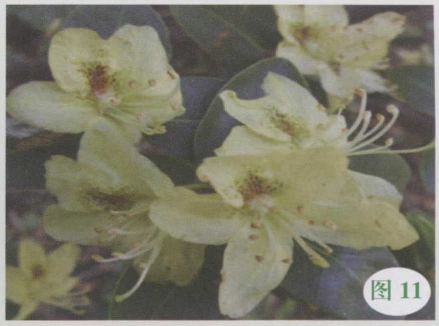
图 11

图 1：雪山杜鹃，示子房和花柱无毛，林芝色季拉山东坡；
图 2：裂毛雪山杜鹃，林芝色季拉山东坡；图 3：蜿蜒杜鹃，林芝比日神山；
图 4：猴斑杜鹃，示花萼形状和子房密被腺状毛（左）及花冠（右），多雄拉南坡（墨脱那格）；
图 5：鲁浪杜鹃，林芝色季拉山东坡；图 6：雪层杜鹃，色季拉山东坡；
图 7：林芝杜鹃，色季拉山东坡；图 8：山育杜鹃，林芝色季拉山东坡；
图 9：多变杜鹃，林芝色季拉山西坡；图 10：林芝光柱杜鹃，林芝色季拉山东坡；
图 11：三花杜鹃，示花淡黄色，林芝比日神山；

中国知网 https://www.cnki.net
