# Supplementary material for: Metabolomics Based on UPLC-MS/MS Revealed the Metabolic Differences Among Four Species of Rhododendrons in Linzhi, Xizang
Source: Metabolites. 2026 Mar 30;16(4):226. doi: 10.3390/metabo16040226 (PMC13117825; doi:10.3390/metabo16040226)
Supplement: Supplementary file 1 [file metabolites-16-00226-s001.zip › Supplementary documents/Supplementary References/3.Liu X, Zhou H, Yuan Y, et al (2021) Resources of Rhododendron in Xizang and their application prospects..pdf]

doi: 10.3969/j. issn. 1006 - 9690. 2021. 06. 016

西藏杜鹃花属植物资源及其应用前景分析

刘秀群[1,2*], 周浩洋[2], 袁一波[2], 傅 强[2], 邓 岚[2], 丰 明[3]

(1. 西藏自治区农牧科学院, 西藏 拉萨 850002; 2. 华中农业大学 园艺植物生物学教育部重点实验室/园艺林学学院, 湖北 武汉 430070; 3. 西藏自治区农牧科学院蔬菜研究所, 西藏 拉萨 850032)

摘 要 采用实地调查与文献查阅相结合的方法,对西藏杜鹃花属植物资源进行了统计和分析。西藏杜鹃花属植物共有180种(不包括种下分类等级),隶属于常绿杜鹃亚属、杜鹃亚属、马银花亚属、毛枝杜鹃亚属和糙叶杜鹃亚属5个亚属,以常绿杜鹃亚属(58.89%)和杜鹃亚属(38.33%)为主。从生活型来看,西藏杜鹃花属植物可分为灌木型(123种),乔木型(12种),灌木和乔木兼有型(45种)。从垂直分布来看,西藏杜鹃花属植物在海拔1000~5800 m的范围内均有分布,在海拔3000~4000 m范围内分布的种类最多(143种)。从水平分布来看,西藏除阿里地区之外的其他6市34县(区)均有分布,主要集中在西藏东南部和南部。还从树型、花色、花型、花期等方面分析了西藏杜鹃花属植物的观赏价值和应用潜力,并讨论了其在稳定整个西藏高寒地区脆弱生态方面的重要价值。

关键词 西藏; 杜鹃花属; 植物资源; 观赏价值; 生态价值

中图分类号: S326 **文献标识码**: A **文章编号**: 1006 - 9690(2021) 06 - 0089 - 06

Plant Resources and Application Prospects of *Rhododendron* in Tibet

Liu Xiuqun[1,2*], Zhou Haoyang[2], Yuan Yibo[2], Fu Qiang[2], Deng Lan[1], Feng Ming[3]

(1. Tibet Academy of Agricultural and Animal Husbandry Sciences, Lhasa 850002, China; 2. Key Lab. of Horticultural Plant Biology (Ministry of Education), College of Horticulture and Forestry Science, Huazhong Agricultural University, Wuhan 430070, China; 3. Institute of Vegetables, Tibet Academy of Agricultural and Animal Husbandry Sciences, Lhasa 850032, China)

Abstract Using the combination of field investigation and literature review, this study sorted out and analyzed the resources of *Rhododendron* in Tibet. There were 180 species (excluded subspecies and variants) in Tibet belonged to 5 subgenera (Subgen. *Hymenanthes*, Subgen. *Rhododendron*, Subgen. *Azaleastrum*, Subgen. *Pseudazalea* and Subgen. *Pseudorhodorastrum*). Among them, Subgen. *Hymenanthes* (58.89% in total species) and Subgen. *Rhododendron* (38.33% in total species) included most species. According to the life form, Tibetan *Rhododendron* included shrub form (123 species), tree form (12 species), shrub - tree form (45 species). Based on vertical distribution, Tibetan *Rhododendron* were distributed the areas of altitude span from 1000 to 5800m, mainly from 3000 - 4000 m (143 species). Based on the horizontal distribution, Tibetan *Rhododendron* were distributed in 6 cites 34 counties (districts) (no distribution in Ali area), mainly in Southeastern and Southern Tibet. This study analyzed the ornamental values and application prospects of Tibetan *Rhododendron* from plant shapes, flower colors, flower shapes and blossom periods, and also discussed the important value in stabilizing the fragile ecology in high - cold area in Tibet.

Key words *Rhododendron*; Plant resources; Ornamental value; Ecological value

杜鹃花是杜鹃花科(Ericaceae Juss.)杜鹃花属(*Rhododendron* L.)全部物种的统称。杜鹃花属在我国西南山地常形成大面积的天然群落,对保持水土、调节山区环境发挥重要的生态作用[1]。该属很多杜

收稿日期: 2021 - 02 - 24, 录用日期: 2021 - 06 - 10
基金项目: 西藏自治区重点研发及转化项目(XZ202001ZY0016N)。
作者简介: 刘秀群(1975 -), 男, 副教授, 研究方向为观赏植物种质资源。E - mail: liu_xiuqun@sina.com

(C)1994-2022 China Academic Journal Electronic Publishing House. All rights reserved. http://www.cnki.net

鹃花种类以及商业品种都为著名花卉，具有很高的观赏价值。其中，杜鹃花（*Rhododendron simsii* Planchon，俗称映山红）既是世界名花，也是中国十大传统名花之一。据资料记载全世界分布有杜鹃花属植物约960～1000种（不包括种下分类等级，下同），大多具有优良的观赏性状，被引种栽培的杜鹃花已超过600种；根据《中国植物志》（第57卷）和《Flora of China》（第14卷）以及其他资料记载中国杜鹃花属植物共有571种，其中409种为中国特有种；分布种类最多的是地处中国青藏高原和横断地区的西藏、四川和云南，有410种[2-3]。

国内外学者对杜鹃花属植物资源开展了一系列研究工作，内容包括资源调查，亲缘关系建立，基于形态形态学的分类研究，观赏价值评价，多样性与分布评估，遗传多样性与遗传分化研究，自然杂交分析，DNA条形码构建，种间杂交育种，RAD、转录组与全基因组测序等；涉及到的分布地点包括云南、四川、广西、辽宁、湖南、湖北、陕西、海南、山西、青海、甘肃等省（区），对于杜鹃花属植物主要分布地之一的西藏涉及较少。目前，对西藏杜鹃花属植物的研究主要有《西藏植物志》、《中国植物志》等资料对西藏杜鹃花属植物的描述，以及对色季拉山杜鹃花植物资源种类[4-6]、亲缘关系[7]、群落学特征[8-9]、遗传多样性[10]、园林应用[11]、环境适应性[12]等研究。

西藏地处高原地带，具有高辐射、严寒和低氧等环境特征，独特的地理位置和气候条件孕育了丰富的植物区系，为杜鹃花属植物生长提供了最佳生境，与云南、四川共同形成世界杜鹃花的分布中心。虽然目前对西藏杜鹃花属植物的研究相对较少，但是其生态作用依然重要，其观赏价值依然显著，应用前景依然可观。因此，开展西藏野生杜鹃花属植物资源种类的调查、整理和分析，为在科学保护的基础上的合理开发利用奠定基础。

1　研究区概况

西藏自治区位于青藏高原西南部，地处北纬26°50′～36°53′，东经78°25′～99°06′，面积为120×10^4 km^2。西藏东南部是高山峡谷，属热带、亚热带气候，并受印度洋暖湿气流影响，雨量充沛，从而形成了高差悬殊而又各有特色的植物垂直带谱；北部和西北部地势高，湖泊众多，平均海拔4000 m以上，雨量稀少，气候寒冷干旱[13]。西藏高原地形复杂、气候独特，有世界上最齐全的生物气候带，植被种类丰富，类型多样，区系成分复杂[14]，被称为“地球第三极”，是世界生物多样性热点地区之一。据《西藏植物志》记载，西藏全区维管束植物共有208科1258属5766种；据2013年完成的《Flora of China》记载，西藏有182科1294属7100种维管束植物（不包括蕨类植物）[13]。西藏野生花卉种类众多，约有1000多种，资源丰富，以高山花卉最具特色，其中杜鹃花属（*Rhododendron*）、报春花属（*Primula* L.）、龙胆属（*Gentiana*（Tourn.）L.）、绿绒蒿属（*Meconpsis* Vig.）等都是世界著名的高山花卉。另外，蔷薇属（*Rosa* L.）、杏属（*Armeniaca* Mill.）、芍药属（*Paeonia* L.）、马先蒿属（*Pedicularis* L.）、各种兰科植物等著名花卉在西藏都有丰富的资源分布。

2　研究方法

本次研究采取线路调查法并结合访问当地居民的形式进行。调查工作主要分4次进行，分别于2019年5～6月、2019年7～8月、2020年7～8月、2020年9月调查了西藏自治区6市43县（区）（阿里地区无杜鹃花属植物分布记载，没有调查），其中包括拉萨市8县（区），和林芝市7县（区），山南市8县（区），那曲市4县（区），昌都市5县（区），日喀则市11县（区）。在调查线路上进行标本采集、整理、鉴定，并以《中国植物志》及其英文版《Flora of China》、《西藏植物志》等资料对杜鹃花属植物的描述为主要依据，结合已经发表的关于西藏杜鹃花属植物的相关研究[4-10]，统计、整理和分析西藏野生杜鹃花属植物资源的种类、形态指标、生态环境、观赏特性、园林用途等。

3　结果与分析

3.1　西藏杜鹃花属植物种类组成及分布

3.1.1　种类组成

通过调查并结合前述相关资料研究，结果表明西藏杜鹃花属植物隶属于常绿杜鹃亚属（Subgen. *Hymenanthes*）、杜鹃亚属（Subgen. *Rhododendron*）、毛枝杜鹃亚属（Subgen. *Pseudazalea*）、马银花亚属（Subgen. *Azaleastrum*）和糙叶杜鹃亚属（Subgen. *Pseudorhodorastrum*）5亚属，共180种14亚种45变种。本研究分析了各亚属的种类数量（不包括种下分类等级，下同），及其占西藏杜鹃花属总种数的百分比（见表1）。常绿杜鹃亚属中主要以常绿杜鹃组（Sect. Ponticum）的火红杜鹃亚组（Subsect. Neriiflora）、大理杜鹃亚组（Subsect. Taliensia）、蜜腺杜鹃亚组（Subsect. Thomsonia）、漏斗杜鹃亚组（Subsect.

(C)1994-2022 China Academic Journal Electronic Publishing House. All rights reserved.　http://www.cnki.net

Selensia) 等为主,其中火红杜鹃亚组,有 19 种,占该亚属的 17.92%。杜鹃亚属中主要以髯花杜鹃组(Sect. Pogonanthum) 及杜鹃组(Sect. Rhododendron) 的有鳞大花亚组(Subsect. Maddenia)、黄花杜鹃亚组(Subsect. Boothia)、高山杜鹃亚组(Subsect. Lapponica) 为主;其中,有鳞大花亚组有 13 种,占该亚属的 18.84%。毛枝杜鹃亚属种类较少,下不分组。马银花亚属仅有马银花组(Sect. Azaleastrum) 的墨脱马银花(*Rh. medoense*) 1 种。糙叶杜鹃亚属仅有帚枝杜鹃组(Sect. Rhabdorhodion) 的柳条杜鹃(*Rh. virgatum*) 1 种。

3.1.2　生活型

从生活型来看,西藏杜鹃花属植物可分为灌木型、乔木型、灌木和乔木兼有型 3 类,并分析了它们的种类构成和占西藏杜鹃花属总种数的百分比(见表 2)。西藏杜鹃花属植物种类以灌木型为主,灌木、乔木兼有型次之,乔木型最少。

表 1　西藏杜鹃花属植物种类组成

Table 1　Species composition of *Rhododendron* in Tibet

亚属	种类数量	占西藏杜鹃花属总种数百分比(%)
常绿杜鹃亚属	106 种 9 亚种 34 变种	58.89
杜鹃亚属	69 种 5 亚种 7 变种	38.33
毛枝杜鹃亚属	3 种 4 变种	1.67
马银花亚属	1 种	0.56
糙叶杜鹃亚属	1 种	0.56

表 2　西藏杜鹃花属植物生活型

Table 2　Life forms of *Rhododendron* in Tibet

生活型	种类数量	占西藏杜鹃花属总种数百分比(%)
灌木型	123 种 10 亚种 39 变种	68.33
乔木型	12 种 2 变种	6.67
灌木和乔木兼有型	45 种 4 亚种 4 变种	25

3.1.3　垂直分布

从垂直分布来看,西藏杜鹃花属植物在海拔 1 000 ~ 5 800 m 的范围内均有分布,例如分布于墨脱和察隅等地的大白杜鹃(*Rh. decorum* Franch.) 的下线可以达到海拔 1 000 m 左右,上线可以达到 4 000 m 左右;而分布于洛扎、定日、吉隆、聂拉木等地的毛花杜鹃(*Rh. hypenanthum* Balf. F.),其分布的海拔下线在 3 500 m 左右,而海拔上线可以达到 5 200 m 左右;而橙黄杜鹃(*Rh. Citriniflorum* Balf. f. et Forrest var. *citriniflorum*) 的分布上线还可以达到 5 400 m;在西藏广泛分布的雪层杜鹃(*Rh. nivale* Hook. f. subsp. *nivale*) 的分布海拔上线甚至可以达到 5 800 m。本研究按照海拔梯度分析了西藏杜鹃花属植物的垂直分布情况,并列举了不同海拔梯度的代表种类(同一物种在不同海拔之间有分布跨度,种类计算有重复)(见表 3)。西藏杜鹃花属植物在海拔 3 000 ~ 4 000 m 范围内分布的种类数量最多;其次是海拔 2 000 ~ 3 000 m 范围内分布的种类数量较多;海拔 4 000 ~ 5 000 m 范围内分布种类数量为第三;海拔 1 000 ~ 2 000 m 范围内分布种类数量为第四;海拔5 000 m 以上范围内分布的种类数量最少,涉及种类的主要分布区都在海拔 3 000 ~ 5 000 m 以内,但是其分布的海拔上线可以延伸到 5 000 m 以上。

表 3　西藏杜鹃花属植物垂直分布

Table 3　Vertical distributions of *Rhododendron* in Tibet

海拔范围	种类数量	代表种类
>5 000 m	6 种<br>1 亚种	弯柱杜鹃、毛花杜鹃、橙黄杜鹃、雪层杜鹃、草莓花杜鹃、毛花杜鹃、毛冠杜鹃等
4 000 ~ 5 000 m	45 种<br>21 变种	宽钟杜鹃、毛冠杜鹃、宽筒杜鹃、米林杜鹃、簇毛杜鹃、辐花杜鹃、矮小杜鹃、滇藏杜鹃、毛嘴杜鹃、栎叶杜鹃、多叶杜鹃、短蕊杜鹃、广口杜鹃、砾石杜鹃、匍匐杜鹃、宏钟杜鹃、橙黄杜鹃、杯萼杜鹃、单色杜鹃、美被杜鹃等
3 000 ~ 4 000 m	143 种<br>31 变种	棕背川滇杜鹃、云南杜鹃、白背杜鹃、凸尖杜鹃、山育杜鹃、林生杜鹃、铜色杜鹃、睫毛杜鹃、毛枝杜鹃、团花杜鹃、一朵花杜鹃、糙毛杜鹃、独龙杜鹃、怒江杜鹃、弯果杜鹃、杂色杜鹃、紫背杜鹃、多趣杜鹃、半圆叶杜鹃、黄杯杜鹃等
2 000 ~ 3 000 m	88 种<br>7 变种	木兰杜鹃、瘤枝杜鹃、瓦弄杜鹃、蜿蜒杜鹃、革叶杜鹃、藏东杜鹃、雪山杜鹃、卷叶杜鹃、亮叶杜鹃、硫磺杜鹃、藏布杜鹃、招展杜鹃、红棕杜鹃、镰果杜鹃等
1 000 ~ 2 000 m	22 种<br>1 变种	大白杜鹃、树形杜鹃、鲜黄杜鹃、巨魁杜鹃、墨脱马银花、强壮杜鹃、越橘杜鹃等

3.1.4　水平分布

从水平分布来看,西藏除阿里地区无杜鹃花属植物分布外,其他 6 个地级市均有分布。180 种杜鹃花属植物遍布西藏 34 个县(区),县(区)级覆盖率达到45.83%,主要集中在西藏东南部、南部、东部及东北部。林芝市分布最多最广,察隅县有 72 种,墨脱县有63 种,巴宜区有38 种,米林县有 32 种,波密县有30 种,朗县有 12 种,工布江达县有 5 种(各县之间有重复种)。其次是山南市和日喀则市,分别有杜鹃花属植物39 种和31 种。另外 3 个地级市则零散分布有少量杜鹃花属植物。据统计,西藏杜鹃花属植物中雪层杜鹃(*Rh. nivale* Hook. f.) 最为常见,广泛分布于西藏 26 个县(区);其次是鳞腺杜鹃(*Rh. lepidotum* Wall. ex G. Don)、藏南杜鹃(*Rh. principis* Bur. et Franch.)、白毛杜鹃(*Rh. vellereum*

(C)1994-2022 China Academic Journal Electronic Publishing House. All rights reserved.　http://www.cnki.net

Hutch. et Tagg) 和毛喉杜鹃(*Rh. cephalanthum* Franch.),分布范围均在10个以上县(区);髯花杜鹃(*Rh. anthopogon* D. Don)、钟花杜鹃(*Rh. campanulatum* D. Don subsp. *Campanulatum*)、三花杜鹃(*Rh. triflorum* Hook. f.)、树形杜鹃(*Rh. arboreum* Smith var. *arboreum*)等稍常见,分布于7~8个县(区)间;而折萼杜鹃(*Rh. auritum* Tagg)、黄药杜鹃(*Rh. flavantherum* Hutch. et K. Ward)、石峰杜鹃(*Rh. scopulorum* Hutch.)等则很少见,仅在个别地点有分布。

3.2 西藏杜鹃花属植物的应用价值

3.2.1 观赏价值

树型多样。杜鹃花属植物的树型多样,在木本花卉中很难再找出一类花卉其树型的多变程度能与杜鹃花属相媲美。西藏杜鹃花属植物的树型从几厘米高的垫状灌木到十几米甚至二十几米高的高大乔木应有尽有,并且一种杜鹃花还可能有几种不同的树型。其中,较大的常绿乔木,如强壮杜鹃(*Rh. magnificum* K. Ward)、凸尖杜鹃(*Rh. sinogrande* Balf. F. et W. W. Smith)、羞怯杜鹃(*Rh. pudorosum* Cowan)、墨脱杜鹃(*Rh. montroseanum* Davidian)、巨魁杜鹃(*R. grande* Wight)等均可长至高10 m以上,强壮杜鹃甚至可高达18 m;一般的常绿乔木,如树形杜鹃、藏东杜鹃(*Rh. oreogenum* L. C. Hu)、翘首杜鹃(*Rh. protistum* Balf. F. et Forrest)、大王杜鹃(*Rh. rex* Levl.)、白毛杜鹃(*Rh. vellereum* Hutch. et Tagg)等植株高度一般在3~10m;灌木类的有腺房杜鹃(*Rh. adenogynum* Diels)、雪山杜鹃(*Rh. aganniphum* Balf. f. et K. Ward var. *aganniphum*)、细枝杜鹃(*Rh. amandum* Cowan)、变光杜鹃(*Rh. calvescens* Balf. F. et Forrest)、隆子杜鹃(*Rh. dekatanum* Cowan)、焰红杜鹃(*Rh. miniatum* Cowan)等,这些植株高度较低,基本在1~7 m;而一些小灌木,如髯花杜鹃(*Rh. anthopogon* D. Don)、黄花花杜鹃(*Rh. boothii* Nutt.)、弯柱杜鹃(*Rh. campylogynum* Franch.)、泡泡叶杜鹃(*Rh. edgeworthii* Hook. f.)、匍匐杜鹃(*Rh. erastum* Balf. F. et Forrest)、草莓花杜鹃(*Rh. fragariiflorum* Kingdon-Ward)等大多不超过1 m,呈平铺、垫状或附生,尤其是草莓花杜鹃,其植株高度可低至仅5 cm;另外还有一些种的杜鹃花兼有灌木和乔木的树形,如团花杜鹃(*Rh. anthosphaerum* Diels.)、宽钟杜鹃(*Rh. beesianum* Diels.)、三花杜鹃、瓦弄杜鹃(*Rh. walongense* K. Ward)等。

花色丰富。花色是对杜鹃花属植物观赏价值举足轻重的重要观赏性状之一。西藏野生杜鹃花属植物的花色包括红色系、白色系、黄色系、紫色系、紫红色系、绿黄色系等,其中以红色系、白色系和黄色系居多。本研究分析了各种色系的种类数量,并列举了部分代表种类(由于有些种具有多种色系,故不同色系的种类有重复)(见表4)。黄色系、紫色系、绿黄色系的种类在品种培育中具有明显资源优势,此类杜鹃花在市面上流通较少,为杜鹃花花色的选育提供了丰富的原材料。

表4 西藏杜鹃花属植物的花色多样性

Table 4 Diversity of flower colors of *Rhododendron* in Tibet

色系类型	种类数量	代表种类
红色系	125种32变种11亚种	硬刺杜鹃、变光杜鹃、樱花杜鹃等
白色系	74种14变种5亚种	雪山杜鹃、夺目杜鹃、革叶杜鹃等
黄色系	47种12变种	细枝杜鹃、黄花花杜鹃、弯果杜鹃等
紫色系	23种5亚种3变种	铜叶钟花杜鹃、草莓花杜鹃、短蕊杜鹃等
紫红色系	14种1变种	弯柱杜鹃、灰白杜鹃、矮小杜鹃等
绿黄色系	6种2变种	弯月杜鹃、异鳞杜鹃、显绿杜鹃等

花型优美。杜鹃花属植物的花冠通常为辐射对称或中轴对称,其轮廓、大小、空间结构的不同构成了其多样、优美的姿态。西藏杜鹃花属植物根据其整体形状可分为杯状、漏斗状、辐状、盘状、管状、钟形、喇叭状、筒状,细分则可进一步分为漏斗状钟形、管状钟形、宽漏斗状、狭筒状、宽钟状、斜钟形等,形态各异,观赏价值极高。

花期较长。西藏杜鹃花属植物花期跨度较长,不同种类在2~9月的时间范围内均有花盛开,例如睫毛杜鹃(*Rh. ciliatum* Hook. f.)的花期可从2月持续到5月;红棕杜鹃(*Rh. rubiginosum* Franch.)在3~6月都有花开放;雪层杜鹃、林芝杜鹃(*Rh. nyingchiense* R. C. Fang et S. H. Huang)的花期从5月一直开到8月,横跨整个夏季。但大部分西藏杜鹃花属植物的花期还是集中在5~6月,在此期间有花盛开的有145种41变种12亚种;在2~4月有花的有52种4变种1亚种,如睫毛杜鹃、强壮杜鹃等;7~8月开花的有44种22变种4亚种,如亮鳞杜鹃(*Rh. heliolepis* Franch var. *heliolepis*)、雪层杜鹃、薄叶朱砂杜鹃(*Rh. tenuifolium* R. C. Fang et S. H. Huang)等;而独龙杜鹃(*Rh. keleticum* Balf. f. et Forrest)和一朵花杜鹃(*Rh. monanthum* Balf. F. et W.

(C)1994-2022 China Academic Journal Electronic Publishing House. All rights reserved. http://www.cnki.net

W. Smith）更是在 9 月份仍在开花。

3.2.2　生态价值

杜鹃花属植物中有一些种类是乔木或者小乔木，但大多数种类是灌木，其植株一般无明显主干，高度在 5 m 以下，常形成杜鹃花灌丛，作为所在地的植被主体，成为优势种或建群种。这些杜鹃花灌丛不仅是亚热带地区的典型灌丛群落[15]，也广泛分布于我国的高寒山区，是高寒山区生态系统的建群种[16]。特别是在西藏，由于高寒山区生态条件严酷，除杜鹃花灌丛在林线附近成片分布外，其他植物生长困难，因此杜鹃花属植物在西藏高寒山区生态系统中占据着重要的地位和作用[8,17-18]。生长在西藏高寒地带的杜鹃花属植物耐寒性强，通常植株矮小，枝繁叶茂，根系发达，常成丛长成密不可入的灌木林。这对保水固土十分有利，具有重要的生态价值[19]，已经成为中国西南与西北部内外陆流域的水源涵养林之一，其稳定存在对于青藏高原和相邻干旱地区的水热平衡具有重要的生态学意义[20-21]。西藏高寒杜鹃花灌丛群落也为众多藏药材资源提供了良好的天然生长环境。在西藏色季拉山的杜鹃花灌丛群落下，就生长有长鞭红景天、柴胡红景天、塔黄、矮棱子芹、岩白菜、肿柄雪莲、苞叶雪莲等珍贵的藏药材，为区域经济发展做出了重要贡献[8]。西藏杜鹃花灌丛所在的草地既是牦牛赖以生存的牧场，也是许多珍贵野生动物的栖居地，对稳定整个高寒山区脆弱草地生态具有重要意义[22-25]。

3.2.3　引种与驯化前景

直接引种驯化和利用分布于高寒地区的西藏杜鹃花属植物绝大多数还是存在较大困难，但是在西藏及其邻近地区进行利用应该难度不大。目前在美丽西藏的视角下，也需要应用大量的西藏本土的优良观赏植物资源为西藏及其邻近地区的园林绿化以及生态建设服务。在西藏杜鹃花属植物中，花色绚丽、多姿、独特的多种野生种如夺目杜鹃、蜿蜒杜鹃（*Rh. bulu* Hutch.）、藏布杜鹃（*Rh. charitopes* subsp. *tsangpoense*（K. Ward）Cullen）、隆子杜鹃、鳞腺杜鹃、异鳞杜鹃等可用作花篱。一些花期较长的种类，如睫毛杜鹃、雪层杜鹃、林芝杜鹃等可用作花坛、花镜，供人们长时间观赏。而强壮杜鹃、墨脱杜鹃、巨魁杜鹃等树形较高大的种类亦可作孤植树种用于庭院、广场、公园等地。

虽然从高寒地区的西藏直接引种杜鹃花属植物到低海拔应用存在困难，但是利用其在树型、花色、花型、花期、香味等方面的优良资源，与已经适应低海拔地区的杜鹃花属植物进行杂交育种，应该是杜鹃花植物育种的方向之一。利用西藏杜鹃花属植物的树型多样性，既可作高大树种选育的亲本，又可为盆栽杜鹃花的育种提供矮化基因。亮柠檬黄色的黄花杜鹃、鲜黄色的纯黄杜鹃（*Rh. chrysodoron* Tagg ex Hutch.）、深红紫色的灰白杜鹃、白色的大萼杜鹃（*Rh. megacalyx* Balf. F. et K. Ward）等色彩丰富的西藏杜鹃花为纯色系杜鹃花的选育奠定了坚实的基础。附生杜鹃（*Rh. dendricola* Hutch.）、泡泡叶杜鹃、隐脉杜鹃（*Rh. maddenii* Hook. f. subsp. *maddenii*）、石峰杜鹃、中国木兰杜鹃（*Rh. Sinonuttallii* Balf. F. et Forrest）、瓦弄杜鹃等具有香味的杜鹃花可通过各种育种手段为杜鹃花的香味育种提供重要资源。

4　展　望

西藏作为杜鹃花属植物的分布中心之一，虽然已经对其资源种类、亲缘关系、多样性、环境适应性等方面进行了一定研究，但是大多数研究主要集中在色季拉山等极少数地点。今后应该进一步加强对西藏高寒地区杜鹃花属植物种质资源调查、收集、评价，特别是对西藏杜鹃花野生资源集中连片分布区在生态保护和资源保护的基础上，进行适度开发、合理利用；深入开展西藏杜鹃花属植物在高寒山区的严酷生态条件下，仍能在林线附近成片分布的适应性机制；大力研究杜鹃花属植物在西藏作为重要的保水固土的水源涵养林功能，使其能够更加充分地发挥生态价值；加强有关杜鹃花作为我国宝贵的优势资源植物的认识，加强杜鹃花知识的传播，提升杜鹃花产业的研发与创新能力[26]。

参考文献：

[1]　周兰英，王永清，张丽．26 种杜鹃花属植物花粉形态及分类研究［J］．林业科学，2008，44(2)：55－63.

[2]　Chamberlain D F，Hyam R，Argent G，et al. The genus Rhododendron，its classification and synonymy［M］．Edinburgh：Royal Botanic Garden，1996.

[3]　耿玉英．中国杜鹃花属植物［M］．上海：上海科学技术出版社，2014：1－28.

[4]　郑维列，潘刚，徐阿生，等．西藏色季拉山杜鹃花种质资源的初步研究［J］．园艺学报，1995，22(2)：166－170.

[5]　耿玉英．藏林芝地区杜鹃花属植物资源考察及分类学考证［J］．中国园艺文摘，2010(11)：36－38.

[6]　包红光．西藏色季拉山杜鹃花属植物资源及在园林应用中的研究［J］．现代园艺，2013(2)：164－165.

[7]　徐静静，张良英，赵冰，等．色季拉山 8 个杜鹃花野生种亲缘关系的 ISSR 分析［J］．种子，2016，35(3)：1－4.

(C)1994-2022 China Academic Journal Electronic Publishing House. All rights reserved.　http://www.cnki.net

[8] 唐晓琴，卢杰，边巴多吉．西藏色季拉山高寒杜鹃群落学特征及其保护[J]．山地农业生物学报，2011，30(4)：294－299.
[9] 沈丽，石松林，李景吉，等．珠穆朗玛峰国家级自然保护区高山杜鹃群落多样性研究[J]．西北植物学报，2014，34(12)：2553－2561.
[10] 徐静静，赵冰，张良英，等．西藏地区雪层杜鹃遗传多样性的 AFLP 分析[J]．植物研究，2017，37(1)：88－95.
[11] 任军辉，张宇钰，丁云春，等．西藏色季拉山杜鹃花属植物资源及其园林应用[J]．陕西林业科技，2013 (6)：28－31.
[12] 郭文文，卓么草，方江平，等．藏东南色季拉山薄毛海绵杜鹃叶解剖结构特征与环境适应性[J]．西北植物学报，2020，40(5)：0811－0818.
[13] 马欣堂，杜玉芬，班勤，等．西藏野生花卉[M]．河南科技出版社，2016.
[14] 倪志诚．西藏植被及花卉[J]．西藏科技，1990 (4)：25－30.
[15] 方精云，郭兆迪，朴世龙，等．1981－2000 年中国陆地植被碳汇的估算[J]．中国科学 D 辑：地球科学，2007，37(6)：804－812.
[16] 吴征镒．西藏植物志(第 3 卷)卷[M]．北京：科学出版社，1986：552－732.
[17] 中国农业百科全书编辑部．中国农业百科全书·林业卷(上)[M]．北京：农业出版社，1989：126－127.
[18] 苏楷淇，陈雅琦，杨惠敏．杜鹃属植物与杜鹃灌丛群落的研究进展[J]．热带亚热带植物学报，2020，28(5)：527－536.
[19] 冯正波，庄平，张超，等．野生杜鹃花迁地保护适应性评价[J]．云南植物研究，2004，26(5)：497－506.
[22] 中国植被编辑委员会．中国植被[M]．北京：科学出版社，1980：430－432.
[21] 洛桑，灵智多杰．青藏高原环境与发展概论[M]．北京：中国藏学出版社，1996：74－77.
[22] 于应文，胡自治，张德罡，等．金露梅灌丛净第一性生产力[J]．草业学报，2000，9(4)：33－39.
[23] 张德罡．砍伐与滑坡对东祁连山杜鹃灌丛草地土壤肥力的影响[J]．草业学报，2002，11(3)：72－75.
[24] 张德罡，胡自治．东祁连山杜鹃灌丛草地灌木种群分布格局研究[J]．草地学报，2003，11(3)：234－239.
[25] 曹文侠，张德罡，洪绂曾．祁连山高寒灌丛草地杜鹃属植物的水分动态及生态适应[J]．草地学报，2006，14(1)：67－71.
[26] 吴荭，杨雪梅，邵慧敏，等．杜鹃花产业的种质资源基础：现状、问题与对策[J]．生物多样性，2013，21(5)：628－634.

(上接第 88 页)

总之，叶尔羌河流植物区系及资源多样性方面具有以下特点：该区域的植物种类较丰富，地理成分以北温带和地中海、西亚至中亚分布为主；药用植物资源多，所含药用植物、濒危和特有物种种类较多。因此，应该建立药用植物种子资源库，同时应该加强对濒危物种的保护；对该区域植物种质资源合理开发利用的同时提出相应的措施，保护物种的多样性。

参考文献:

[1] 吴征镒，孙航，周浙昆，等．中国种子植物区系地理[J]．生物多样性，2011，19(1)：148.
[2] 陈灵芝．中国植物区系与植被地理(第 3 版)[M]．北京：科学出版社，2014：52－54.
[3] 孙航，邓涛，陈永生，等．植物区系地理研究现状及发展趋势[J]．生物多样性，2017，25(2)：111－122.
[4] 吴征镒．种子植物分布区类型及其起源和分化(第 1 版)[M]．昆明：云南科技出版社，2006：38－40.
[5] 王荷生．植物区系地理[M]．北京：科学出版社，1992：1－155.
[6] 刘慎谔．中国南部及西南部植物地理概要[C]．刘慎谔文集．北京：科学出版社，1985：47－50.
[7] 刘慎谔．云南植物地理[C]．刘慎谔文集．北京：科学出版社，1985：86－111.
[8] 王彦涛．叶尔羌河流域植物区系及植被研究[D]．乌鲁木齐：新疆师范大学，2010.
[9] 吴征镒，周浙昆，李德铢，等．世界种子植物科的分布区类型系统[J]．云南植物研究，2003，25 (3)：245－257.
[10] 潘晓玲．新疆种子植物科的区系地理成分分析[J]．植物研究，1997，17 (4)：45－50.
[11] 郑度．喀喇昆仑山－昆仑山地区自然地理[M]．北京：科学出版社，1999：12－14.
[12] 潘晓玲．新疆种子植物属的区系地理成分分析[J]．植物研究，1999，19 (3)：3－5.
[13] 关克俭．中国植物志[M]．北京：科学出版社，1979－2004：28－30.
[14] 新疆植物志编辑委员会．新疆植物志：1－6 卷[M]．乌鲁木齐：新疆科技卫生出版社，1992－ 1996.
[15] 王文采，杨宗宗，等．新疆翠雀花属二新种[J]．植物研究，2020，40(6)：801－804.
[16] 覃海宁，赵莉娜，于胜祥，等．中国被子植物濒危等级的评估[J]．生物多样性，2017，25(7)：745－757.
[17] 袁敏敏，阎平，杜珍珠，等．新疆西昆仑山菊科菊苣族植物区系分析[J]．干旱区研究，2017，34(1)：67－71.
[18] 张明理．中国西北干旱区和中亚植物区系地理研究[J]．生物多样性，2017，25(2)：147－156.
[19] 王蕾，施诗，廖文波，等．井冈山地区珍稀濒危植物及其生存状况[J]．生物多样性，2013，21(2)：163－177.
[20] 杨淑萍，阎平，任姗姗，等．新疆北塔山地区药用植物资源及多样性分析[J]．植物科学学报，2016，34(3)：371－380.

(C)1994-2022 China Academic Journal Electronic Publishing House. All rights reserved. http://www.cnki.net
