# Supplementary material for: Metabolomics Based on UPLC-MS/MS Revealed the Metabolic Differences Among Four Species of Rhododendrons in Linzhi, Xizang
Source: Metabolites. 2026 Mar 30;16(4):226. doi: 10.3390/metabo16040226 (PMC13117825; doi:10.3390/metabo16040226)
Supplement: Supplementary file 1 [file metabolites-16-00226-s001.zip › Supplementary documents/Supplementary References/6.Wang S, Chang Z, Zhang Y, et al (2023) Community composition and diversity of endophytic fungi in four Rhododendron species from Xizang.pdf]

西藏4种杜鹃属植物内生真菌群落组成及多样性分析

王素娟[1],常子惠[2],张永娟[1],卫波宁[1],德 吉[1]*

(1.西藏大学理学院,西藏拉萨 850000;2.西藏自治区农牧科学院农业研究所,西藏拉萨 850000)

摘要 [目的]挖掘西藏杜鹃花科杜鹃属植物内生真菌资源,研究米林杜鹃(*Rhododendron mainlingense*)、髯花杜鹃(*R. anthopogon*)、雪层杜鹃(*R. nivale*)和雪山杜鹃(*R. aganniphum*)内生真菌的群落组成及多样性。[方法]采用组织分离法对上述4种杜鹃的根、茎、叶和花内生真菌进行分离,用ITS分子标记方法进行菌株鉴定。[结果]西藏4种杜鹃属植物720个组织块中分离得到336株内生真菌,这些真菌被鉴定为2门5纲10目16科21属55种。4种杜鹃属植物内生真菌多样性指数无显著差异。雪山杜鹃($H'=1.96$,$D=0.80$)和雪层杜鹃($H'=1.81$,$D=0.77$)内生真菌Shannon多样性指数(H')和辛普森指数(D)高于髯花杜鹃($H'=1.36$,$D=0.71$)和米林杜鹃($H'=1.17$,$D=0.65$)内生真菌多样性。根、茎、叶、花不同部位内生真菌多样性也无显著差异。不同部位的H'和D指数分别为根部$H'=2.63$、$D=0.86$,茎$H'=2.35$、$D=0.83$,叶$H'=2.26$、$D=0.80$,花$H'=2.23$、$D=0.83$。[结论]研究结果为挖掘杜鹃中内生真菌资源和药效物质筛选研究提供了基础数据。

关键词 杜鹃;内生真菌;群落组成;多样性
中图分类号 Q948.1 **文献标识码** A
文章编号 0517-6611(2023)11-0138-06
doi:10.3969/j.issn.0517-6611.2023.11.034
开放科学(资源服务)标识码(OSID):

Analysis on the Community Composition and Diversity of Endophytic Fungi in Four Species of *Rhododendron* in Tibet

WANG Su-juan[1], CHANG Zi-hui[2], ZHANG Yong-juan[1] et al (1. School of Science, Tibet University, Lhasa, Tibet 850000; 2. Agriculture Research Institute, Tibet Academy of Agriculture and Animal Husbandry Sciences, Lhasa, Tibet 850000)

Abstract [Objective] To explore the endophytic fungal resources of *Rhododendron* genus in the *Rhododendron* family in Tibet, and to study the community composition and diversity of endophytic fungi in *Rhododendron* mainlingense, *R. anthopogon*, *R. nivale* and *R. aganniphum*. [Method] The endophytic fungi from roots, stems, leaves and flowers of four species were isolated using tissue isolation method and identified by ITS sequencing. [Result] A total of 336 endophytic fungi were isolated from 720 tissues blocks of 4 species of *Rhododendron* plants in Tibet. These fungi were identified as 55 species belonging to 2 phyla, 5 classes, 10 orders, 16 families, 21 genera and 55 species. There was no significant difference in the diversity index of endophytic fungi among the four *Rhododendron* species. The diversity shannon index (H') and simpson index(D) of endophytic fungi in *R. aganniphum* ($H'=1.96$, $D=0.80$) and *R. nivale* ($H'=1.81$, $D=0.77$) were higher than in *R. anthopogon* ($H'=1.36$, $D=0.71$) and *R. mainlingense* ($H'=1.17$, $D=0.65$). There was no significant difference in the diversity of endophytic fungi in different parts of roots, stems, leaves and flowers. The H' index and D index in different tissues as follows: $H'=2.63$, $D=0.86$ in roots, $H'=2.35$, $D=0.83$ in stems, $H'=2.26$, $D=0.80$ in leaves and $H'=2.23$, $D=0.83$ in flowers. [Conclusion] The research results provide basic data for the exploration of endophytic fungal resources and screening of pharmacological substances in *Rhododendron*.

Key words *Rhododendron*; Endophytic fungi; Community composition; Diversity

杜鹃属(*Rhododendron* L.)是杜鹃花科(Ericaceae)植物,其中米林杜鹃(*Rhododendron mainlingense*)、髯花杜鹃(*R. anthopogon*)、雪层杜鹃(*R. nivale*)和雪山杜鹃(*R. aganniphum*)主要分布于海拔2 700~5 800 m的高山地区[1]。据文献记载[2-4],杜鹃根、茎、叶、花和果均可入药,根部具有祛风止痛、消肿散瘀、止咳等功效,可用于治疗跌打损伤、风寒湿痹、癣疮;叶具有解毒、清热和止血的功效;花具有除湿祛风、化瘀止痛的疗效;果实具有祛风镇痛、平喘止咳、化瘀消肿等功效。杜鹃可用于治疗炎症相关的疾病,如支气管炎、风湿病、关节炎等。

药用植物内生真菌是植物微生态系统中的重要组成部分,植物内生真菌是生活在植物细胞中或在其生活史某一时期生活在植物组织内,不引起植物发生明显病变的一类真菌[5-8]。有的内生真菌与植物生长密切相关,影响着植物种子的萌发,可促进植物生长和竞争力。有的内生真菌具有合成宿主次生代谢产物的能力[9-12],同时植物真菌群落与寄主之间的相互作用是植物适应环境的重要因素[13-14]。自短叶红豆杉内生真菌中得到紫杉醇[15]以来,挖掘、筛选其他植物内生真菌药效物质已成为重要的研究方向。已有报道西藏米拉山雪层杜鹃中分离得到了4属内生真菌,分别为曲霉属(*Aspergillus*)、拟隐壳孢属(*Cryptosporiopsis*)、镰刀菌属(*Fusarium*)、壳多胞菌属(*Stagonospora*)[16]。但是,杜鹃种间内生真菌的差异和不同部位的内生真菌差异目前鲜见报道。该研究通过ITS分子标记方法对采集于西藏地区的4种杜鹃属植物的内生真菌进行鉴定与分析,比较不同种杜鹃之间内生真菌多样性,为挖掘杜鹃植物内生真菌资源以及筛选药效物质提供前期数据。

1 材料与方法

1.1 杜鹃花属植物内生真菌的样品采集 于2021年6—8月在西藏不同地区采集杜鹃属全株新鲜植物,采样信息见表1。每个采样点选取新鲜健康植株6株,样品采集后放置于4 ℃冰箱,待处理。

1.2 杜鹃属植物内生真菌的分离纯化及保存 将采集的新鲜杜鹃植物的根、茎、叶、花用无菌水冲洗干净。用已灭菌的解剖刀将根、茎、叶和花所需部分切成小块,其中根部组织192块、叶部组织192块、茎部组织192块、花部组织144块。在无菌条件下将切好的植物材料置于75%乙醇浸泡2 min

基金项目 西藏自治区科技厅中央支持地方项目(XZ2020001YD0003C);2022年中央支持地方高校专项(00060949)。
作者简介 王素娟(1999—),女,甘肃舟曲人,硕士研究生,研究方向:青藏高原生物多样性与进化生态学。*通信作者,副教授,博士,从事青藏高原生物多样性与进化生态学研究。
收稿日期 2022-11-30

(C)1994-2023 China Academic Journal Electronic Publishing House. All rights reserved. http://www.cnki.net

后,用无菌水冲洗 3 次,再用 1%次氯酸钠溶液浸泡 3 min 后,无菌水冲洗 3 次,然后将植物横截面贴入 PDA 培养皿(已加 Amp 和 Kan 抗生素)中培养 3~7 d。每隔 1 d 观察,将植物组织块周围长出的不同菌落分离,接种到新的 PDA 固体培养基中,分离纯化 3 次,得到纯化的菌落。用组织块印迹法和无菌水检测法作为对照,来检验植株表面消毒情况。

表 1　样品分布与采集信息

Table 1　Sample distribution and collection information

序号<br>No.	采样地<br>Sampling site	海拔<br>Elevation//m	经度<br>Longitude	纬度<br>Latitude	植物<br>Plant	分离组织<br>Detached tissue	采集个体数<br>Number of samples
1	拉萨墨竹工卡县日多乡	4 250	92°10′38″	29°42′16″	米林杜鹃、雪层杜鹃	根、茎、叶、花	各 6 株
2	林芝色季拉山	4 442	92°38′11″	29°37′26″	髯花杜鹃、雪层杜鹃、雪山杜鹃(白花与红花)	根、茎、叶、花	各 6 株
3	日喀则亚东县帕里镇	4 311	89°07′42″	27°47′32″	雪层杜鹃	根、茎、叶	各 6 株
4	日喀则亚东县	4 110	89°02′31″	27°37′06″	雪层杜鹃	根、茎、叶	各 6 株

1.3　杜鹃属植物内生真菌的鉴定　通过改良的 CTAB 法[17]提取杜鹃属植物内生真菌的 DNA。用通用引物 ITS1(5′-TCCG TAGGTGAACCTGCGG-3′)和 ITS4(5′-TCCTCCGCTTATTGATATGC-3′)进行扩增。扩增条件:94 ℃预变性 5 min,94 ℃变性 45 s,52 ℃复性 45 s,72 ℃延伸 90 s,重复 35 次,72 ℃延伸 10 min,4 ℃低温保存。将 PCR 反应得到的产物经 1%琼脂糖凝胶电泳检测后,送至生工生物工程(成都)股份有限公司进行测序,测序后获得的 ITS 序列在 NCBI 中用 BLAST 进行同源性序列比对,以此进行内生真菌的鉴定。

1.4　内生真菌群落多样性分析　通过 Shannon 多样性指数(Shannon-Weiner diversity index,H')、辛普森指数(Simpson,D)、均匀度指数(evenness,E)、定殖率(colonization rate,CR)、分离率(isolation rate,IR)和分离频率(isolation frequency,IF)[18-23]分析杜鹃内生真菌的群落组成和多样性。该研究分别采用 R 语言、Origin 2021 和 Excel 软件对杜鹃属植物内生真菌进行分析和绘图。

2　结果与分析

2.1　杜鹃花属植物内生真菌种类　杜鹃属植物 720 个组织块中分离得到 336 株内生真菌,经过分子生物学鉴定,将 336 株内生真菌鉴定为子囊菌门、毛霉菌门 2 门,毛霉菌纲、座囊菌纲、粪壳菌纲、散囊菌纲、锤舌菌纲 5 纲,10 目 16 科 21 属 55 种(表 2)。

表 2　杜鹃属植物内生真菌的种类

Table 2　Types of endophytic fungi in *Rhododendron* plants

门<br>Phylum	纲<br>Class	目<br>Order	科<br>Family	属<br>Genus	种<br>Species	分离频率<br>IF//%
毛霉菌门<br>Mucoromycota	毛霉菌纲<br>Mucoromycetes	毛霉目<br>Mucorales	毛霉科<br>Mucoraceae	放射毛霉属 *Actinomucor*	雅致放射毛霉 *Actinomucorelegans*	0. 65
				毛霉属 *Mucor*	*Mucor* sp.	0. 98
子囊菌门<br>Ascomycota	座囊菌纲<br>Dothideomycetes	格孢腔菌目<br>Pleosporales	格孢菌科<br>Pleosporineae	链格孢属 *Alternaria*	链格孢霉 *Alternaria alternata*	1. 95
					多形性链格孢菌 *Alternaria multiformis*	0. 33
					极细链格孢菌 *Alternaria tenuissima*	0. 98
					芸苔链格孢菌 *Alternaria brassicae*	0. 33
				弯胞属 *Curvularia*	膝曲弯孢 *Curvularia geniculata*	0. 33
			多孢菌科<br>Pleosporineae	附球菌属 *Epicoccum*	黑附球菌 *epicoccum nigrum*	0. 65
			隔孢假壳科<br>Didymosphaeriaceae	亚隔孢壳属 *Didymella*	*Didymella* sp.	0. 33
		枝孢霉目<br>Cladosporiales	枝孢霉科<br>Cladosporiaceae	枝孢霉属 *Cladosporium*	芽枝状枝孢 *Cladosporium pseudocladosporioides*	0. 33
	粪壳菌纲<br>Sordariomycetes	炭角菌目<br>Xylariomycetidae	梨孢假壳科<br>Apiosporaceae	略氏端隔孢壳菌属 *Apiospora*	*Apiospora marii*	0. 33
			炭角菌科<br>Xylariales	炭球菌属 *Daldinia*	炭球菌 *Daldinia concentrica*	0. 33
					轮层炭壳菌 *Daldinia loculata*	0. 65
		粪壳菌目<br>Sordariales	粪壳菌科<br>Sordariaceae	脉孢菌属 *Neurospora*	四孢脉孢菌 *Neurospora tetraspora*	0. 33
			毛壳科<br>Chaetomiaceae	毛壳属 *Chaetomium*	球毛壳霉 *Chaetomium globosum*	0. 33
		肉座菌目<br>Hypocreales	虫草菌科<br>Cordycipitaceae	白僵菌属 *Beauveria*	球孢白僵菌 *Beauveria bassiana*	29. 64
				鳞翅虫草属 *Samsoniella*	*Samsoniella alpina*	0. 33
			肉座菌科<br>Hypocreaceae	木霉属 *Trichoderma* sp.	橘绿木霉 *Trichoderma citrinoviride*	1. 63
					里氏木霉 *Trichoderma reesei*	0. 98
					侧耳木霉 *Trichoderma pleuroticola*	0. 33
					多孢木霉 *Trichoderma polysporum*	0. 33

接下表

(C)1994-2023 China Academic Journal Electronic Publishing House. All rights reserved.　http://www.cnki.net

续表 2

门<br>Phylum	纲<br>Class	目<br>Order	科<br>Family	属<br>Genus	种<br>Species	分离频率<br>IF//%
			麦角菌科<br>Clavicipitaceae	绿僵菌属 *Metarhizium*	金龟子绿僵菌 *Metarhizium anisopliae*	0.65
		间座壳菌目<br>Diaporthales	黑腐皮壳科<br>Valsaceae	壳囊孢属 *Cytospora*	*Cytospora salicacearum*	0.33
					Cytospora tritici	0.33
		锥毛壳目<br>Coniochaetales	锥毛壳科<br>Coniochaetaceae	锥毛壳属 *Coniochaeta*	*Coniochaeta* sp.	0.65
	散囊菌纲<br>Eurotiomycetes	散囊菌目<br>Eurotiales	发菌科<br>Trichocomaceae	曲霉属 *Aspergillus*	冠突曲霉 *Aspergillus cristatus*	0.33
					黑曲霉 *Aspergillus niger*	23.45
					诺米亚曲霉菌 *Aspergillus nomia*	0.33
					多育曲霉 *Aspergillus proliferans*	0.33
					土曲霉 *Aspergillus terreus*	1.95
					塔宾曲霉菌 *Aspergillus tubingensis*	3.91
					杂色曲霉 *Aspergillus versicolor*	0.65
					百岁兰曲霉菌 *Aspergillus welwitschiae*	0.33
				青霉属 *Penicillium*	沙门柏干酪青霉 *Penicillium camemberti*	1.63
					产黄青霉 *Penicillium chrysogenum*	3.91
					二元青霉菌 *Penicillium dipodomyicola*	1.95
					菲姆青霉菌 *Penicillium fimorum*	0.33
					光孢青霉 *Penicillium glabrum*	1.30
					Penicillium goetzii	0.33
					灰黄青霉 *Penicillium griseofulvum*	3.26
					奥尔森青霉 *Penicillium olsonii*	1.63
					波兰青霉 *Penicillium polonicum*	1.63
					产红青霉 *Penicillium rubens*	0.33
					小刺青霉 *Penicillium spinulosum*	0.65
					亚紫青霉 *Penicillium subrubescens*	0.65
					托姆青霉菌 *Penicillium thomii*	2.93
					荨麻青霉 *Penicillium urticae*	1.30
					威灵顿青霉菌 *Penicillium wellingtonense*	0.33
					普通青霉菌 *Penicillium commune*	0.33
					小本青霉 *Penicillium onobense*	0.33
					铅色青霉 *Penicillium lividum*	0.33
					皮落青霉 *Penicillium crustosum*	0.33
				篮状菌属 *Talaromyces*	黄篮状菌 *Talaromyces flavus*	0.33
					嗜松篮状菌 *Talaromyces pinophilus*	0.65
	锤舌菌纲<br>Leotiomycetes	Leotiomycetes incertae sedis	假散囊菌科<br>Pseudeurotiaceae	假散囊菌属<br>*Pseudeurotium*	*Pseudeurotium* sp.	0.33

2.2 杜鹃花属植物不同组织内生真菌的群落组成 从图 1 可以看出,4 种杜鹃属植物根部组织 192 块中分离出 93 株内生真菌,隶属于 13 属,其优势属为曲霉属 *Aspergillus*(40.86%)、白僵菌属 *Beauveria*(19.35%)和青霉属 *Penicillium*(16.13%)。茎部组织 192 块中分离出 94 株内生真菌,其优势属为曲霉属(37.23%)、白僵菌属(25.53%)和青霉属(25.53%)。叶部组织 192 块分离出 80 株内生真菌,其优势属为白僵菌属(38.75%)、青霉属(28.75%)和曲霉属(21.25%)。花组织 144 块中分离出 69 株内生真菌,优势属为曲霉属(33.33%)、白僵菌属(30.43%)和青霉属(23.19%)。

研究结果还发现根部组织特有属为锥毛壳属 *Coniochaeta*(2.15%)、弯胞属 *Curvularia*(1.08%)、炭球菌属 *Daldinia*(4.30%)、脉孢菌属 *Neurospora*(1.08%)、鳞翅虫草属 *Samsoniella*(1.08%)、篮状菌属 *Talaromyces*(3.21%)和壳囊孢属 *Cytospora*(2.15%)。茎组织特有属为略氏端隔孢壳菌属 *Apiospora*(1.06%)和假散囊菌属 *Pseudeurotium*(1.06%)。叶组织特有属为枝孢霉属 *Cladosporium*(1.25%)、亚隔孢壳属 *Didymella*(1.25%)和绿僵菌属 *Metarhizium*(2.50%)。花组织特有属为毛壳属 *Chaetomium*(1.45%)。

4 种杜鹃属植物根、茎、叶、花组织内生真菌优势种都是曲霉属、白僵菌属和青霉属(图 2)。米林杜鹃中分离出 41 株内生真菌,分为 4 属,分别为链格孢属(*Alternaria*)、曲霉属、白僵菌属和青霉属;髯花杜鹃分离出 67 株内生真菌,分为 5 属,分别为链格孢属、曲霉属、白僵菌属、青霉属和篮状菌属;雪层杜鹃分离出 119 株内生真菌,分为 10 属,分别为放射毛霉属(*Actinomucor*)、链格孢属、曲霉属、白僵菌属、青霉属、附球菌属(*Epicoccum*)、绿僵菌属、毛霉属(*Mucor*)、鳞翅虫草属和木霉属(*Trichoderma*);雪山杜鹃分离出 99 株内生真菌,分为 17 属,分别为链格孢属、略氏端隔孢壳菌属、曲霉属、白僵

(C)1994-2023 China Academic Journal Electronic Publishing House. All rights reserved. http://www.cnki.net

菌属、枝孢霉属、锥毛壳属、弯胞属、毛壳属、壳囊孢属、炭球菌属、亚隔孢壳属、青霉属、脉孢菌属、假散囊菌属、附球菌属、毛霉属和木霉属。

4 种杜鹃属植物根、茎、叶、花组织内生真菌的特有属共有 12 属，分别是枝孢霉属 、锥毛壳属、弯胞属、炭球菌属、亚隔孢壳属、绿僵菌属、脉孢菌属、假散囊菌属、鳞翅虫草属、篮状菌属、毛壳属和壳囊孢属。在根部组织中，链格孢属和篮状菌属为髯花杜鹃的特有属，锥毛壳属、弯胞属、炭球菌属、附球菌属、脉孢菌属和壳囊孢属为雪山杜鹃的特有属，鳞翅虫草属为雪层杜鹃特有属；在茎部组织中，放射毛霉属为雪层杜鹃特有属，链格孢属、略氏端隔孢壳菌属、毛霉属和假散囊菌属为雪山杜鹃的特有属；在叶部组织中，放射毛霉属、链格孢属、略氏端隔孢壳菌属、绿僵菌属和木霉属为雪层杜鹃特有属，枝孢霉属、亚隔孢壳属为雪山杜鹃的特有属；在花组织中，毛霉属为雪层杜鹃特有属，毛壳属为雪山杜鹃的特有属。

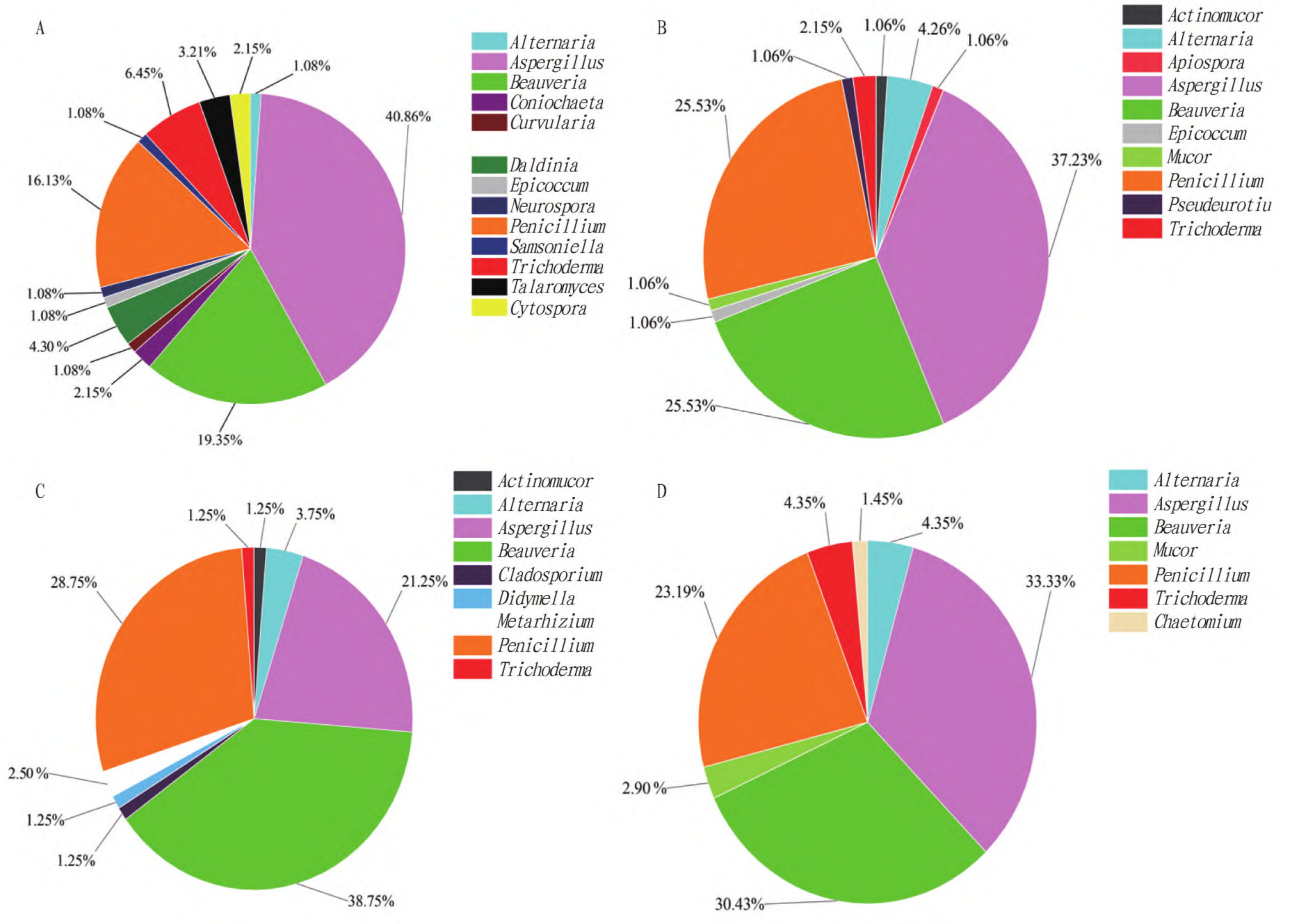

注：A. 根；B. 茎；C. 叶；D. 花。

Note：A. Root；B. Stem；C. Leaf；D. Flower.

图 1　杜鹃不同组织内生真菌的群落组成

Fig. 1　Community composition of endophytic fungi in different tissues of *Rhododendron*

从表 3 可以看出，杜鹃属植物不同组织部位的定殖率分别为茎 92.71%、根 91.15%、花 90.97%、叶 90.63%，分离率分别为茎 48.96%、根 48.44%、花 47.92%、叶 41.67%。

2.3　杜鹃属植物内生真菌的多样性分析　4 种杜鹃属植物多样性分析结果（表 4）显示，Shannon 多样性指数（H'）从大到小依次为雪山杜鹃>雪层杜鹃>髯花杜鹃>米林杜鹃，多样性最高的是雪山杜鹃，最低的是米林杜鹃。辛普森指数（D）从大到小依次为雪山杜鹃>雪层杜鹃>髯花杜鹃>米林杜鹃。将多样性数据进行显著性差异分析，P 值为 0.40，说明 4 种杜鹃属植物内生真菌无显著差异。

2.4　杜鹃属植物不同组织内生真菌的多样性分析　从杜鹃属植物不同组织部位（根、茎、叶、花）的多样性指数（表 5）可以看出，Shannon 多样性指数（H'）从大到小依次为根>茎>叶>花，多样性最高的是根，最低的是花。辛普森指数（D）从大到小依次为根>茎=花>叶。将多样性数据进行显著性差异分析，P 值为 0.39，说明 4 种杜鹃属植物不同组织内生真菌无显著差异。

3　讨论

该研究对西藏 4 种杜鹃属植物 720 个组织块进行分离得到 336 株内生真菌，鉴定为 21 属 55 种。根、茎、叶和花组织中优势属都有曲霉属、白僵菌属和青霉属。其中曲霉属和青霉属为许多药用的草本植物和木本植物的优势种[9]。有研究报道，青霉属、曲霉属、木霉属、链格孢属等在亮毛杜鹃（*R. microphyton* Franch）根际内生真菌中存在，其中青霉属是

(C)1994-2023 China Academic Journal Electronic Publishing House. All rights reserved.　http://www.cnki.net

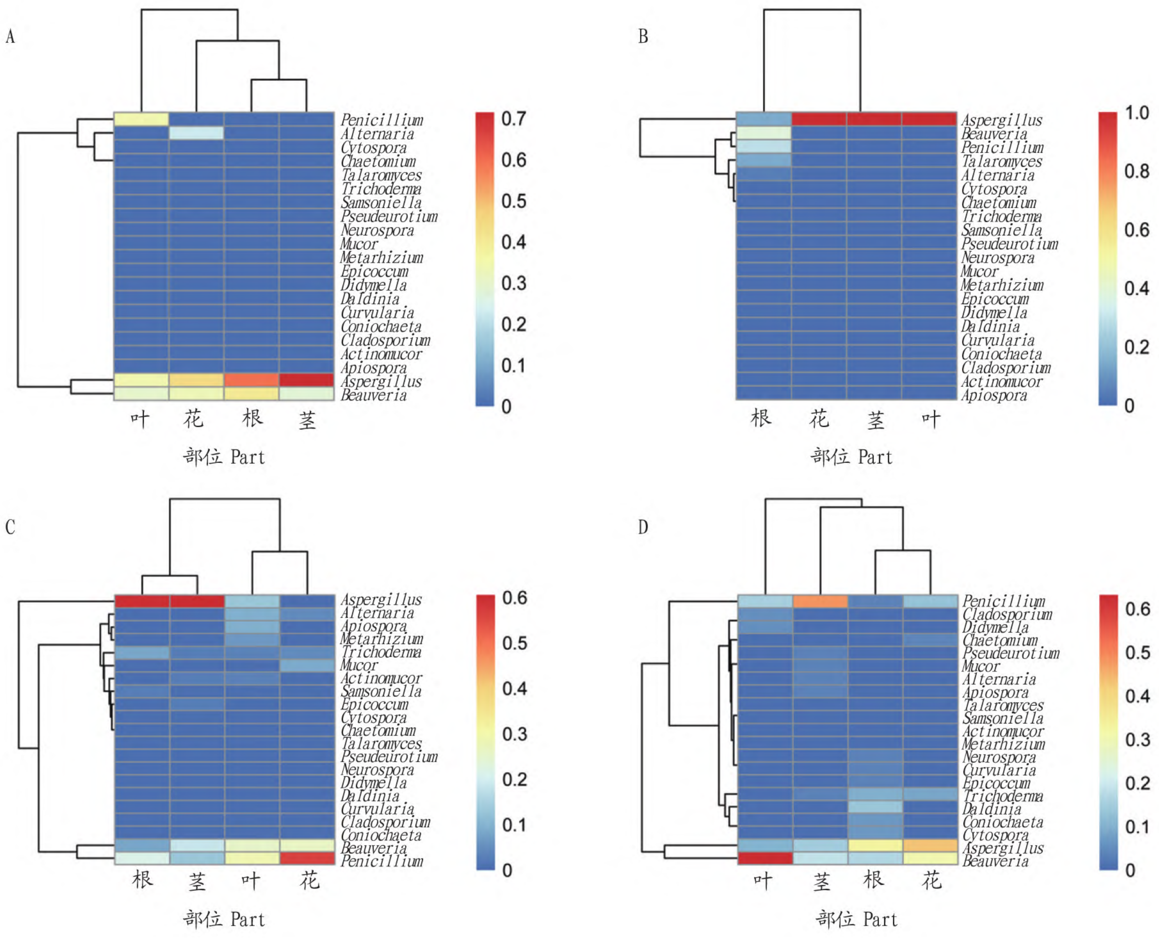

注:A. 米林杜鹃;B. 髯花杜鹃;C. 雪层杜鹃;D. 雪山杜鹃。

Note:A. *R. mainlingense*;B. *R. anthopogon*;C. *R. nivale*;D. *R. aganniphum*.

图 2 4 种杜鹃属植物不同组织内生真菌的相对丰度热图

Fig. 2 Heat map of relative abundance of endophytic fungi in different tissues of four *Rhododendron* species

表 3 不同分离组织部位杜鹃内生真菌的定殖率和分离率

Table 3 Colonization rate and isolation rate of endophytic fungi of *Rhododendron* species in different isolated tissues

部位 Part	组织块数 Number of tissues detected	有菌组织块数 Number of tissues colonized by fungi	分离菌株数 Number of isolates	定殖率 CR//%	分离率 IR//%	分离频率 IF//%
根 Root	192	175	93	91. 15	48. 44	27. 68
茎 Stem	192	178	94	92. 71	48. 96	27. 98
叶 Leaf	192	174	80	90. 63	41. 67	23. 81
花 Flower	144	131	69	90. 97	47. 92	20. 54

表 4 杜鹃属植物内生真菌的多样性指数

Table 4 Diversity index of endophytic fungi from *Rhododendron* plant

植物 Plant	物种数 Species number	H'指数 H' index	D 指数 D index	E 指数 E index
米林杜鹃 *R. mainlingense*	4	1. 17	0. 65	0. 84
雪层杜鹃 *R. nivale*	21	1. 81	0. 77	0. 60
髯花杜鹃 *R. anthopogon*	5	1. 36	0. 71	0. 84
雪山杜鹃 *R. aganniphum*	17	1. 96	0. 80	0. 69

优势属[24];青霉属、木霉属在杜鹃种(*R. simsii*)根部内生真菌中发现,且为优势属[25]。子囊菌和木霉属在马缨杜鹃(*Rhododendron delavayi*)内生真菌中被报道,其中子囊菌为优势菌群[26]。曲霉属、拟隐壳孢属、镰刀菌属和壳多胞菌属在西藏米拉山雪层杜鹃的内生真菌中被分离发现[16]。该研究发现4 种杜鹃属植物内生真菌共有类型为曲霉属、青霉属、链格孢属、白僵菌属。其中白僵菌属在其他杜鹃属植物内生真菌鉴定中鲜见报道。

表 5 不同分离组织部位杜鹃内生真菌的多样性指数

Table 5 Diversity index of endophytic fungi from different tissues of *Rhododendron*

部位 Part	物种数 Species number	H'指数 H' index	D 指数 D index	E 指数 E index
根 Root	29	2. 63	0. 86	0. 78
茎 Stem	24	2. 35	0. 83	0. 74
叶 Leaf	21	2. 26	0. 80	0. 74
花 Flower	18	2. 23	0. 83	0. 77

(C)1994-2023 China Academic Journal Electronic Publishing House. All rights reserved. http://www.cnki.net

于洪佳等[27]研究报道青霉属和曲霉属具有抗菌、抗癌、抗肿瘤、抗氧化、杀虫、免疫调节等功效。曲霉属和青霉属都有生物碱类、聚酮类、萜类、肽类、固醇类和脑苷脂类等化合物[28]。从枝孢霉属的次级代谢产物中分离出甾醇、倍半萜类、生物碱类、二酮哌嗪、芳香酸等化合物[29]。篮状菌属的次级代谢产物有萜类、生物碱类、聚酮类、聚酯类、醌类和甾体类,具有抗菌、抗病毒、抗真菌、抗肿瘤活性、生物防治和产生酶活等作用,在食品、环境、农业和医药等方面发挥了重要作用[30]。毛壳属真菌代谢产物的主要类型为细胞松弛素类和唑啉酮两大类,包含细胞松弛素、唑啉酮类、二酮哌嗪类、色原酮、蒽醌等结构类型,表现出抗微生物、抗氧化、免疫抑制等生物活性,具有抗菌、抗肿瘤、抗病毒等作用[31]。

综上所述,该研究对西藏地区不同种杜鹃属植物的不同组织内生真菌进行分离鉴定,发现种间真菌多样性差异不明显,同种植物不同部位内生真菌差异不明显,说明 4 种内生真菌组成相似,且优势属都有曲霉属、白僵菌属和青霉属。该研究结果为下一步内生真菌代谢产物研究提供基础。

参考文献

[1] 中国科学院青藏高原综合考察队. 西藏植物志:第 3 卷[M]. 北京:科学出版社,1983:552-562.
[2] 伍志辉. 羊踯躅根(丸)治疗类风湿性关节炎一例探讨[J]. 中国药业,1996,5(9):42-43.
[3] 国家药典委员会. 中华人民共和国药典:2010 年版 一部[S]. 北京:中国医药科技出版社,2010:210.
[4] 陈冀胜,郑硕. 中国有毒植物[M]. 北京:科学出版社,1987:216-239.
[5] STONE J K,BACON C W,WHITE J F,Jr. An overview of endophytic microbes:Endophytism defined[M]//BACON C W,WHITE J. Microbial endophytes. Boca Raton:CRC Press,2000:3-29.
[6] SMITH S E,READ D J. Mycorrhizal symbiosis[M]. San Diego:Academi Press,2008:419-457
[7] 郭顺星. 药用植物内生真菌研究现状和发展趋势[J]. 菌物学报,2018,37(1):1-13.
[8] 宁祎,李艳玲,李媛,等. 桃儿七茎叶组织内生真菌多样性[J]. 生态学报,2017,37(15):5157-5166.
[9] 谭小明,周雅琴,陈娟,等. 药用植物内生真菌多样性研究进展[J]. 中国药学杂志,2015,50(18):1563-1580.
[10] UPSON R,READ D J,NEWSHAM K K. Nitrogen form influences the response of *Deschampsia antarctica* to dark septate root endophytes[J]. Mycorrhiza,2009,20(1):1-11.
[11] 程茂高,乔卿梅,魏志华,等. 中药材仓储害虫及其绿色防控技术研究进展[J]. 中药材,2016,39(8):1917-1921.
[12] FRANTZESKAKIS L,DI PIETRO A,REP M,et al. Rapid evolution in plant-microbe interactions-a molecular genomics perspective[J]. New Phytol,2020,225(3):1134-1142.
[13] PHILIPPOT L,RAAIJMAKERS J M,LEMANCEAU P,et al. Going back to the roots:The microbial ecology of the rhizosphere[J]. Nat Rev Microbiol,2013,11(11):789-799.
[14] VANDENKOORNHUYSE P,QUAISER A,DUHAMEL M,et al. The importance of the microbiome of the plant holobiont[J]. New Phytol,2015,206(4):1196-1206.
[15] STROBEL G,STIERLE A,STIERLE D,et al. *Taxomyces andreanae*, a proposed new taxon for a bulbilliferous hyphomycete associated with Pacific yew(*Taxus brevifolia*)[J]. Mycotaxon(USA),1993,47(71):71-80.
[16] 郭顺星. 药用植物内生真菌生物学[M]. 北京:科学出版社,2016.
[17] 张弘弛. 两种药用植物内生真菌次生代谢产物及其生物活性的研究[D]. 西安:陕西科技大学,2012.
[18] PIELOU E C. Ecological diversity[M]. New York:John Wiley and Sons Inc.,1975:1-165.
[19] HATA K,FUTAI K. Endophytic fungi associated with healthypine needles and needles in fested by the pine needle gall midge,*Thecodiplosis japonensis*[J]. Can J Bot,1995,73(3):384-390.
[20] YUAN Z L,ZHANG C L,LIN F C,et al. Identity,diversity,and molecular phylogeny of the endophytic mycobiota in the roots of rare wild rice(*Oryza granulate*)from a nature reserve in Yunnan,China[J]. Appl Environ Microbiol,2010,76(5):1642-1652.
[21] 罗鑫,于存. 贵州马尾松内生真菌多样性[J]. 菌物学报,2021,40(3):531-546.
[22] 顾美英,张志东,唐光木,等. 黑果枸杞不同组织内生真菌群落组成及生态功能分析[J]. 菌物学报,2022,41(8):1254-1267.
[23] BOYLE C,GÖTZ M,DAMMANN-TUGEND U,et al. Endophyte-host interactions III. Local vs. systemic colonization[J]. Symbiosis,2001,31(4):259-281.
[24] 李雪玲. 亮毛杜鹃根际真菌与内生真菌的多样性研究[J]. 楚雄师范学院学报,2004,19(3):87-90,132.
[25] 熊丹,欧静,李林盼,等. 黔中地区马尾松林下杜鹃根部内生真菌群落组成及其生态功能[J]. 生态学报,2020,40(4):1228-1239.
[26] 龙毅. 三种杜鹃属植物菌根真菌的研究[D]. 贵州:贵州大学,2008.
[27] 于洪佳,薛雅馨,洪葵,等. 一株红树林曲霉菌 *Aspergillus* sp. WHUF0343 的次级代谢产物研究[J]. 微生物学报,2022,62(7):2658-2670.
[28] 闫慧娇. 两株黄槿内生真菌次生代谢产物研究[D]. 青岛:中国科学院研究生院(海洋研究所),2010.
[29] 常俊男,田晓清,樊成奇,等. 两株南极枝孢霉属真菌 *Cladosporium* sp. NJF4 和 NJF6 的次级代谢产物研究[J]. 极地研究,2020,32(1):60-67.
[30] 陈仲巍,林风娇,陈彬彬,等. 篮状菌属微生物次级代谢产物研究进展[J]. 生物资源,2022,44(4):362-369.
[31] 徐国波,张青艳,周孟. 毛壳属真菌的次生代谢产物及其生物活性研究进展[J]. 天然产物研究与开发,2018,30(3):515-525.

(上接第 137 页)

去寻找体系中的规律性[11]。基于灰色系统以上特点,该研究以相对关联度为测度,建立评价半枝莲药材等级规格质量的模型,为半枝莲药材等级规格内涵的评价提供一种新的评价方法。

参考文献

[1] 国家药典委员会. 中华人民共和国药典:2020 年版 一部[S]. 北京:中国医药科技出版社,2020:122.
[2] 黄雅娜,张铃,林炜. 半枝莲对人结肠癌细胞 SW620 增殖和凋亡的影响[J]. 福建中医药,2020,51(2):33-35,91.
[3] 田新宇,范翠梅,渠田田,等. 半枝莲总黄酮中 7 种成分的含量测定及抗肿瘤活性[J]. 中国实验方剂学杂志,2017,23(1):53-59.
[4] 王彩虹,尹宁,王柯静,等. 基于 AMPK 通路研究半枝莲多糖对宫颈癌移植瘤小鼠肿瘤组织血管生成的作用机制[J]. 中药药理与临床,2022,38(2):74-78.
[5] 郭姗姗,时宇静,高英杰,等. 半枝莲总黄酮抗副流感病毒的作用机制[J]. 药学学报,2009,44(12):1348-1352.
[6] 冯德富,李小沙. 复方半枝莲汤联合阿德福韦酯治疗慢性乙型肝炎疗效观察[J]. 实用中医内科杂志,2010,24(2):72-73,76.
[7] 农训学. 半枝莲采收加工[N]. 民族医药报,2006-12-22(003).
[8] 武孔云. 中药材种养关键技术丛书[M]. 南京:江苏科学技术出版社,2001:295.
[9] 张洪坤,王其丰,郭长达,等. 不同加工方法牡丹皮中 7 种指标性成分的含量测定及质量评价[J]. 中国药房,2018,29(22):3063-3068.
[10] 张洪坤,路丽,黄玉瑶,等. 牡丹皮不同采收期质量综合评价研究[J]. 世界科学技术-中医药现代化,2019,21(2):240-247.
[11] 李少泓,夏鹏飞,马肖,等. 基于灰色关联分析方法评价当归药材质量[J]. 中药材,2012,35(11):1742-1746.
[12] 张洪坤,路丽,黄玉瑶,等. 关于半枝莲质量标准中不同部位占比的研究[J]. 安徽医药,2022,26(3):458-462.
[13] 李硕,李成义,李敏,等. 基于灰色关联分析方法评价商品甘草药材质量[J]. 中国实验方剂学杂志,2015,21(1):89-94.
[14] 龚雨虹,罗光明,张风波,等. 基于灰色关联度法评价栀子药材质量[J]. 中国实验方剂学杂志,2017,23(13):74-79.
[15] 李莉,常欣,陈志禹. 基于熵权法和灰色关联度法的川贝母质量评价研究[J]. 中药材,2021,44(2):387-393.
[16] 邹佳莉,王康宇,何文媛,等. 基于熵权法和灰色关联度分析对当归道地产区划分研究[J]. 中药材,2022,45(5):1173-1178.
[17] 林钦贤,梁伟龙,王斌,等. 基于灰色关联分析法评价广藿香药材质量[J]. 安徽农业科学,2020,48(2):213-218,263.

(C)1994-2023 China Academic Journal Electronic Publishing House. All rights reserved.　http://www.cnki.net
