# Supplementary material for: Metabolomics Based on UPLC-MS/MS Revealed the Metabolic Differences Among Four Species of Rhododendrons in Linzhi, Xizang
Source: Metabolites. 2026 Mar 30;16(4):226. doi: 10.3390/metabo16040226 (PMC13117825; doi:10.3390/metabo16040226)
Supplement: Supplementary file 1 [file metabolites-16-00226-s001.zip › Supplementary documents/Supplementary References/7.Zheng W, Pan G, Xu A, et al (1995) Preliminary study on the germplasm resources of Rhododendron in Sejila Mountain, Xizang..pdf]

园 艺 学 报 1995, 22(2): 166—170
Acta Horticulturae Sinica

西藏色季拉山杜鹃花种质资源的初步研究*

郑维列　潘　刚　徐阿生　罗大庆
(西藏高原生态研究所,林芝 860000)

提　要　色季拉山杜鹃花有25个种及变种,其中7种分布于东坡,3种分布于西坡,4种分布于山顶地带,11种在东西坡均有分布。其生境类型可分为林缘灌丛型、森林型、高山灌丛型3类,以森林型种类居多,占48%。其观赏类群可分为香花类、团花类、雅致类和黄花类4群,以香花类和黄花类观赏价值最高,雅致类最易被驯化利用。

关键词　西藏;　杜鹃花;　种质资源;　生境类型;　观赏评价

杜鹃花(*Rhododendron* L.)全世界约有850种,我国约460种,西藏产170种左右,占世界的1/5[1]。研究西藏杜鹃的分布与生境,对开发、利用该地区的杜鹃资源有重要意义。色季拉山位于西藏东南部,系念青唐古拉山南伸的余脉,与喜马拉雅山向东发展的山系相连,其基带属湿润山地暖温带、半湿润山地温带气候[3];其杜鹃花区系反映出藏东横断山区与藏南东喜马拉雅南翼两大杜鹃种质丰富区间的联系。

1　杜鹃花种质资源

色季拉山有杜鹃花属植物25种及变种,检索如下:

西藏色季拉山杜鹃花属植物分种检索表

1. 叶片具鳞片(杜鹃亚属 Subgen. *Rhododendron*)
  2. 叶长8cm以下,宽3cm以下
    3. 鳞片边缘锐裂,叶下面暗红褐色 ……………………………………………… (1) 林芝杜鹃 *R. nyingchiense* R.C. Fang et H. Huang
    3. 鳞片全缘或有微齿,叶下面不为暗红褐色
      4. 花冠黄色带杏红色……………………………… (2) 三花杜鹃 *R. trifloum* Hook. f.
      4. 花冠不为黄色
        5. 叶缘具细圆齿,有时疏生刚毛 …… (3) 草莓花杜鹃 *R. fragariflorum* Ward
        5. 叶全缘
          6. 叶背鳞片色单一,或深浅色分异不显著
            7. 花冠外面被毛 ……………………… (4) 石峰杜鹃 *R. scopulorum* Hutch.
            7. 花冠外面无毛
              8. 花冠外面被鳞片
                9. 花芽顶生,花梗长1cm以上,花冠辐状……………………………………………………………………(5) 鳞腺杜鹃 *R. lepidotum* Wall.
                9. 花芽腋生,花梗长0.5cm以下,花冠漏斗状………………………

收稿日期: 1994—07—06;　修回日期: 1994—08—24。
* 本研究在徐凤翔教授指导下完成,特此致谢!

(C)1994-2023 China Academic Journal Electronic Publishing House. All rights reserved.　http://www.cnki.ne

……………………………………………………… (6) 柳条杜鹃 *R. virgatum* Hook. f.

8. 花冠外面无鳞片

10. 花冠白色带淡红色,叶面有刚毛 ……………… (7) 睫毛杜鹃 *R. ciliatum* Hook. f.

10. 花冠紫红色,叶面无毛 ………………… (8) 山育杜鹃 *R. oreotrephes* W. W. Smith

6. 叶背鳞片明显 2 色

11. 叶长 10mm 以下,暗色鳞片多而均匀 ……… (9) 雪层杜鹃 *R. nivale* Hook. f.

11. 叶长 12mm 以上,暗色鳞片少而分散……………… (10) 散鳞杜鹃 *R. bulu* Hutch.

2. 叶长 10cm 以上,宽 4cm 以上

12. 叶柄连同中脉仅在下面隆起……………………………………………………………………………………(11) 大萼杜鹃 *R. megacalyx* Balf. f. et Ward

12. 叶柄连同中脉在两面隆起…………………………(12)木兰杜鹃 *R. nuttallii* Booth

1. 叶片不具鳞片(常绿杜鹃亚属 Subgen. *Hymenanthes*)

13. 叶柄具腺头刚毛 ……………………………… (13) 硬毛杜鹃 *R. hirtipes* Tagg

13. 叶柄不具腺头刚毛

14. 子房密生腺体

15. 花冠黄色 ………………………… (14) 黄杯杜鹃 *R. wardii* W. W. Smith

15. 花冠不为黄色

16. 花冠粉红色至白色,子房腺体具长柄……………………………………………………………(15) 喉斑杜鹃 *R. faucium* Chamberlain

16. 花冠深红色,子房腺体不具长柄……………………………………………………………………(16) 樱花杜鹃 *R. cerasinum* Tagg

14. 子房无腺体

17. 叶背无毛,侧脉清晰

18. 叶革质,侧脉在叶背隆起,子房无毛……………………………………………………(17) 光柱杜鹃 *R. tanastylum* Balf. f. et Ward

18. 叶纸质,侧脉在叶背不隆起,子房疏生平伏柔毛………………………………………(18) 红点杜鹃 *R. rubro-punctatum* T. l. Ming

17. 叶背被各式毛被,侧脉不显

19. 子房明显被毛…………………………………………………………………………………(19) 长叶川滇杜鹃 *R. traillianum* Forrest et W. W. Smith var. *dictyotum* (Balf. f. ex Tagg) Chamberlain

19. 子房无毛或微被毛

20. 叶背被灰白色羊皮紫质薄层毛被………………………………………(20)光蕊杜鹃 *R. coryanum* Togg et Forrst

20. 叶背毛被非羊皮纸质

21. 果狭长而呈镰状弯弓 ……………………………………………………(21) 紫玉盘杜鹃 *R. uvarifolium* Diels

21. 果粗短而直立或略弯

(C)1994-2023 China Academic Journal Electronic Publishing House. All rights reserved.　http://www.cnki.net

22. 叶柄被灰白色毛……………………………………………………………(22) 白背紫斑杜鹃 *R. principes* Bur. et Franch. var. *vellereum* (Hutch. ex Tagg) T.L. Ming

22. 叶柄无毛

23. 侧脉少于 12 对,叶柄长 1.5cm 以下,花冠粉红色至白色……………………………………………………………………………(23) 黄毛海绵杜鹃 *R. aganniphum* Balf. f. et Ward var. *flavorufum* (Balf. f. et Forrest) Chamberlain

24. 叶背毛被由灰白色变至褐色,不开裂……………………………………………………………………………(24) 薄毛海绵杜鹃 *R. aganiphum* Balf. f. et Ward var. *schizopeplum* (Balf. f. et Forrest) T.L. Ming

23. 侧脉多于 13 对,叶柄长 1.5cm 以上,花冠紫色至白色 ……………………………………………………………… (25) 钟花杜鹃 *R. campanulatum* D. Don

在上述各类杜鹃中,山育杜鹃(原产芒康、波密)、木兰杜鹃(原产波密)、薄毛海绵杜鹃(原产芒康、察隅、米林)、黄毛海绵杜鹃(原产察隅)、长叶川滇杜鹃(原产芒康、察隅、米林)、光柱杜鹃(原产察隅)、大萼杜鹃(原产察隅)、钟花杜鹃(原产喜马拉雅南麓)8 种及变种为林芝新分布;林芝杜鹃、草莓花杜鹃、散鳞杜鹃、石峰杜鹃、白背紫斑杜鹃、红点杜鹃、喉斑杜鹃、硬毛杜鹃 8 种为西藏特有种。

据文献记载[2],林芝还产林生杜鹃(*R. lanigerum* Tagg)、长蕊杜鹃(*R. ramsdenianum* Cowan)、弯柱杜鹃(*R. campylogynum* Franch)、毛冠杜鹃(*R. laudandum* Cowan)4 种。调查中未发现以上 4 种,故本文未予列入。

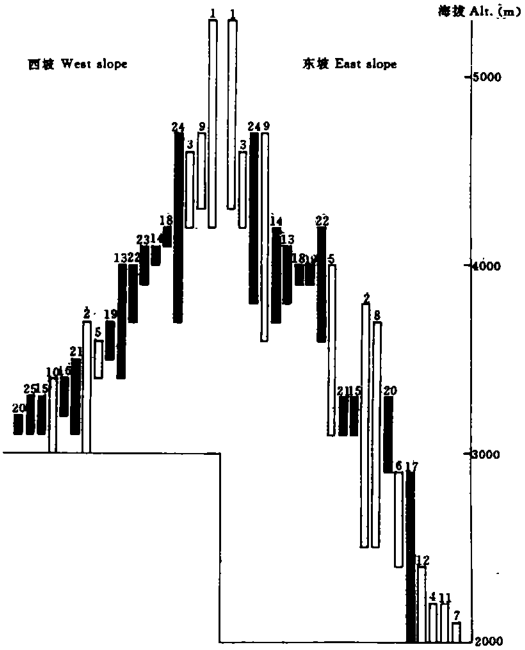

图 色季拉山杜鹃花垂直分布示意图

* 图中数码与检索表中相同。黑柱代表常绿杜鹃亚属,白柱代表杜鹃亚属。

Figure. Illustrated drawing for the vertical distribution of rhododendrons on Shergyla Mountain

The numerals in drawing are the same as in key—list. The solid posts stand for Subgen. *Hymenanthes* and the hollow ones stand for Subgen. *Rhododendron.*

2 资源分布

2.1 垂直分布

东坡下切至海拔 2000m 的泊隆藏布峡谷,属山地亚热带与山地暖温带交汇带、常绿落叶阔叶混交林地带,分布有光柱杜鹃、石峰杜鹃、木兰杜鹃、大萼杜鹃、睫毛杜鹃。海拔 2400~2900m 松林、针阔混交林地带分布有柳条杜鹃、鳞腺杜鹃、三花杜鹃、山育杜鹃、光柱杜鹃。

东坡海拔 2900m 以上及西坡海拔 3000m 以上至山顶海拔 5300m,有松林、云杉、冷杉林等、高山灌丛、草甸及冰碛石滩[3]。本山区 76%的杜鹃花种类在该区域分布(图)。

2.2 水平分布

(C)1994-2023 China Academic Journal Electronic Publishing House. All rights reserved. http://www.cnki.ne

光柱杜鹃、柳条杜鹃、山育杜鹃、大萼杜鹃、木兰杜鹃、睫毛杜鹃、石峰杜鹃7种仅分布于东坡,占总数的28%。钟花杜鹃、樱花杜鹃、散鳞杜鹃3种仅分布于西坡,占12%。林芝杜鹃、雪层杜鹃、草莓花杜鹃、薄毛海绵杜鹃4种分布于海拔4200m以上的山顶地带,占16%。光蕊杜鹃、紫玉盘杜鹃、喉斑杜鹃、红点杜鹃、黄杯杜鹃、硬毛杜鹃、白背紫斑杜鹃、长叶川滇杜鹃、黄毛海绵杜鹃、鳞腺杜鹃、三花杜鹃11种在两坡均有分布,占总数的44%。

3 生境类型

3.1 林缘灌丛型

属于该生境型的种类生于林缘灌丛或林中空地,喜光,稍耐荫。

3.1.1 温暖亚型　有睫毛杜鹃、石峰杜鹃、大萼杜鹃和木兰杜鹃4种,生长于东坡海拔2000～2200(2400)m常绿落叶阔叶混交林林缘灌丛,或向林中延伸,主要建群中有察隅润楠(*Machilus chayuensis* S. Lee)、云南鹅耳枥(*Carpinus monbeigiana* Hand. —Mazz.)等。

3.1.2 温凉亚型　有山育杜鹃、鳞腺杜鹃、散鳞杜鹃、三花杜鹃和柳条杜鹃5种,生长于海拔(2400)2500～3500(4000)m阳坡灌丛、林缘灌丛、林中空地及高山松林下,为分布幅最宽的类型,主要伴生种有川滇高山栎(*Quercus aquifolioides* Rehd. et Wils.)、多蕊金丝桃(*Hypericum hookerianum* Wight et Arn.)、楔叶绣线菊(*Spiraea canescens* D. Don)等。

3.2 森林型

属于该生境型的种类生于林下及林缘,喜肥,耐荫。

3.2.1 温暖亚型　仅光柱杜鹃1种,生长于东坡海拔2000～2900m常绿阔叶林、常绿落叶阔叶混交林、针阔混交林、松林中。该群种中常绿阔叶树有通麦栎(*Quercus tungmaiensis* Y. T. Chang)、落叶阔叶树有长穗桦(*Betula cylindrostachya* Lindl.)、针叶树有华山松(*Pinus armandi* Franch.)等。

3.2.2 温凉亚型　有钟花杜鹃、喉斑杜鹃、樱花杜鹃、光蕊杜鹃和紫玉盘杜鹃5种。生长于海拔3000～3200(3400)m云杉、冷杉林中,主要伴生种有杯萼忍冬(*Lonicera inconspicua* Batal.)、西藏箭竹(*Sinarundinaria setosa* Yi.)等。

3.2.3 寒冷亚型　有黄杯杜鹃、红点杜鹃、硬毛杜鹃、黄毛海绵杜鹃、长叶川滇杜鹃、白背紫斑杜鹃6种。生于海拔3700～4200(4300)m冷杉等林下,主要伴生种有西康花楸(*Sorbus prattii* Koehne)、长尾槭(*Acer caudatum* Wall.)等。

3.3 高山灌丛型

该生境型的种类生长于海拔4200～5300m灌丛,对低温高湿有特殊的适应,仅1个亚型。有林芝杜鹃、雪层杜鹃、草莓花杜鹃和薄毛海绵杜鹃4种,常单种形成优势灌丛。

4 观赏评价

色季拉山杜鹃花除属高山灌丛型生境的种类,因花、叶小且要求低温高湿生境而难于驯化外,其它均有利用价值。鳞腺杜鹃、紫玉盘杜鹃、黄杯杜鹃在本世纪30年代已被国外引种[4]。依据花的特点,可将其观赏类群分为4类。

4.1 **香花类**　花具香味。有木兰杜鹃、大萼杜鹃、石峰杜鹃3种。花冠长5～7cm,白色,蜡

(C)1994-2023 China Academic Journal Electronic Publishing House. All rights reserved.　http://www.cnki.ne

质,外面带红晕,芳香。其花色淡雅,芬芳宜人,开发价值极高。

4.2 **团花类** 总状花序短缩呈团状的常绿杜鹃。主要有紫玉盘杜鹃、喉斑杜鹃、钟花杜鹃、光蕊杜鹃等种类。其特点为花团锦簇。花色通常为粉红至白色,也有淡紫色如钟花杜鹃等。此类多为大灌木或小乔木,可应用于园林绿化,喜肥,需要一定的空气湿度。

4.3 **雅致类** 植株矮小,花形花色多样,单花、少花或花序松散的种类,多为杜鹃亚属。主要有鳞腺杜鹃、散鳞杜鹃、三花杜鹃、柳条杜鹃、山育杜鹃、硬毛杜鹃、樱花杜鹃等。该类可作盆栽,喜光,是亟待开发的花木资源。

4.4 **黄花类** 本山区仅黄杯杜鹃1种。其花蕾期带红色,盛开后纯黄色,极为美丽。畏高温,需要一定的空气湿度。

参考文献

1 闵天禄、方瑞征、黄素华,1986,西藏植物志。科学出版社,北京,3:553～677。

2 沈渊如、沈荫椿,1985,杜鹃花。中国建筑工业出版社,北京,3～45。

3 郑维列,1992,西藏色季拉山报春花种质资源及其生境类型。园艺学报,19(3):261～266。

4 苏雪痕,1987,英国引种中国园林植物种质资源史实及应用概况。园艺学报,14(2):133～138

5 余清珠,1986,西藏产杜鹃花属一新种。植物研究,6(1):167～168。

A Study on the Germplasm Resources and Habitat of *Rhododendron* in Shergyla Mountain in Tibet

Zheng Weilie, Pan Gang, Xu Asheng and Luo Daqing

(*Tibet Plateau Ecology Res. Inst.*, *Xizang Nyingchi* 860000)

Abstract The rhododendron germplasm resources were investigated in shergyla mountain areas in Tibet. There existed 25 species or varieties. Among them 7 species distributed merely in the east slope area, 3 were in the west slope ares, 4 were in the top area and 11 were in both the east and the west area of the mountains. Based on the vegetation conditions, their habitat areas were divided into 3 types: forestedge shrubs type; forest type and alpine shrub type. The species number in forest type area was largest with 14, accounting for 48% of the total species (varieties). The 25 species (varieties) could be divided into fragrant flower group, ball inflorescence group, tasteful flower group and yellow flower group, according to the characterastics of their flowers.

Key words Tibet; Rhododendron; Germplasm; Habitat type

(C)1994-2023 China Academic Journal Electronic Publishing House. All rights reserved. http://www.cnki.ne
