# Supplementary material for: Metabolomics Based on UPLC-MS/MS Revealed the Metabolic Differences Among Four Species of Rhododendrons in Linzhi, Xizang
Source: Metabolites. 2026 Mar 30;16(4):226. doi: 10.3390/metabo16040226 (PMC13117825; doi:10.3390/metabo16040226)
Supplement: Supplementary file 1 [file metabolites-16-00226-s001.zip › Supplementary documents/Supplementary References/before13-now15.Zhu P, Shang Y, Yang L, et al (2013) Photosynthetic physiological characteristics of several alpine plants on the Qinghai–Xizang Plateau. .pdf]

886－892　　草业科学　　30卷06期
06/2013　　PRATACULTURAL SCIENCE　　Vol.30,No.06

青藏高原几种高山植物的光合生理特性

朱鹏锦[1,2],尚艳霞[2,3],杨莉[2,3],师生波[2],韩发[2]

(1.广西壮族自治区亚热带作物研究所,广西 南宁 530000;2.中国科学院西北高原生物研究所高原生物适应与进化重点实验室,青海 西宁 810001;3.中国科学院研究生院,北京 100049)

摘要:以14种高寒草甸植物为材料,研究分析植物叶片的光合色素含量和叶绿素荧光参数,以探究高寒草甸植物适应青藏高原极端环境的光合生理机制。结果表明,黄花鸢尾(*Iris pseudoacorus*)、美丽风毛菊(*Saussurea pulchra*)、鹅绒委陵菜(*Potentilla anserina*)和天蓝苜蓿(*Medicago lupulina*)叶片的单位面积叶绿素含量分别比黄帚橐吾(*Ligularia virgaurea*)高284.83%、200.56%、171.35%和148.31%,叶片PSⅡ有效光化学量子产量分别高79.99%、73.94%、68.37%和63.90%,叶片PSⅡ实际光化学效率也具有相同的变化趋势;而黄花鸢尾、美丽风毛菊、兰石草(*Lancea tibetica*)和麻花艽(*Gentiana straminea*)叶片的单位叶面积类胡萝卜素含量分别比天蓝苜蓿高393.93%、348.48%、260.61%和233.33%,其叶片的非光化学猝灭也显著高于天蓝苜蓿;黄帚橐吾、珠芽蓼(*Polygonum viviparnm*)和柔软紫菀(*Aster flaccidus*)叶片的叶绿素a/b值高于2.91,能适应较强光照,而天蓝苜蓿叶片的叶绿素a/b值小于2.5,常生长于植被下层,说明植物能够通过调整光合色素含量和构成而合理有效地利用光能以适应特殊的环境条件。

关键词:高寒草甸;植物;光合色素;叶绿素荧光参数

中图分类号:S812;Q945.11　　**文献标识码:**A　　**文章编号:**1001-0629(2013)06-0886-07

青藏高原是由冈瓦纳大陆向北分离的若干小板块与欧亚大陆在雅鲁藏布江缝合线处发生大陆板块的碰撞后,经过3次强烈构造运动引起的隆起和两次夷平作用而形成具有特殊的生态环境和独特的生物多样性的"世界屋脊"[1],位于亚洲大陆中南部,我国西部边陲。青藏高原由于独有的海拔和地貌特征,地面气温比同纬度平原地区低,气温的地域差异十分显著,而且具有明显的垂直梯度变化[2];全年无四季之分,仅冷暖之别,冷季月平均气温低达－15～－10℃,大部分暖季月平均气温低于10℃,是我国特有的高寒地域[3]。青藏高原地区受大陆性气候影响,绝大部分地区年降水量少,干旱与半干旱地区约占总面积的2/3,此外空气稀薄,太阳辐射强(比同纬度其它地区高50%～100%)紫外线强[4],形成寒冷、干燥、缺氧、太阳总辐射高和紫外线强的独特高原气候。

由于寒冷、干燥、缺氧和太阳总辐射高等高原气候的影响,青藏高原的植物生理生化特性、形态结构和生长发育均受到很大影响[5]。特别是处于寒冷和强近地面太阳紫外线环境胁迫中的植物,体内活性氧增加,损伤光合系统结构,迫使植物不断对高原气候产生的胁迫做出响应,以增强适应性谋求生存[6]。师生波等[7]根据青藏高原独有的海拔和强近地面太阳UV-B辐射,在青藏高原地区通过增补和滤除UV-B辐射模拟臭氧层损耗导致近地面UV-B辐射增加对植物的影响做了大量工作,研究表明,高原植物经长期自然选择与进化,在生理生化、形态结构和光合特性等方面形成了一定的适应机制;已有研究从分子水平上揭示了长白红景天(*Bhocliola angusta*)对低温胁迫的响应机理[8],也有研究从生理生化水平上解释青藏高原高山植物对强紫外辐射的适应机制[9-11],但是关于不同植物光合生理对青藏高原地区极端环境响应的研究甚少。因此,本研究拟通过分析植物叶片的叶绿素含量和叶绿素荧光参数特性,探究在极端环境下不同植物光合生理的适应性,

* 收稿日期:2012-12-12　接受日期:2013-02-08
基金项目:国家自然科学基金课题资助项目(30670307、30570270、30170154);国际科技合作重点项目计划(2002CB714006)
作者简介:朱鹏锦(1983-)男,广西博白人,研究实习员,硕士,植物生理生态学研究。E-mail:zhupengjin04@163.com
通信作者:师生波(1963-)男,青海西宁人,研究员,博士,主要从事高原生物生理生态适应性研究。E-mail:sbshi@nwipb.cas.cn

(C)1994-2023 China Academic Journal Electronic Publishing House. All rights reserved.　http://www.cnki.net

以期为评价高原气候胁迫下青藏高原地区高寒草甸不同植物的光合作用响应机制提供理论依据。

1 材料与方法

1.1 试验样地与材料 试验样地设立在中国科学院海北高寒草甸生态系统定位研究站(简称海北站)的综合观测场。研究站位于青藏高原东北部,即祁连山东段的冷龙岭南麓(37°37′ N,101°19′ E),山地平均海拔4 000 m,受明显的高原大陆性气候影响,年平均气温－1.7 ℃,年平均降水量为590.1 mm,降水主要集中在5－9月(占全年降水总量的80%)。年均实际日照时间为2 462.7 h,占理论可照时间的55.0%,年平均太阳辐射6 170 MJ·m^{-2},占太阳总辐射的60.1%。太阳辐射强烈,温度年差较小,日差较悬殊。近地面UV-B辐射高于同纬度其它地区。

植被类型为高寒矮嵩草(*Kobresia homilies*)草甸,主要由多年生草本植物组成,优势种为矮嵩草,常见伴生种见表1。

表1 高寒矮嵩草草甸常见伴生种

Table 1 Common companion species of *Kobresia homilies grassland* in alpine meadow

纲名 Class	编号 No.	科名 Family	属名 Genus	种名 Species
双子叶植物纲 Dicotyledoneae	1	蓼科 Polygonaceae	蓼属 *Polygonum*	珠芽蓼 *Polygonum viviparnm*
	2	蔷薇科 Rosaceae	委陵菜属 *Potentilla*	雪白委陵菜 *Potentilla acaulis*
	3	蔷薇科 Rosaceae	委陵菜属 *Potentilla*	鹅绒委陵菜 *Potentilla anserina*
	4	紫草科 Borginaceae	微孔草属 *Microula*	微孔草 *Microula sikkimensis*
	5	龙胆科 Gentianaceae	龙胆属 *Gentiana*	麻花艽 *Gentiana straminea*
	6	菊科 Compositae	紫菀属 *Aster*	柔软紫菀 *Aster flaccidus*
	7	菊科 Compositae	风毛菊属 *Saussurea*	美丽风毛菊 *Saussurea pulchra*
	8	菊科 Compositae	风毛菊属 *Saussurea*	瑞玲草 *Saussurea nigrescens*
	9	菊科 Compositae	橐吾属 *Ligularis*	箭叶橐吾 *Ligularia sagitta*
	10	菊科 Compositae	橐吾属 *Ligularis*	黄帚橐吾 *Ligularia virgaurea*
	11	豆科 Leguminosae	苜蓿属 *Medicago*	天蓝苜蓿 *Medicago lupulina*
	12	玄参科 Scrophulariaceae	兰石草属 *Lancea*	兰石草(肉果草)*Lancea tibetica*
单子叶植物纲 Monocotyledoneae	13	鸢尾科 Iridaceae	鸢尾属 *Iris*	黄花鸢尾 *Iris pseudoacorus*
	14	莎草科 Cyperaceae	苔草属 *Carex*	黑褐苔草 *Carex atrofusca*

1.2 测量方法

1.2.1 叶绿素荧光参数的测定 本研究在高寒草甸的草盛期(7、8月)进行,所观测的高山植物正值营养生长期。在连续两个月内选择天气晴朗的09:00－11:00,采用便携式脉冲荧光仪(FMS-2,英国)测定植物的叶绿素荧光参数。测定时,选择自顶部向地面的第3、4片叶(因为这两片叶子是完全展开且成熟的,再往下面的叶片可能会老化),并保证所选叶片向阳且受光角度相同,且测量部位一致及避开叶片中部主脉。

1.2.2 叶绿素和类胡萝卜素含量的测定 选取的叶片与测定叶绿素荧光参数的要求相同,避开主脉,用打孔器(直径0.7 cm)取18个叶圆片,混合均匀随机分成3组,每组6片,即每种植物3个重复,将叶圆片分别放入装有10 mL叶绿素提取液($V_{丙酮}$:$V_{乙醇}$:$V_{水}$＝45:45:10)的试瓶中,密封低温保存至叶圆片无色(约10 d)。用紫外分光光度计(UV-1601,日本)检测在663、645和440 nm波长处的吸光值。叶绿素含量的测定与计算参照Arnon[12]的方法,类胡萝卜素含量的测定与计算参照朱广廉[13]的测定方法,光合色素以单位叶片面积的含量表示。

1.3 数据分析 所测数据用SPSS 16.0软件进行统计分析与显著性检验。数据展示为重复±标准误,采用Originpro 8.5软件作图。

2 结果与分析

2.1 高寒草甸不同植物叶片光合色素含量的比较研究

2.1.1 高寒草甸14种植物叶片的叶绿素含量比较分析 植物叶片的叶绿色素在参与光合作用中涉及

(C)1994-2023 China Academic Journal Electronic Publishing House. All rights reserved. http://www.cnki.net

光能的吸收、传递和转化的过程，是客观反映植物利用光能的重要指标，也是作为判断植物光合生理能力、反映环境胁迫状况的重要指标，但是叶片的叶绿素含量变化与植物种类、环境变化密切相关。不同植物之间叶片的叶绿素含量具有一定差异，黄花鸢尾(13)、美丽风毛菊(7)、鹅绒委陵菜(3)和天蓝苜蓿(11)叶片的单位面积叶绿素含量比黄帚橐吾(10)分别高 284.83%、200.56%、171.35%和 148.31%(图 1)。

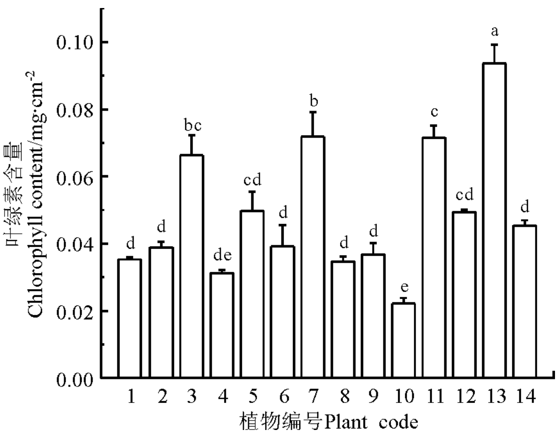

图 1　高寒草甸不同植物叶片的叶绿素含量比较

Fig. 1　Comparison of the chlorophyll content of different plant leaves at alpine meadow

注：不同小写字母表示差异显著($P<0.05$)。下同。

Note: Different lower case letters mean significant difference at 0.05 level. The same below.

2.1.2 高寒草甸 14 种植物叶片的叶绿素 a/b 的比较分析　植物叶片的叶绿素包括叶绿素 a 和叶绿素 b，叶绿素 a 主要与 PSⅠ和 PSⅡ的反应中心复合体结合，一部分位于捕光色素蛋白复合体(LHCⅡ)中，而叶绿素 b 主要分布在捕光色素蛋白复合体中，因此叶绿素 a/b 常作为一个评价植物对光照强度敏感性的指标[14]。本研究中有 10 种植物叶片的单位面积叶绿素 a/b 大于 2.91，其中黄帚橐吾(10)的比值最大，属阳生植物；黄花鸢尾(13)、鹅绒委陵菜(3)与黑褐苔草(14)叶片的比值在2.91和 2.5 之间；而天蓝苜蓿(11)最小，属阴生植物(图 2)。在层次聚类分析(图 3)中，高寒草甸 14 种植物叶片的单位面积叶绿素 a/b 可分为 3 类，与图 2 相一致。

2.1.3 高寒草甸 14 种植物叶片的类胡萝卜素含量比较分析　植物体内的类胡萝卜素与捕光色素蛋白复合体结合，既可以辅助吸收光能并传递给光系统反应中心的叶绿素 a，也可以在强近地面 UV-B 辐射和高太阳辐射的胁迫下促进非光化学猝灭耗散多余的光能，以实现光破坏防御的功能[15]，体现植物对过剩光能耗散的能力[16]。黄花鸢尾(13)、美丽风毛菊(7)、兰石草(12)和麻花艽(5)叶片的单位面积类胡萝卜素含量分别比天蓝苜蓿(11)高 393.93%、348.48%、260.61%和 233.33%(图 4)。

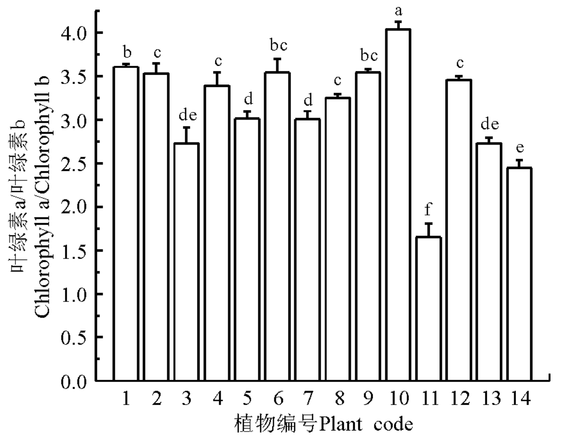

图 2　高寒草甸不同植物叶片的叶绿素 a/b 比值的比较

Fig. 2　Comparison of the Chla/Chlb of different plant leaves at alpine meadow

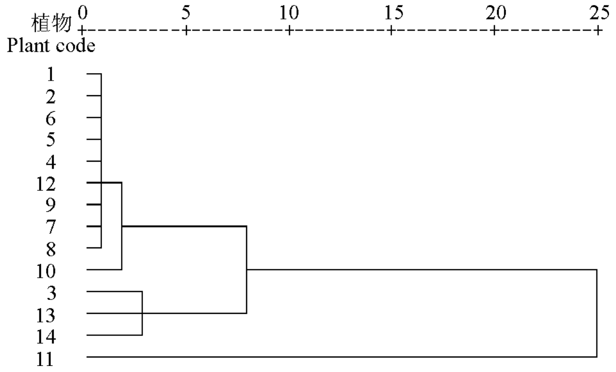

图 3　高寒草甸 14 种植物叶片的叶绿素 a/b 比值的聚类分析

Fig. 3　The dendrogram of hierarchical cluster analysis of the Chla/Chlb of 14 plant leaves at alpine meadow

2.2 高寒草甸植物叶片光适应下 PSII 有效光化学量子产量(Fv′/Fm′)和实际光化学效率(ΦpsⅡ)的比较分析　PSⅡ有效光化学量子产量(Fv′/Fm′)反映开放的 PSⅡ反应中心原初光能

(C)1994-2023 China Academic Journal Electronic Publishing House. All rights reserved.　http://www.cnki.net

捕获效率[17]。在连续两个月的测量中，晴天的数据表明植物叶片的PSⅡ有效光化学量子产量与其叶绿素含量具有相关性，即植物叶片的叶绿素含量高，其PSⅡ有效光化学量子产量也较高(图5)。实际光化学效率(ΦpsⅡ)，以(Fm′-Fs)/Fm′表示，代表线性电子传递的量子效率[17]。不同植物叶片的实际光化学效率的变化与其叶绿素含量及构成比例有关，与Fv′/Fm′的变化相似(图6)。

2.3 高寒草甸植物叶片光适应下光化学猝灭(qp)和非光化学猝灭(NPQ)的分析比较 光化学淬灭(qp)指的是PSⅡ天线色素吸收的光能用于光化学电子传递的份额，反映了光系统Ⅱ稳定性原初电子受体QA的还氧状态，是由于QA-重新氧化引起的；光化学淬灭系数(qp值)愈大，说明QA-重新氧化成为QA的量愈大，也就是光系统Ⅱ的电子传递活性愈大[18]。不同高寒草甸植物叶片的光适应下光化学猝灭(qp)与实际光化学效率(ΦpsⅡ)的变化相似。非光化学淬灭(NPQ)反映的是PSⅡ天线色素所吸收的光能中，不能用于光合电子传递而以热的形式耗散掉的部分；当PSⅡ反应中心天线色素吸收了过量光能时，如不能及时地耗散，将会引起光合机构失活或造成破坏，所以非光化学淬灭是

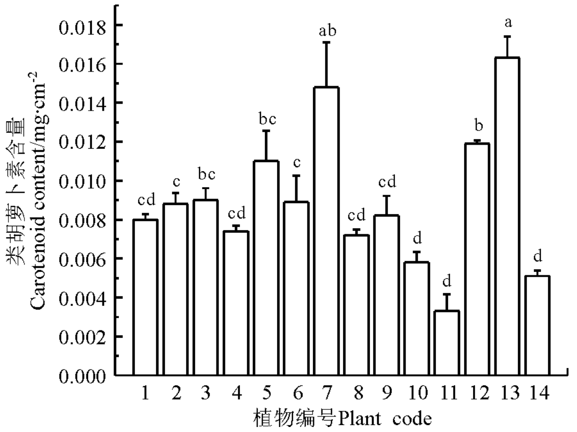

图4 高寒草甸不同植物叶片的类胡萝卜素含量比较

Fig. 4 Comparison of the carotenoid content of different plant leaves at alpine meadow

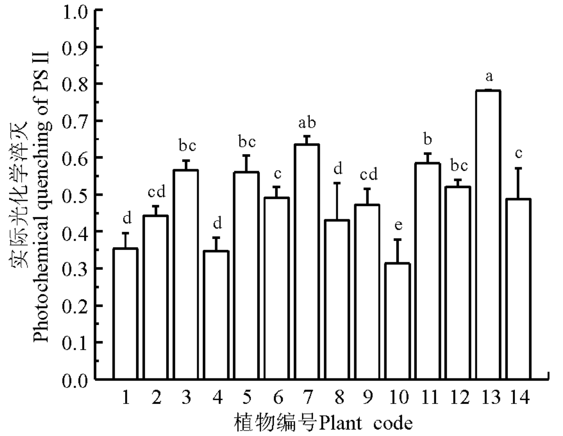

图6 高寒草甸14种植物叶片的实际光化学效率(ΦpsⅡ)的比较

Fig. 6 Comparison of the photochemical efficiency of PSⅡ in the light(ΦPSⅡ)of different plant leaves at alpine meadow

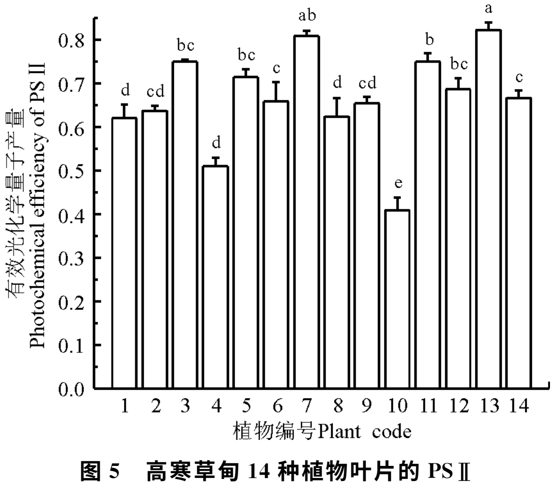

图5 高寒草甸14种植物叶片的PSⅡ有效光化学量子产量(Fv′/Fm′)的比较

Fig. 5 Comparison of the photochemical efficiency of PSⅡ in the light(Fv′/Fm′)of different plant leaves at alpine meadow

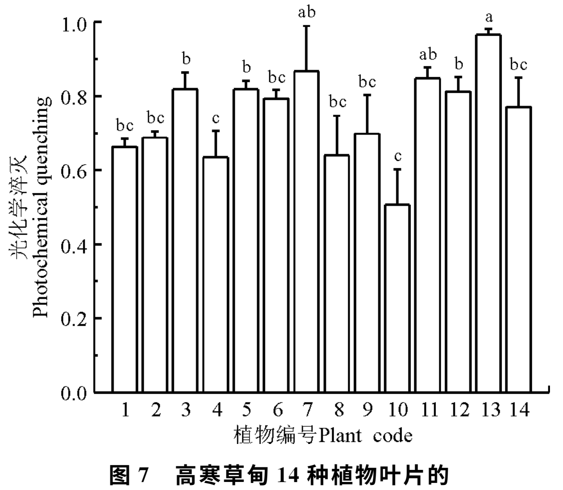

图7 高寒草甸14种植物叶片的光化学淬灭(qp)的比较

Fig. 7 Comparison of the photochemical quenching(qp)of different plant leaves at alpine meadow

(C)1994-2023 China Academic Journal Electronic Publishing House. All rights reserved. http://www.cnki.net

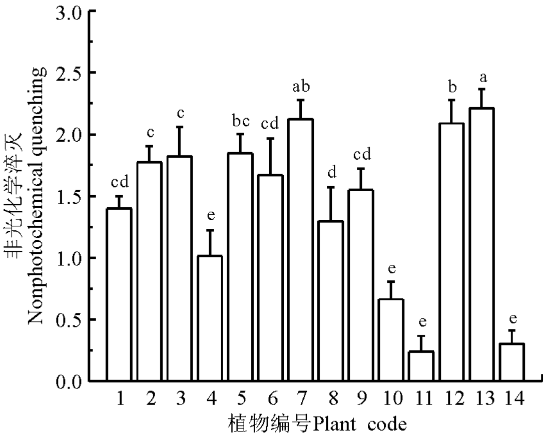

图 8 高寒草甸 14 种植物叶片的非光化学淬灭(NPQ)的比较

Fig. 8 Comparison of the Nonphotochemical quenching(NPQ) of different plant leaves at alpine meadow

一种自我保护机制，对光合机构起到一定的保护作用[17]（图 7）。不同高寒草甸植物叶片的非光化学猝灭的变化与其类胡萝卜素含量有关，即植物叶片类胡萝卜素含量高和构成比例大，则其非光化学猝灭较高(图 8)。

3 讨论

青藏高原生态环境独特，是研究植物对极端环境响应和适应性的理想平台。在极端环境的胁迫下，植物会通过改变叶片形态、调整叶片角度等行为来减少受光面积以及通过叶表蜡质层和微绒毛反射部分太阳辐射而有效避免强光和紫外线造成的损伤[5]。本研究发现，珠芽蓼、麻花艽、箭叶橐吾、兰石草和黄花鸢尾叶片均具有比较厚的蜡质层，而鹅绒委陵菜、微孔草、柔软紫菀和美丽风毛菊叶片则具有较多的微绒毛，这说明青藏高原地区的植物可通过改变其形态特征来减少对强光和紫外线的吸收，以适应环境。

本试验在对不同植物叶片单位面积叶绿素含量和叶绿素荧光参数特性的研究中发现光合色素含量的高低与组成比例不同，在一定程度上反映了实验中观测的高山植物光合生理对青藏高原地区高太阳辐射、强紫外线等独特环境的响应。如叶绿素含量较高的黄花鸢尾、美丽风毛菊、鹅绒委陵菜和天蓝苜蓿，其叶片的 PSⅡ 反应中心原初光能捕获效率与黄帚橐吾的相比也分别高79.99％、73.94％、68.37％和 63.90％，说明高山植物(黄花鸢尾、美丽风毛菊、鹅绒委陵菜和天蓝苜蓿)叶绿素含量增加能使叶片捕获和传递光能的效率增强。这与 Gabrielsen[21] 研究植物叶片不同叶绿素浓度对光合作用影响以及 McCall 和 Martin[22] 研究堪萨斯州东北部森林 3 种植物叶绿素浓度和光合作用的结果一致。在高寒草甸生态系统中天蓝苜蓿植株较矮、叶片薄且小，对强太阳辐射比较敏感，而且根据图 1 和图 2 能推出天蓝苜蓿单位面积叶绿素 b 含量多，表明天蓝苜蓿通过提高叶绿素 b 的含量增加捕光色素蛋白复合体的比例以获得足够的光能。Bailey 等[23] 研究拟南芥(*Arabidopsis thaliana*)对光照的响应的结果表明，叶绿素 b 合成加快和捕光色素蛋白复合体使捕光能力增强，本研究结果与其相似。叶绿素 a/b 是一个判断植物对光强适应性的常用参数[14,24]。天蓝苜蓿叶片的单位面积叶绿素 a/b 值最小(＜1.7)，而 10 种植物的比值均大于 2.91，其中黄帚橐吾比值最大。该结果与 Johnson[25] 通过对 19 种植物的研究发现阳生植物的叶绿素 a/b 为(2.91±0.08)，而阴生植物的叶绿素 a/b 为(2.59±0.11)的结论相似。

植物对吸收光能的分配和利用主要包括光化学反应转化光能、非光化学热能耗散以及叶绿素荧光形式耗散过剩光能。光化学反应和热耗散的变化会引起叶绿素荧光猝灭过程的相应变化，因此，采用叶绿素荧光动力学能够无损伤地快速反映逆境胁迫下植物的光合生理特性[18,26]。植物叶片的 PSⅡ 实际光化学效率代表线性电子传递的量子效率，常用来反映电子在 PSⅠ 和 PSⅡ 的传递情况，是荧光参数的重要组成部分。14 种植物中黄花鸢尾、美丽风毛菊、天蓝苜蓿和鹅绒委陵菜叶片的 PSⅡ 实际光化学效率比较高，这与其光合色素的构成有关，但目前色素组成比例与光能利用之间的机制仍不明确，有待结合飞秒分辨差异吸收光谱技术进行深入研究。叶绿素荧光猝灭有光化学猝灭和非光化学猝灭两种形式。植物叶片的类胡萝卜素含量与其非光化学猝灭具有相关性，说明类胡萝卜素能够促进非光化学猝灭；不同植物间光化学猝灭与 PSⅡ 实际光化学效率的变化趋势是相似的，因为光化学猝灭系数大小反映了光系统Ⅱ的电子传递活性大小。

(C)1994-2023 China Academic Journal Electronic Publishing House. All rights reserved. http://www.cnki.net

绿色植物的光合作用过程受外界环境变化的影响非常明显，其中光照强度、温度和 CO_2 浓度等环境因素对光合作用的影响最大。本研究是在自然条件下通过分析植物光合色素含量和构成比例以及叶绿素荧光参数，发现所观测的高山植物能调整其叶片光合色素含量和比例进行有效的光能利用和分配。据此认为，这可能是一种高山植物对极端环境的适应性机制。本研究进行光合生理的分析中存在一定的局限性：1）仅从植物光合色素含量和比例以及叶绿素荧光参数进行分析，不能够系统、全面地分析光合作用过程中色素的具体功能；2）高山植物由于植株低矮、叶片很小的形态特征要结合光合仪测定光合速率进行研究分析受到限制。嗣后研究重点后是在野外试验站进行以自然光为背景长期增补紫外线 UV-B 和滤除近地面紫外线 UV-B 实验，深入探讨高山植物对极端环境的光合生理适应机制。

4 结论

本研究发现在青藏高原高寒草甸生态系统中，所观测的高山植物不仅形态特征发生改变以适应环境，还通过调整其叶片光合色素的构成和含量来适应该地区高太阳辐射、强 UV-B 辐射和低温的环境。当植物的叶绿素 a 含量高而 PSⅡ 实际光化学效率受限制使得捕获的光能过剩时，捕光色素蛋白复合体中的类胡萝卜素则会进行热能耗散以减轻光氧化损伤；若植物的叶绿素 a 含量低且吸收的光能不能满足光合作用需求时，植物通过提高叶绿素 b 的含量使得捕光色素蛋白复合体增加，光能利用效率增强，以致在这极端环境下植物能有效地利用和耗散光能而谋求生存与繁殖。

参考文献

[1] 崔之久，李德文，伍永秋，等. 关于夷平面[J]. 科学通报，1998，43(17)：1794-1805.
[2] 孙鸿烈. 青藏高原研究的新进展[J]. 地球科学进展，1996，11(6)：536-542.
[3] 孙鸿烈，郑度. 青藏高原的综合考察与科学研究[A]. 中国科学技术协会. 科技进步与学科发展——“科学技术面向新世纪”学术年会论文集[C]. 北京：中国科学技术协会，1998：5.
[4] 莫申国，张百平，程维明，等. 青藏高原的主要环境效应[J]. 地理科学进展，2004，23(2)：88-96.
[5] 戴怡龄，安黎哲，陈拓，等. 寒区不同海拔橘黄罂粟叶片结构特征的比较研究[J]. 西北植物学报，2004，24(3)：495-503.
[6] 师生波，贲桂英，赵新全，等. 增强 UV-B 辐射对高山植物麻花艽净光合速率的影响[J]. 植物生态学报，2001，25(5)：520-524.
[7] 师生波，尚艳霞，朱鹏锦，等. 增补 UV-B 辐射对高山植物美丽风毛菊光合作用和光合色素的影响[J]. 草地学报，2010，18(5)：607-614.
[8] 颜廷芬，周福军，阎秀峰，等. 长白红景天天然种群遗传多样性及遗传分化[J]. 植物研究，1999(2)：69-74.
[9] 周党卫，朱文琰，腾中华，等. 不同海拔珠芽蓼抗氧化系统的研究[J]. 应用与环境生物学报，2003(5)：489-492.
[10] 韩发，周党卫，滕中华，等. 青藏高原不同海拔矮嵩草抗氧化系统的比较[J]. 西北植物学报，2003(9)：1491-1496.
[11] 汪晓峰，任红旭，孙国钧. 四裂红景天与长鳞红景天叶片中抗氧化系统随海拔梯度的变化[J]. 植物生态学报，2005(2)：331-337.
[12] Arnon D I. Copper enzymes in isolated chloroplasts. polyphenoloxidase in *Beta vulgaris*[J]. Plant Physiology，1949，24(1)：1.
[13] 朱广廉. 植物生理实验[M]. 北京：北京大学出版社，1990：4.
[14] 孙小玲，许岳飞，马鲁沂，等. 植株叶片的光合色素构成对遮阴的响应[J]. 植物生态学报，2010，34(8)：989-999.
[15] Matsubara S，Krause G H，Seltmann M，*et al*. Lutein epoxide cycle，light harvesting and photoprotection in species of the tropical tree genus *Inga*[J]. Plant，Cell and Environment，2008，31(4)：548-561.
[16] Tracewell C A，Vrettos J S，Bautista J A，*et al*. Carotenoid photooxidation in photosystem II[J]. Archives of Biochemistry and Biophysics，2001，385(1)：61-69.
[17] Bilger W，Björkman O. Role of the xanthophyll cycle in photoprotection elucidated by measurements of light-induced absorbance changes，fluorescence and photosynthesis in leaves of *Hedera canariensis*[J]. Photosynthesis Research，1990，25(3)：173-185.
[18] 王可玢，许春辉，赵福洪，等. 水分胁迫对小麦旗叶某些体内叶绿素 a 荧光参数的影响[J]. 生物物理学报，

(C)1994-2023 China Academic Journal Electronic Publishing House. All rights reserved. http://www.cnki.net

1997,13(2):273-278.

[19] Hofmann R W,Swinnye E,Bloor S J,*et al*. Responses of nine *Trifolium repens* L. populations to ultraviolet-B radiation:Differential flavonol glycoside accumulation and biomass production[J]. Annals of Botany, 2000,86(3):527-537.

[20] Sullivan J H,Gitz D C,Liu-Gitz L,*et al*. Coupling short-term changes in ambient UV-B levels with induction of UV-screening compounds[J]. Photochemistry and Photobiology,2007,83(4):863-870.

[21] Gabrielsen E K. Effects of different chlorophyll concenttrations on photosynthesis in foliage leaves[J]. Physiologia Plantarum,1948,1(1):5-37.

[22] Mccall K K,Martin C E. Chlorophyll concentrations and photosynthesis in 3 forest understory mosses in northeastern KANSAS[J]. Bryologist,1991,94(1):25-29.

[23] Bailey S,Walters R G,Jansson S,*et al*. Acclimation of *Arabidopsis thaliana* to the light environment: the existence of separate low light and high light responses[J]. Planta,2001,213(5):794-801.

[24] Murchie E H,Horton P. Contrasting patterns of photosynthetic acclimation to the light environment are dependent on the differential expression of the responses to altered irradiance and spectral quality[J]. Plant,Cell and Environment,1998,21(2):139-148.

[25] Johnson G,Scholes J,Horton P,*et al*. Relationships between carotenoid composition and growth habit in British plant species[J]. Plant,Cell and Environment, 1993,16(6):681-686.

[26] 张守仁.叶绿素荧光动力学参数的意义及讨论[J].植物学通报,1999,16(4):444-448.

Comparison of photosynthetic pigment contents and the chlorophyll fluorescence of characteristics of different plant leaves in alpine meadow of Tibet plateau

ZHU Peng-jin[1,2], SHANG Yan-xia[2,3], YANG Li[2,3], SHI Sheng-bo[2], HAN Fa[2]

(1. Guangxi Institute of Sub-tropical Crops, Nanning 530000, China;
2. Key Laboratory of Adaptation and Evolution of Plateau Biology, Northwest Institute of Plateau Biology, Chinese Academy of Sciences, Xining 810001, China;
3. Graduate University of Chinese Academy of Sciences, Beijing 100049, China)

Abstract: Tibetan Plateau is known as the "Roof of the World" with harsh,unique climatic characteristics and geographic conditions and cold, high solar radiation and strong ultraviolet light *et al*,and has profound influences on the morphology, photosynthetic pigments content and photosynthetic process of leaf of alpine plants. The plateau climate is important to photosynthetic pigments content, the constituent and photosynthesis of leaf, few studies have been focused on the relationship between photosynthetic pigments and photosynthesis of alpine plants in the third pole. In this research,14 plants of 9 species were selected and photosynthetic pigments content and the chlorophyll fluorescence of characteristics of leaf in the alpine meadow ecosystem were measured. The results showed that 1) To some extent,the chlorophyll content of alpine plants can reflect the photosynthetic capacity of leave; 2) The different composition proportion of photosynthetic pigments is closely related to photosynthetic physiology of plants.

Key words: alpine meadow; plants; photosynthetic pigment; chlorophyll fluorescence characteristic

Corresponding author:SHI Sheng-bo E-mail:sbshi@nwipb.cas.cn

(C)1994-2023 China Academic Journal Electronic Publishing House. All rights reserved. http://www.cnki.net
