# Supplementary material for: Metabolomics Based on UPLC-MS/MS Revealed the Metabolic Differences Among Four Species of Rhododendrons in Linzhi, Xizang
Source: Metabolites. 2026 Mar 30;16(4):226. doi: 10.3390/metabo16040226 (PMC13117825; doi:10.3390/metabo16040226)
Supplement: Supplementary file 1 [file metabolites-16-00226-s001.zip › Supplementary documents/Supplementary References/before14-now16.Liu T, Ji Y, Liu Y, et al (2021) Transcriptome-based analysis of the adaptation mechanisms of five plant species to high-altitude light conditions in Maidika Wetland, Xizang. .pdf]

DOI：10.11913/PSJ.2095-0837.2021.60632
刘泰龙，姬亚丽，刘怡萱，吴玄峰，陈飞飞，刘星. 基于转录组测序探讨西藏麦地卡湿地5种植物对高海拔光照的适应机制[J]. 植物科学学报，2021，39(6)：632-642
Liu TL，Ji YL，Liu YX，Wu XF，Chen FF，Liu X. Study on the adaptive mechanisms of five plants to high-altitude light based on transcriptome sequencing in Maidica wetland of Tibet[J]. *Plant Science Journal*，2021，39(6)：632-642

基于转录组测序探讨西藏麦地卡湿地5种植物对高海拔光照的适应机制

刘泰龙[1,2]，姬亚丽[1,2]，刘怡萱[1,2]，吴玄峰[1,2]，陈飞飞[1,2]，刘 星[1,2,3]*

（1. 西藏大学理学院，拉萨 850000；2. 西藏大学青藏高原生态与环境研究中心——极端环境生物适应与进化实验室，拉萨 850000；3. 武汉大学生命科学学院，武汉 430000）

摘　要：基于转录组测序技术对杉叶藻（*Hippuris vulgaris* L.）、云南黄芪（*Astragalus yunnanensis* Franch.）、甘肃马先蒿（*Pedicularis kansuensis* Maxim.）、马先蒿（*P. ikomai* Sasaki.）以及穗花马先蒿（*P. spicata* Pall.）5种麦地卡湿地常见植物的转录组数据进行分析，比较筛选影响麦地卡湿地植物光合作用的通路及基因，探讨高原植物在光合作用方面的适应性机制。结果显示，通过搜索GO和KEGG基因数据库，发现大多数基因被注释到细胞过程，且主要在代谢过程。在基因的GO富集分析结果中发现，较多基因富集在光系统Ⅰ、天线系统、光系统Ⅱ和光合作用；而KEGG富集分析结果表明，5种植物中大多数基因均显著富集在光合作用、光合作用-天线蛋白、卟啉和叶绿素代谢以及氧化磷酸化通路。推测麦地卡湿地植物可能通过卟啉和叶绿素代谢、氧化磷酸化等生物途径来抵御强光对自身照射的伤害，维持正常的生理活动。证明在高原极端环境条件影响下，麦地卡湿地生存的植物在光合作用中产生了适应性策略。

关键词：麦地卡湿地；转录组；光合作用；杉叶藻；云南黄芪；甘肃马先蒿；马先蒿；穗花马先蒿

中图分类号：Q943.2　　**文献标识码：**A　　**文章编号：**2095-0837(2021)06-0632-11

Study on the adaptive mechanisms of five plants to high-altitude light based on transcriptome sequencing in Maidica wetland of Tibet

Liu Tai-Long[1,2], Ji Ya-Li[1,2], Liu Yi-Xuan[1,2], Wu Xuan-Feng[1,2], Chen Fei-Fei[1,2], Liu Xing[1,2,3]*

（1. *College of Science*, *Tibet University*, Lhasa 850000, China；2. *Research Center for Ecology and Environment of Qinghai-Tibetan Plateau*, *Tibet University*, *Extreme Environmental Biological Adaptation and Evolution Laboratory*, Lhasa 850000, China；3. *College of Life Science*, *Wuhan University*, Wuhan 430000, China）

Abstract：Based on transcriptome sequencing technology, we analyzed the transcriptome data of five common plants from the Maidica wetland of Tibet, including *Hippuris vulgaris* L., *Astragalus yunnanensis* Franch., *Pedicularis kansuensis* Maxim., *P. ikomai* Sasaki., and *P. spicata* Pall. The pathways and genes affecting photosynthesis of plants in the Maidica wetland were compared and screened to explore the adaptive mechanisms of plateau plants regarding photosynthesis. Based on searching the Gene Ontology (GO) and Kyoto Encyclopedia for Genes and Genomes (KEGG) databases, most genes were annotated into cellular processes, especially metabolic processes. GO enrichment analysis of genes showed that more genes were enriched in photosystem Ⅰ, antenna system, photosystem Ⅱ,

收稿日期：2021-05-24，修回日期：2021-08-26。
基金项目：西藏麦地卡湿地生态站建设项目(00060352)。
This work was supported by a grant from the Construction Project of Maidica Wetland Ecological Station in Tibet (00060352).
作者简介：刘泰龙(1998-)，男，硕士研究生，研究方向为青藏高原生物多样性与分子进化(E-mail：1810464846@qq.com)。
* 通讯作者(Author for correspondence. E-mail：xingliu@whu.edu.cn)。
© Plant Science Journal http://www.plantscience.cn

and photosynthesis; KEGG enrichment analysis showed that most genes in the five plants were significantly enriched in pathways related to photosynthesis, photosynthesis-antenna protein, porphyrin and chlorophyll metabolism, and oxidative phosphorylation. It is speculated that plants in the Maidica wetland may resist damage from strong light and maintain normal physiological activities through porphyrin and chlorophyll metabolism, oxidative phosphorylation, and other biological pathways. These results indicate that under the influence of extreme environmental plateaus conditions, Maidica wetland plants use various adaptive strategies in photosynthesis.

Key words: Maidica wetland; Transcriptome; Photosynthesis; *Hippuris vulgaris*; *Astragalus yunnanensis*; *Pedicularis kansuensis*; *Pedicularis ikomai*; *Pedicularis spicata*

青藏高原作为世界上海拔最高、地域最为广阔的高原[1]，有着独特的高原气候条件，如太阳辐射强、日照时间长、气温低、昼夜温差大、气压低、氧气和二氧化碳含量少，湿度和降水量不均衡等[2]。这些特点对生长在青藏高原的植物，不仅影响其形态结构，而且对植物的生理代谢过程也会产生影响[3, 4]。麦地卡湿地自然保护区位于西藏自治区中部偏北的那曲地区嘉黎县北部，面积43 496 hm^2，平均海拔 4900 m，属于高原湖泊沼泽草甸湿地。该地一年四季不分明，夏秋为暖季，冬春为冷季，属于高原亚寒带半湿润气候区[5]。保护区内湿地类型多、湖泊数量多、水资源储量丰富，是众多水禽和其他生物的栖息繁殖地，也是研究高原生物遗传与物种保护的天然场所[6]。现有关麦地卡湿地的研究仅涉及保护区内环境保护和湿地种子植物资源多样性研究等，而关于麦地卡湿地植物响应环境胁迫方面的研究很少。

西藏大部分地区的总辐射较大且日照时间长[7, 8]，植物在生长期内可以获得充足的光能[9]。太阳光能作为植物进行光合作用、制造有机物质的唯一能量来源，直接影响植物的生长发育[10, 11]，对光合作用之外的许多生理过程，也都有明显影响[12–14]，因此研究高原植物在强光照强辐射下如何进行光合作用具有极其重要的意义。

研究表明，高原上的植物为了抵御高原极端的自然环境，进化出许多特殊的适应性性状[15]。例如，随着海拔升高，矮蒿草(*Kobresia humilis* (C. A. Mey. ex Trautv.) Sergievskaya)通过增强光合速率的方式在强光下能利用更多的光能[16]；生长于高海拔的珠芽蓼(*Polygonum viviparum* L.)[17]通过增加光能利用率来积极适应强辐射，缓解高山逆境导致的光抑制；麻花艽(*Gentiana straminea* Maxim.)[18]和冰川毛茛(*Ranunculus glacialis* L.)[19]通过较高的光呼吸强度积极适应强辐射。本文以典型分布在麦地卡湿地的 5 种不同植物为材料，包括：杉叶藻(*Hippuris vulgaris* L.)、云南黄芪(*Astragalus yunnanensis* Franch.)、甘肃马先蒿(*Pedicularis kansuensis* Maxim.)、马先蒿(*P. ikomai* Sasaki.)、穗花马先蒿(*P. spicata* Pall.)，采用 Illumina 高通量测序平台进行转录组测序，对 5 种植物的基因注释及光合作用中基因参与的主要生物学功能进行分析，探讨高原植物的光合作用适应机制。研究结果旨在为开展麦地卡湿地植物适应高原极端环境相关研究奠定基础。

1　材料与方法

1.1　实验材料的采集

麦地卡湿地 5 种植物杉叶藻(SYZ)、云南黄芪(YNHQ)、甘肃马先蒿(GSMXH)、马先蒿(MXH)和穗花马先蒿(SHMXH)采集于海拔 4691 m 的西藏麦地卡湿地-乌琼措(30°57′12″N，92°57′04″E)。利用 TPJ-22-G 型温度照度记录仪进行测量，记录采样时光照强度为 155 000 lx。采集部位为植物新鲜的叶片组织，用 RNAase-free 水清洗后分装在 RNAase-free 的 EP 管中，并保证每个植物装 3 管，满足 3 个生物学重复。然后将样品速冻在液氮中，储存在−80℃的超低温冰箱中备用。

1.2　RNA 提取和检测

采用 Trizol 法提取 5 种植物总 RNA，将质量检测合格的样品进行 cDNA 文库的构建；利用带有 Oligo (dT)的磁珠与 ployA 进行 A-T 碱基配对，从总 RNA 中分离出 mRNA；对富集得到的 mRNA 进行片段化；在逆转录酶作用下，加入 6 碱基随机引

© Plant Science Journal　http://www.plantscience.cn

物反转合成 cDNA；最后将反转合成的双链 cDNA 连接测序接头。

1.3 测序和功能注释

采用上海美吉生物医药科技有限公司 Illumina Hiseq 400 SBS Kit（300 cycles）二代测序平台完成转录组测序。测序产生的原始数据利用软件 SeqPrep（https://github.com/jstjohn/SeqPrep）和 Sickle（https://github.com/najoshi/sickle）去除接头序列、低质量序列、碱基信息不确定比例较高的序列及长度过短序列，转换为干净数据（Clean data）。同时计算 Clean data 的 Q20、Q30（分别计算 Phred 数值大于 20、30 的碱基占总体碱基的百分比）、GC 含量（计算碱基 G 和 C 的数量总和占总的碱基数量的百分比）（表 2）。然后使用Trinity[20] 软件进行 *de novo* 组装，通过聚类和拼接得到 Unigenes。Unigenes 的功能注释通过搜索 KEGG（https://www.kegg.jp/）和 GO（http://geneontology.org/）数据库进行。

1.4 功能富集分析

采用软件 Goatools（https://github.com/tanghaibao/GOatools）对给定基因集进行 GO 富集分析，当 *P*-value corrected ≤ 0.05，认为 GO 功能存在显著富集情况。对基因进行 KEGG 通路富集分析，同样满足 *P*-value corrected ≤ 0.05 的 KEGG 通路定义为在基因集中显著富集的 KEGG 通路。

2 结果与分析

2.1 转录组组装和注释

本研究发现，5 种植物各样本的 Q20 均大于 97%，Q30 均大于 94%，GC 含量在 43% ~ 49%（表 1）。对杉叶藻的 Clean data 进行从头组装，得到了 35 816 个单基因和 73 160 个转录本，单基因平均长度为 1245.30 bp，N50（按照长度将组装 unigene/transcript 从大到小排序，累加转录本的长度到总长度的一半时，对应转录本的长度）长度为 1731 bp；云南黄芪组装得到了 43 536 个单基因和 85 329 个转录本，单基因平均长度为 1188.04 bp，N50 长度为 1781 bp；甘肃马先蒿组装得到了 49 375 个单基因和 93 593 个转录本，单基因平均长度为 876.62 bp，N50 长度为 1155 bp；马先蒿组装得到了 31 781 个单基因和 58 245 个转录本，单基因平均长度为 1095.93 bp，N50 长度为 1504 bp；穗花马先蒿组装得到了47 790个单基因和 96 987 个转录本，单基因平均长度为 848.98 bp，N50 长度为 1167 bp。

利用 GO 数据库，对基因和基因产物按照其参与的 BP（Biological process，生物过程）、CC（Cellular component，细胞组分）及 MF（Molecular function，分子功能）3 个方面进行分类注释，大致了解某个物种的全部基因产物的功能分类情况（图 1）。GO 注释结果表明，杉叶藻的基因产物成

表 1 测序数据质控结果

Table 1 Sequencing data quality control results

物种 Species	样品 Sample	原始测序序列 Raw reads	高质量纯净序列 Clean reads	测序错误率 Error rate (%)	Q20 (%)	Q30 (%)	GC 含量 GC content (%)
杉叶藻 *Hippuris vulgaris* L.	SYZ-1	50168308	49674226	0.0247	98.19	94.45	44.19
	SYZ-2	48301152	47825536	0.0247	98.18	94.43	46.30
	SYZ-3	44172928	43791990	0.0250	98.07	94.15	45.95
云南黄芪 *Astragalus yunnanensis* Franch.	YNHQ_1	49521304	49175124	0.0244	98.31	94.72	43.56
	YNHQ_2	50974410	50622830	0.0245	98.27	94.61	43.40
	YNHQ_3	49545362	49157428	0.0250	98.07	94.10	43.36
甘肃马先蒿 *Pedicularis kansuensis* Maxim.	GSMXH_1	46678864	46229328	0.0250	98.04	94.17	47.85
	GSMXH_2	51804594	51296232	0.0248	98.11	94.30	48.16
	GSMXH_3	42547664	41704272	0.0248	98.11	94.32	48.50
马先蒿 *Pedicularis ikomai* Sasaki.	MXH_1	46726598	46280046	0.0247	98.17	94.45	48.36
	MXH_2	50127198	49665594	0.0247	98.15	94.42	48.88
	MXH_3	51961356	51480754	0.0247	98.14	94.39	48.46
穗花马先蒿 *Pedicularis spicata* Pall.	SHMXH_1	50798856	50037708	0.0250	98.05	94.16	47.99
	SHMXH_2	44916974	44409844	0.0245	98.24	94.64	48.87
	SHMXH_3	44331190	43925622	0.0264	97.51	92.71	48.50

© Plant Science Journal http://www.plantscience.cn

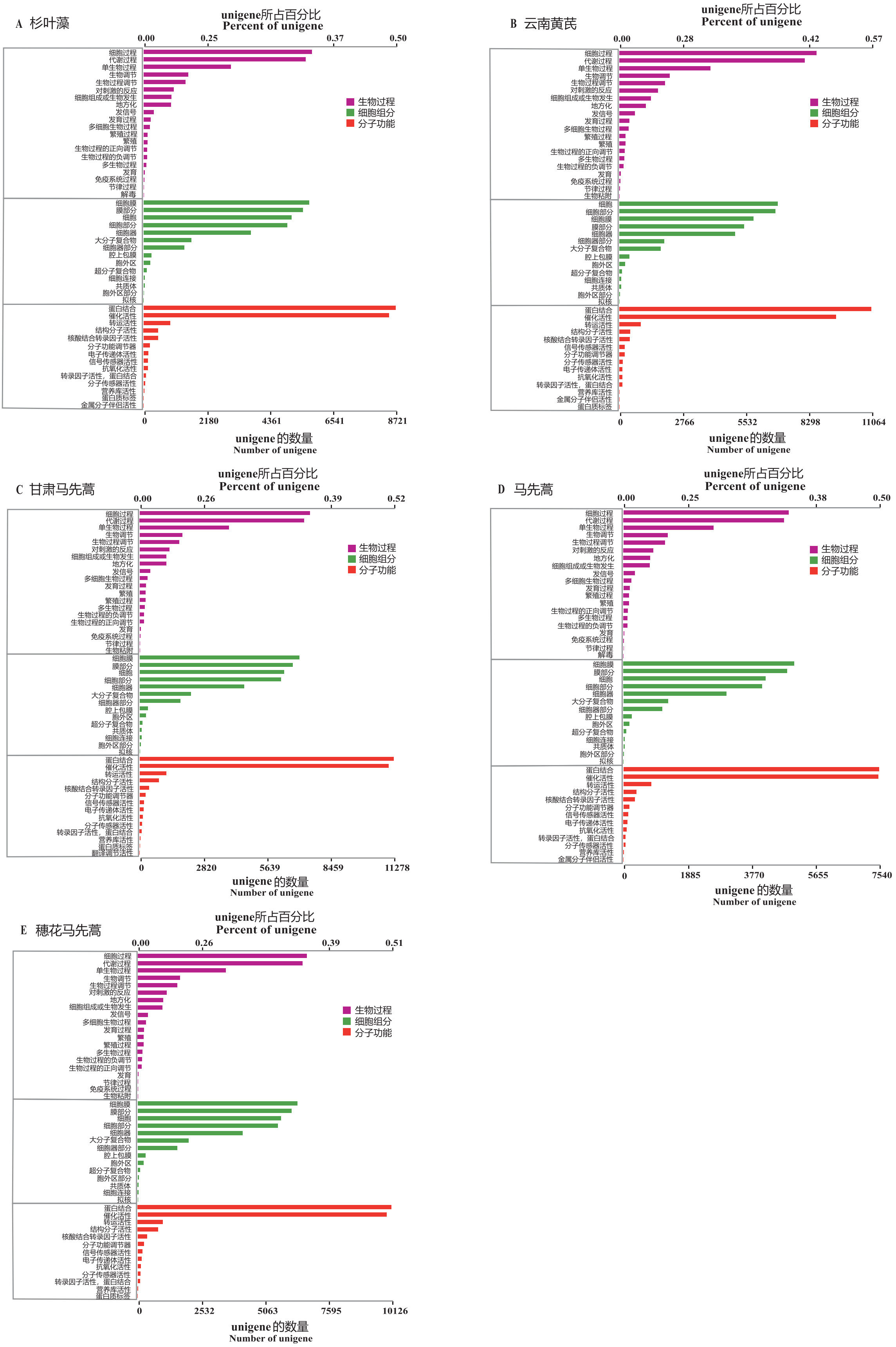

图 1　GO 分类统计

Fig. 1　GO classification statistics

© Plant Science Journal　http://www.plantscience.cn

功注释到48个功能组，其中生物过程(BP)注释到20个GO条目(GO term)，细胞组分(CC)注释到14个GO term，分子功能(MF)注释到14个GO term；云南黄芪的基因产物成功注释到48个功能组，其中BP注释到20个GO term，CC注释到14个GO term，MF注释到14个GO term；甘肃马先蒿的基因产物成功注释到48个功能组，其中BP注释到20个GO term，CC注释到14个GO term，MF注释到14个GO term；马先蒿的基因产物成功注释到47个功能组，其中BP注释到20个GO term，CC注释到14个GO term，MF注释到13个GO term；穗花马先蒿的基因产物成功注释到47个功能组，其中BP注释到20个GO term，CC注释到14个GO term，MF注释到13个GO term。在生物过程这一类中，5种植物的基因条目都主要注释在细胞过程、代谢过程、单一生物过程、生物调控、生物过程调节、响应刺激；在细胞成分这一类中，主要注释集中在膜、膜部分、细胞、细胞部分、细胞器、细胞器部分、大分子；在分子功能这一类中，主要注释集中在蛋白结合、催化活性、转运活性、结构分子活性方面。

KEGG数据库全称京都基因与基因项目组百科全书，可以将基因集中的基因按照参与的pathway通路或行使的功能分类(图2)，进一步理解基因在生物体中的功能[21]。通过对麦地卡5种植物unigene的KEGG代谢途径分类分析，发现在所有物种的所有代谢途径中，成功注释的基因多集中在代谢这一大类中。而在该大类中注释较多的子类有碳水化合物代谢、氨基酸代谢、能量代谢、脂质代谢；在遗传信息处理中注释较多的子类有基因翻译过程；在环境信息处理中注释较多的子类有信号转导；在细胞过程中注释较多的子类有分解和代谢；在生物体系统中注释较多的子类有环境适应。

2.2 功能富集分析

采用美吉云分析平台对注释到的基因进行关键词(光合作用)检索，并将检索后的基因建立基因集，进行后续GO和KEGG功能富集分析。基于TPM计算方法对基因进行功能富集，筛选FDR(*P*-value corrected) ≤ 0.05(即显著富集)，富集程度前10位的GO条目(图3)以及显著富集的KEGG通路(图4)。结果表明：麦地卡湿地植物杉叶藻光合作用相关的GO条目显著富集在光系统Ⅰ中光合作用和光捕获、光系统Ⅱ中光合电子传递、对弱光刺激的反应等；云南黄芪显著富集在光合作用、光捕获、光系统Ⅱ、光系统Ⅱ析氧复合体等；甘肃马先蒿显著富集在光系统Ⅱ析氧复合体、光系统Ⅱ中光合电子传递、膜外成分等；马先蒿显著富集在光合作用、光系统、光系统Ⅱ析氧复合体、光系统Ⅱ的光合作用电子传递等；穗花马先蒿显著富集在光合作用、光捕获、光系统Ⅱ活性细胞色素b6/f复合物中的电子传递、光系统Ⅰ反应中心、叶绿体类囊体膜等(图3)。KEGG富集分析显示：麦地卡湿地5种植物光合作用相关的unigene都显著富集(FDR ≤ 0.05)在光合作用、光合作用-天线蛋白、卟啉和叶绿素代谢以及氧化磷酸化通路(图4)。

3 讨论

青藏高原寒冷的气候、低氧条件和强烈的紫外线辐射环境对大多数生物来说都是非常极端的[22]，生存于这一地区的植物为适应这种极端环境必然进化产生了特殊的代谢适应性[23]，从而保证正常的生命活动。麦地卡湿地是青藏高原的一部分，同时作为天然湿地，也具有可以影响周围地区的小气候[24]。在高原极端环境和这种小气候的影响下，植物的生存环境变得更为复杂，生存在麦地卡湿地的植物可能会产生一些特殊的适应性机制，包括调节光合机构生理功能和基因表达，使其能够适应严酷多变的高原环境。

研究表明，植物体通过调节与光合作用相关基因的表达以适应高原强辐射环境[25]，本研究推测麦地卡湿地植物在光合作用通路方面的部分应答与适应高原强辐射环境有着直接联系。植物在面对强辐射这种极端环境时会产生特定的胁迫的适应性反应以及保护植物免受一种以上环境胁迫的响应[26]，这涉及不同调节水平之间复杂的相互作用[27]。当植物受到环境胁迫时，细胞膜最先感知外界胁迫刺激，然后通过膜上信号转导，最终将环境信号传递到下游信号通路，进而引起后续一系列分子和生理机制[28, 29]。通过对麦地卡湿地5种植物功能注释分析，我们发现有注释在膜、信号转导、代谢、生物调控、响应刺激和环境适应等条目。推测麦地卡湿地植物也存在利用膜感知和传递外界信号，直接或间接地感知应激启动信号转导途径，再通

© Plant Science Journal http://www.plantscience.cn

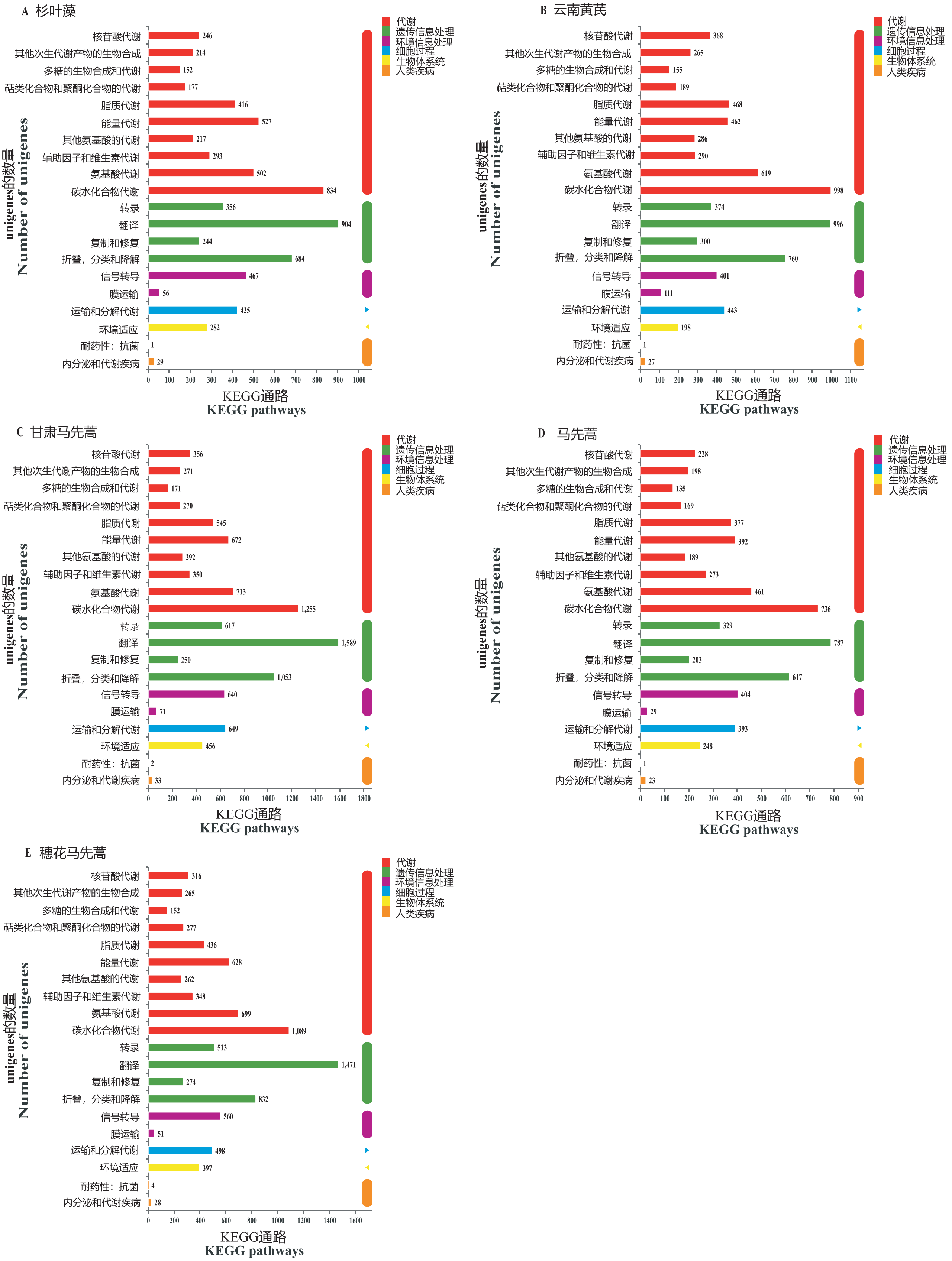

图 2　KEGG 分类统计

Fig. 2　KEGG classification statistics

© Plant Science Journal　http://www.plantscience.cn

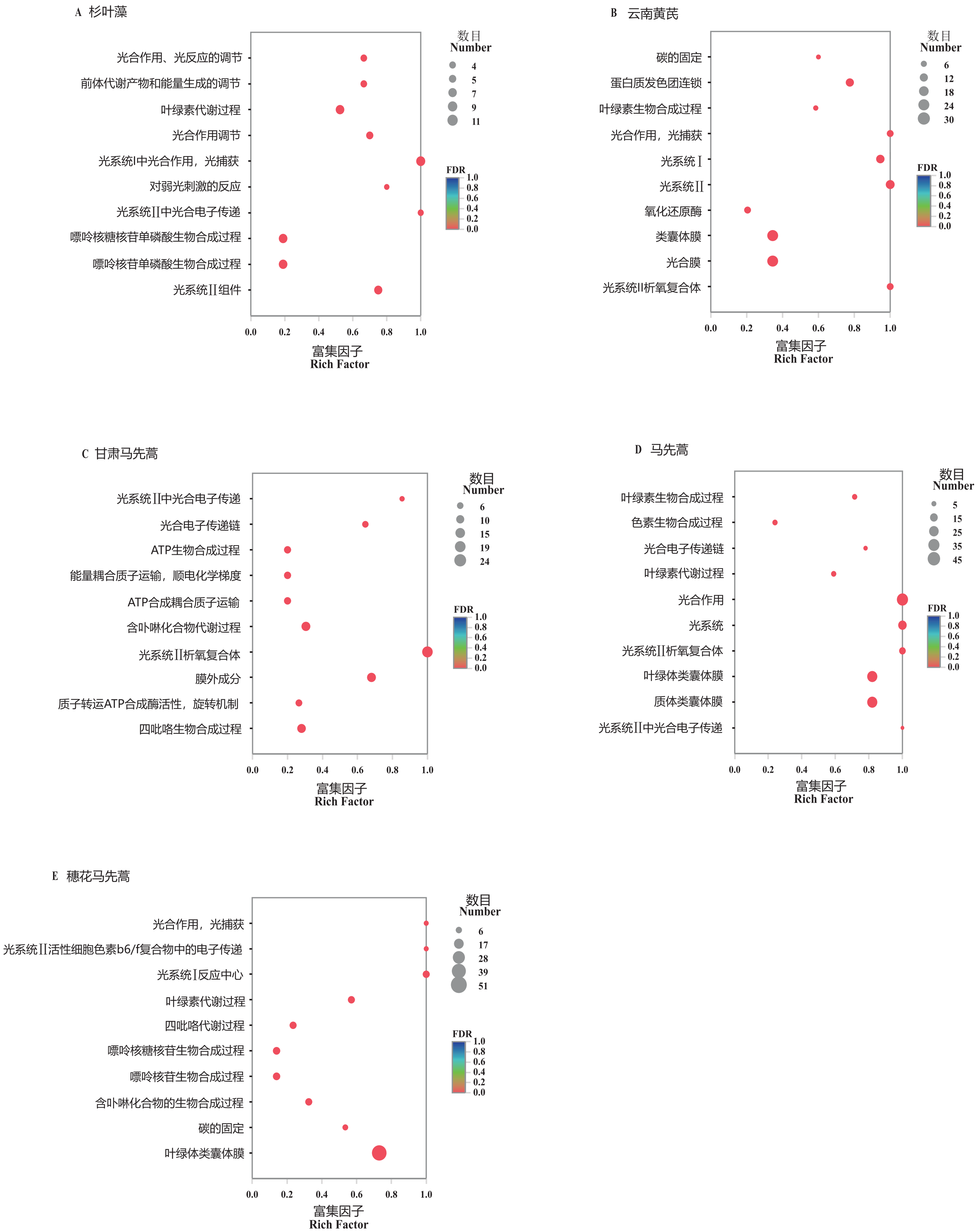

图 3 光合作用 GO 富集统计图

Fig. 3 GO enrichment statistics of photosynthesis

© Plant Science Journal http://www.plantscience.cn

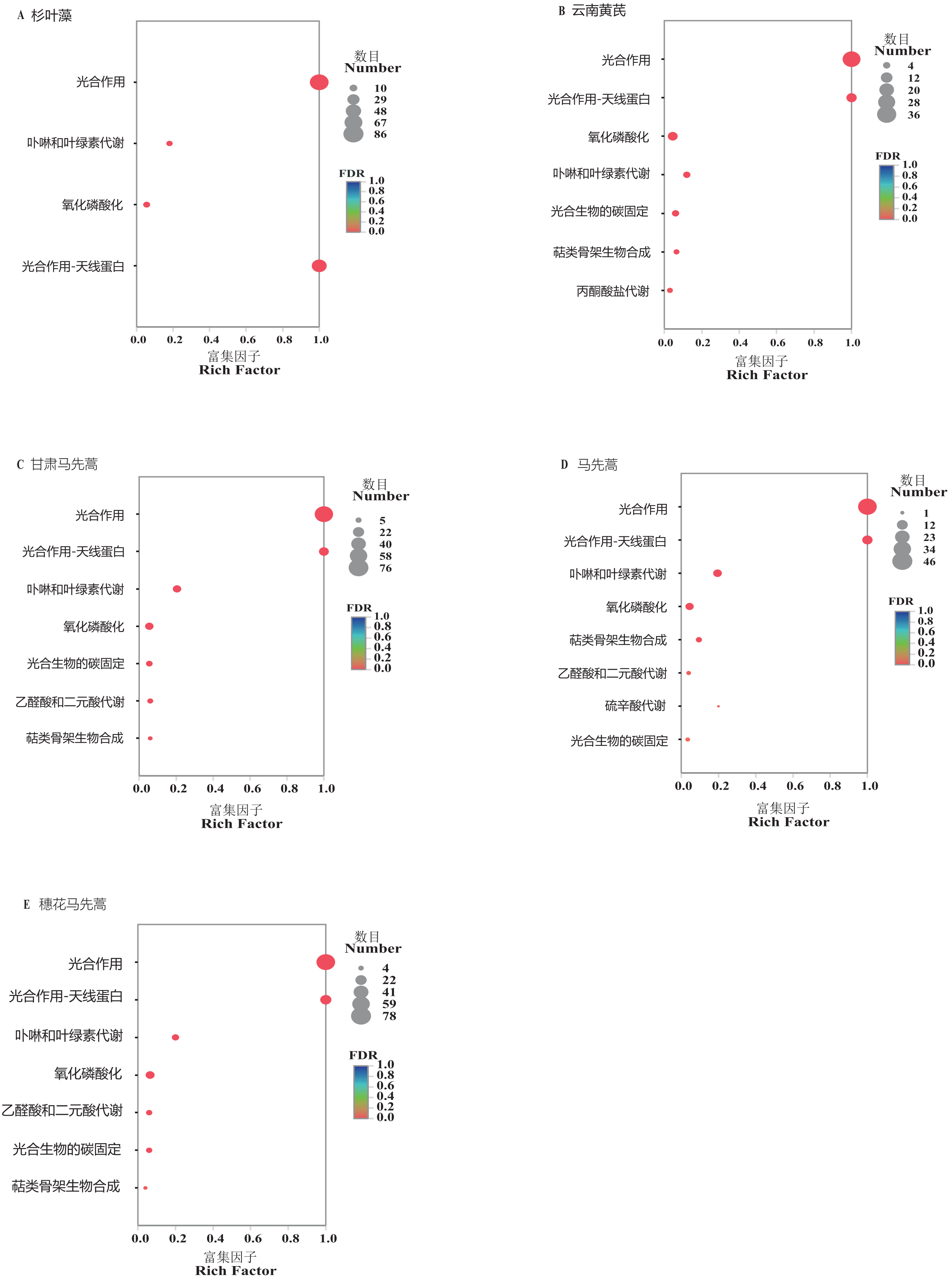

图 4　光合作用 KEGG 富集统计

Fig. 4　KEGG enrichment statistics of photosynthesis

© Plant Science Journal　http://www.plantscience.cn

过激活控制和协调适应所必需的生理和生化反应的信号转导级联来应对环境变化[26]，保护植物防御非生物胁迫。

本研究发现，麦地卡湿地的植物在高原极端环境条件下，植物光合作用产生了适应性策略。例如，光合作用是能量及物质的转化过程[30]，而天线蛋白能够捕获太阳能，并将其光能的95%输送至光合反应中心，经一系列反应将其储存，从而驱动光合作用[31]。同时天线蛋白作为一类捕获与传递光能的色素蛋白系统，具有光胁迫下能量耗散和光保护的功能[32]。本研究在麦地卡湿地植物光合作用有关的富集通路中发现显著富集天线蛋白，这可能是植物抵御环境胁迫的重要策略。研究发现，卟啉是叶绿素的主体，能影响叶绿素的合成[33]。叶绿素作为绿色植物叶绿体内参与光合作用的重要色素，在强光条件下它也是一种潜在的植物毒素[34]。麦地卡湿地的植物可能通过卟啉和叶绿素代谢的改变来应对外界胁迫，促进植物的高海拔耐受性。另外，能量的产生也是植物耐逆性的基础，而其中涉及能量代谢过程的氧化磷酸化途径是需氧细胞生命能量来源的主要方式[35, 36]，是生物产生ATP的主要途径。我们推测氧化磷酸化途径也可能为植物适应高原发挥了重要作用。

环境指标记录结果显示，采样时光照强度为155 000 lx，麦地卡湿地植物主要通过天线系统、卟啉和叶绿素代谢、氧化磷酸化途径，减轻强光照射对自身的伤害，维持自身生理活动的正常进行。本研究发现5种植物中与光合作用相关的基因*psaL*、*psaO*均有较高表达。*psaL*是光系统Ⅰ三聚化所必需的[37]，*psaO*是光合系统Ⅰ复体中核心亚基的一种，在两个光合系统之间平衡激发能方面起着重要作用[38]。光系统Ⅰ作为植物光合器官的膜蛋白复合体之一，负责收集光能并将其转化为化学能[39]。因此我们推测在高原强光照射下，植物在进行光合作用的过程中，光系统Ⅰ占主要部分。以往的研究表明，光系统Ⅰ和光系统Ⅱ是两种多蛋白复合物，其含有收获光子所必需的颜料，并利用光能催化产生高能化合物的主要光合作用的反应。这两种光系统都包含启动电子传递的反应中心，以及对于光捕获和光合作用活动调节很重要的外圈天线系统[40, 41]。在本研究中，5种植物同样在光系统Ⅰ、光系统Ⅱ等主要GO条目明显富集，证明在光合作用过程中光系统Ⅰ和光系统Ⅱ都明显活跃。

本研究利用转录组学的数据，探究了麦地卡湿地植物适应青藏高原环境的分子机制。青藏高原的植物为适应极端的生态环境，发生了分子机制的改变并产生代谢物用以抵抗多种非生物胁迫[42, 43]。麦地卡湿地作为青藏高原重要的环境资源之一，对该地区植物进行适应性进化研究，对于揭示该地区植物对高原气候的生态适应性机制具有一定的科学意义。

参考文献：

[1] Qiao Q, Wang Q, Han X, Guan Y, Sun H, *et al*. Transcriptome sequencing of *Crucihimalaya himalaica* (Brassicaceae) reveals how *Arabidopsis* close relative adapt to the Qinghai-Tibet Plateau[J]. *Sci Rep*, 2016, 6: 21729.

[2] Yoshikazu O, Akio F. Photoinhibition of elongation growth of roots in rice seedlings[J]. *Plant Cell Physiol*, 1967, 1: 141-150.

[3] Wang S, Guo L, He B, Lyu Y, Li T. The stability of Qinghai-Tibet Plateau ecosystem to climate change[J]. *Phys Chem Earth*, 2020, 115: 102827.

[4] Zhang T, Qiao Q, Novikova PY, Wang Q, Qiong L. Genome of *Crucihimalaya himalaica*, a close relative of *Arabidopsis*, shows ecological adaptation to high altitude[J]. *Proc Natl Acad Sci*, 2019, 116(14): 7137-7146.

[5] 拦继酒，罗建. 西藏麦地卡湿地自然保护区种子植物资源多样性[J]. 高原农业，2018，2(1)：7.
Lan JJ, Luo J. Diversity of seed plant resources in Maidica wetland natural reserve in Tibet[J]. *Journal of Plateau Agriculture*, 2018, 2(1): 7.

[6] 于萍萍. 西藏麦地卡湿地环境保护现状及其对策[J]. 农技服务，2016，33(1)：198.

[7] Gelsor N, Gelsor N, Wangmo T, Chen YC, Frette O, *et al*. Solar energy on the Tibetan Plateau: atmospheric influences[J]. *Sol Energy*, 2018, 173(10): 984-992.

[8] 徐宗学，巩同梁，赵芳芳. 近40年来西藏高原气候变化特征分析[J]. 亚热带资源与环境学报，2006，1(3)：24-32.
Xu ZX, Gong TL, Zhao FF. Analysis of climate change in Tibetan Plateau over the past 40 years[J]. *Journal of Subtropical Resources and Environment*, 2006, 1(3): 24-32.

[9] 刘志民，杨甲定. 青藏高原几个主要环境因子对植物的生理效应[J]. 中国沙漠，2000，20(3)：309-313.
Liu ZM, Yang JD. Effects of several environmental factors on plant physiology in Qinghai-Xizang Plateau[J]. *Journal of Desert Research*, 2000, 20(3): 309-313.

[10] Cui XY, Gu S, Wu J, Tang YH. Photosynthetic response to dynamic changes of light and air humidity in two moss

© Plant Science Journal http://www.plantscience.cn

species from the Tibetan Plateau[J]. *Ecol Res*, 2009, 24(3): 645-653.

[11] Khuong T. Investigation of the regulation of photosynthesis at the molecular level for improvement of plant growth and productivity under limiting light conditions[D]. Aix: Marseille University, 2013.

[12] Kurilik A, Mikluyt-Anova R, Dapkūnien S, Ilinskait S, Ukauskas A. *In vitro* cultivation of *Chrysantthemum* plantlets using light-emitting diodes[J]. *Cent Eur J Biol*, 2008, 3(2): 161.

[13] Kim H. Green-light supplementation for enhanced lettuce growth under red- and blue-light-emitting diodes[J]. *Hortscience*, 2004, 39(7): 1617.

[14] Hélène G, Claude VG, Nathalie B. Effects of blue light on the vertical colonization of space by white clover and their consequences for dry matter distribution[J]. *Ann Bot*, 1997, 80(5): 665-671.

[15] Sun H, Niu Y, Chen YS, Song B, Liu CQ, *et al*. Survival and reproduction of plant species in the Qinghai-Tibet Plateau[J]. *J Syst Evol*, 2014, 52(3): 378-396.

[16] 韩发，贲桂英，师生波. 青藏高原不同海拔矮嵩草抗逆性的比较研究[J]. 生态学报, 1998, 18(6): 654-659.
Han F, Ben GY, Shi SB. Comparative study on the resistance of *Kobresia humilis* grown at different altitudes in Qinghai-Xizang Plateau[J]. *Acta Ecologica Sinica*, 1998, 18(6): 654-659.

[17] Wei J, Yu H, Zhong ZP, Kuang TY, Ben GY. Comparison of photosynthetic adaptability between *Kobresia humilis* and *Polygonum viviparum* on Qinghai Plateau[J]. *Acta Botanica Sinica*, 2001, 43(5): 486-489.

[18] 师生波，李惠梅，王学英，岳向国，徐文华，陈桂琛. 青藏高原几种典型高山植物的光合特性比较[J]. 植物生态学报, 2006, 30(1): 40-46.
Shi SB, Li HM, Wang XY, Yu XG, Xu WH, Chen GC. Comparative studies of photosynthetic characteristics in typical alpine plants of the Qinghai-Tibet Plateau[J]. *Chinese Journal of Plant Ecology*, 2006, 30(1): 40-46.

[19] Streb P, Josse EM, Gallouët E, Baptist F, Kuntz M, Cornic G. Evidence for alternative electron sinks to photosynthetic carbon assimilation in the high mountain plant species *Ranunculus glacialis*[J]. *Plant Cell Environ*, 2010, 28(9): 1123-1135.

[20] Grabherr MG, Haas BJ, Yassour M, Levin JZ, Thompson DA, *et al*. Full-length transcriptome assembly from RNA-Seq data without a reference genome[J]. *Nat Biotechnol*, 2011, 29(7): 644-652.

[21] Ogata H, Goto S, Sato K, Fujibuchi W, Kanehisa M. KEGG: kyoto encyclopedia of genes and genomes[J]. *Nucleic Acids Res*, 1999, 27(1): 29-34.

[22] Cheviron ZA, Brumfield RT. Genomic insights into adaptation to high-altitude environments[J]. *Heredity*, 2012, 108(4): 354-361.

[23] 刘天猛. 青藏高原植物适应性进化和精油资源利用探究[D]. 拉萨：西藏大学, 2018.

[24] 李妍妍，王景升，税燕萍，陈歆，郑国强，等. 拉萨河源头麦地卡湿地景观格局及功能动态分析[J]. 生态学报, 2018, 38(24): 8700-8707.
Li YY, Wang JS, Shui YP, Chen X, Zheng GQ, *et al*. Analysis of landscape pattern and ecological service function of the Madica Wetland Reserve[J]. *Acta Ecologica Sinica*, 2018, 38(24): 8700-8707.

[25] Drop B, Webber-Birungi M, Yadav S, Filipowicz-Szymanska A, Fusetti F, *et al*. Light-harvesting complex Ⅱ (LHCⅡ) and its supramolecular organization in *Chlamydomonas reinhardtii*[J]. *BBA-Bioenergetics*, 2014, 1837(1): 63-72.

[26] Huang GT, Ma SL, Bai LP, Zhang L, Ma H, *et al*. Signal transduction during cold, salt, and drought stresses in plants[J]. *Mol Biol Rep*, 2012, 39(2): 969-987.

[27] Krasensky J, Jonak C. Drought, salt, and temperature stress-induced metabolic rearrangements and regulatory networks[J]. *J Exp Bot*, 2012, 63(4): 1593.

[28] Chrispeels MJ, Holuigue L, Latorre R, Luan S, Trewavas A. Signal transduction networks and the biology of plant cells[J]. *Biol Res*, 1999, 32(1): 35-60.

[29] Shimotohno A, Aki SS, Takahashi N, Umeda M. Regulation of the plant cell cycle in response to hormones and the environment[J]. *Annu Rev Plant Biol*, 2021, 72(1): 273-296.

[30] Meunier C. Energy from photobioreactors: Bioencapsulation of photosynthetically active molecules, organelles, and whole cells within biologically inert matrices[J]. *Pure Appl Chem*, 2008, 80(11): 2345-2376.

[31] Iwasaki Y, KomanoM, Takabe T. Molecular cloning of cDNA for a 17.5-kDa polypeptide, the *psaL* gene product, associated with cucumber photosystem Ⅰ[J]. *Biosci Biotech Bioch*, 2014, 59(9): 1758-1760.

[32] Ruban AV, Berera R, Ilioaia C, Stokkum IHMV, Kennis JTM, *et al*. Identification of a mechanism of photoprotective energy dissipation in higher plants[J]. *Nature*, 2007, 450(7169): 575-578.

[33] Neuberger A. The regulation of chlorophyll and porphyrin biosynthesis[J]. *Int J Biochem*, 1980, 12(5-6): 787-789.

[34] Hu G, Yalpani N, Briggs SP, Johal GS. A Porphyrin pathway impairment is responsible for the phenotype of a dominant disease Lesion Mimic Mutant of Maize[J]. *Plant Cell*, 1998, 10(7): 1095-1105.

[35] Liska AJ. Enhanced photosynthesis and redox energy production contribute to salinity tolerance in *Dunaliella* as revealed by homology-based proteomics[J]. *Plant Physiol*,

© Plant Science Journal　http://www.plantscience.cn

2004, 136(1): 2806-2817.

[36] Baris TZ, Blier PU, Pichaud N, Crawford DL, Oleksiak MF. Gene by environmental interactions affecting oxidative phosphorylation and thermal sensitivity[J]. *Am J Physiol-Reg I*, 2016, 311(1): 157-165.

[37] Chitnis VP, Chitnis PR. *PsaL* subunit is required for the formation of photosystem Ⅰ trimers in the cyanobacterium *Synechocystis* sp. PCC 6803[J]. *Febs Lett*, 1993, 336(2): 330-334.

[38] Jensen PE, Haldrup A, Zhang S, Scheller HV. The PSI-O subunit of plant photosystem Ⅰ is involved in balancing the excitation pressure between the two photosystems[J]. *J Biol Chem*, 2004, 279(23): 24212-24217.

[39] Kato K, Nagao R, Jiang TY, Ueno Y, Akita F. Structure of a cyanobacterial photosystem Ⅰ tetramer revealed by cryo-electron microscopy[J]. *Nat Commun*, 2019, 10(1): 4929.

[40] Horton P, Ruban AV, Walters RG. Regulation of light harvesting in green plants[J]. *Annu Rev Plant Phys*, 1996, 47(1): 655-684.

[41] Demmig-Adams B, AdamsIII WW, Mattoo AK. Photoprotection, photoinhibition, gene regulation, and environment[M]//Govindjee, ed. Advances in Photosynthesis and Respiration: Vol 21. Dordrecht: Springer, 2006.

[42] Yang Y, Zhou Z, Li Y, Lv Y, Yang D, *et al*. Uncovering the role of a positive selection site of wax ester synthase/diacylglycerol acyltransferase in two closely related *Stipa* species in wax ester synthesis under drought stress[J]. *J Exp Bot*, 2020, 71(14): 4159-4170.

[43] Ma L, Sun X, Kong X, Galvan JV, Li X, *et al*. Physiological, biochemical and proteomics analysis reveals the adaptation strategies of the alpine plant *Potentilla saundersiana* at altitude gradient of the Northwestern Tibetan Plateau[J]. *J Proteomics*, 2015, 112: 63-82.

(责任编辑:周 媛)

© Plant Science Journal http://www.plantscience.cn
