# Supplementary material for: Metabolomics Based on UPLC-MS/MS Revealed the Metabolic Differences Among Four Species of Rhododendrons in Linzhi, Xizang
Source: Metabolites. 2026 Mar 30;16(4):226. doi: 10.3390/metabo16040226 (PMC13117825; doi:10.3390/metabo16040226)
Supplement: Supplementary file 1 [file metabolites-16-00226-s001.zip › Supplementary documents/Supplementary References/before21-now23.Xie J, Zheng Y, Xu X, et al (2021) Inhibitory effect of myricetin on skin photoaging and NF-κB signaling pathway expression. .pdf]

●论　著

杨梅黄酮对皮肤光老化及 NF-κB 信号通路表达的抑制作用

谢璟　郑炎焱　徐晓敏　朱莉

【摘要】 目的　探讨杨梅黄酮(Myr)对小鼠皮肤和人皮肤角质形成(HaCaT)细胞光老化的抑制作用及其对核转录因子(NF-κB)信号通路表达的影响。方法　将 50 只小鼠按随机数字表法分为空白对照组(未经紫外线照射和 Myr处理)、模型对照组(背部涂抹0.9%氯化钠溶液 2 ml)、低剂量组(背部涂抹 5 mg/ml Myr 2 ml)、中剂量组(背部涂抹 20 mg/ml Myr 2 ml)、高剂量组(背部涂抹 50 mg/ml Myr 2 ml),每组各 10 只。除空白对照组外,其余各组均隔日用 Myr或 0.9%氯化钠溶液联合紫外线照射制备皮肤光老化模型。小鼠经末次(第 14 周)背部照射后,取照射区皮肤组织进行 HE 染色和胶原纤维染色,观察皮肤组织形态及真皮内胶原纤维的变化,检测皮肤抗氧化能力[超氧化物歧化酶(SOD)、总抗氧化力(T-AOC)和羟捕氨氧(HYP)]。HaCaT 细胞经 0、5、15、25、35、50 μM Myr处理后,用中波紫外线(UVB)照射建立细胞光老化模型,以未经 UVB 照射和药物处理细胞作为对照组,采用 MTT 方法检测各组细胞的活力,流式细胞术分析各组细胞的活性氧(ROS)水平。采用 RT-PCR 分别测定皮肤和细胞核转录因子 κB 抑制蛋白 α(IκBα)和环氧化酶(COX)-2 mRNA 表达水平,Western blot 检测细胞磷酸化(p)-IκBα、IκBα 和 COX-2 蛋白表达水平。结果　小鼠背部皮肤经紫外线照射后,均出现不同程度光老化现象。与模型对照组比较,中剂量组皮肤组织 SOD 活性、T-AOC 活性和 HYP 水平均升高,而 IκBα 和 COX-2 mRNA 表达水平均下调(均 $P<0.05$)。细胞光老化模型显示,35 μM 或 50 μM Myr预处理细胞可显著提高 UVB 照射后的细胞活性,并下调细胞的 ROS 水平(均 $P<0.05$)。50 μM Myr预处理细胞可显著抑制细胞 IκBα 和 COX-2 mRNA 表达,并下调 p-IκBα 和 COX-2 蛋白表达水平(均 $P<0.05$)。结论　Myr可提高皮肤和细胞抗氧化应激活性,降低氧化产物含量,抑制 NF-κB 信号通路活性,达到抗皮肤光老化作用。

【关键词】 杨梅黄酮　光老化　NF-κB 信号通路　氧化应激

Inhibitory effect of myricetin on skin photoaging and expression of NF-κB signaling pathway　*XIE Jing, ZHENG Yanyan, XU Xiaomin, ZHU Li. Department of Dermatology, Wenzhou People's Hospital, Wenzhou 325000,* China
Corresponding author: XIE Jing, E-mail: christian_xj@163.com

【Abstract】 Objective　To evaluate the inhibitory effect of Myricetin(Myr) on the photoaging in mice and its relation to NF-κB signaling pathway.　Methods　Fifty Kunming mice were randomly divided into blank control group, model control group, low dose group , medium dose group and high dose group with 10 mice in each group. The skin photoaging model was induced by UV radiation on alternate days in all groups except blank control group. Before modeling the mice were pre-treated with 2 ml saline(blank control and model control groups) or 2 ml Myr at concentration of 5 mg/ml(low dose group), 20 mg/ml(medium dose group)and 50 mg/ml(high dose group), respectively. After the last UV irradiation(the 14th week), the samples of dorsal skin tissue in the irradiated area were taken and HE and collagen fibers straining was performed. The changes of skin tissue morphology and collagen fibers in the dermis were observed, and the oxidative stress of skin was detected. Human keratinocytes (HaCaT) were treated with 0, 5, 15, 25, 35, 50 μM Myr in vitro and UVB irradiation was given to induce cell photoaging model. The viability of cells was detected by MTT method, and the ROS content in was analyzed by flow cytometry. The mRNA expression of IκBα and COX-2 were detected by RT-PCR, and the protein expression of p-IκBα, IκBα and COX-2 in cells were detected by Western blot.　Results　The dorsal skin of mice showed different degrees of photoaging after UV irradiation, and Myr had a al-

DOI:10.12056/j.issn.1006-2785.2021.43.14.2021-214

基金项目:温州市科技计划项目(Y20180662)

作者单位:325000　上海大学附属第三医院(温州市人民医院)皮肤科(谢璟、朱莉),神经内科(郑炎焱),妇产科学重点实验室(徐晓敏)

通信作者:谢璟,E-mail:christian_xj@163.com

(C)1994-2022 China Academic Journal Electronic Publishing House. All rights reserved.　http://www.cnki.net

leviating effect on the skin photoaging. Compared with model control group, the activity of SOD and T-AOC and the content of HYP in skin tissue of medium dose group were significantly increased, while the mRNA expression levels of IκBα and COX-2 were significantly decreased(all $P<0.05$). The cell photoaging model showed that 35 μM or 50 μM Myr significantly increased the cell activity after UVB radiation and down-regulated the ROS level(all $P<0.05$), 50 μM Myr significantly inhibited the mRNA and protein expression of IκBα and COX-2(all $P<0.05$). **Conclusion** Our data suggested that Myr may improve the anti-oxidative stress activity of skin cells, reduce the content of oxidation products, inhibit the activity of NF-κB signaling pathway, to exert the anti-skin photoaging effect.

【Key words】 Myricetin Photoaging NF-κB signaling pathway Oxidative stress

皮肤光老化是指长期日光中的紫外线照射皮肤后引起的皮肤老化现象[1]。引起光老化的紫外线主要是长波紫外线(ultraviolet A,UVA),中波紫外线(ultraviolet B,UVB),其中,大部分UVB被皮肤表皮吸收,小部分UVB可到达真皮浅层,而UVA可穿透表皮直达真皮深处,并在细胞中长期累积[2]。长期紫外线辐射可导致机体产生大量的活性氧(reactive oxygen species,ROS),由于ROS的产生,体内氧化应激通路被启动,进一步激活核转录因子κB(NF-kappaB,NF-κB)信号通路表达,引起炎症损伤、胶原蛋白降解,甚至诱发皮肤癌[3]。当前,开发具有抗氧化活性的药物来抵抗紫外线的辐射并预防和延缓皮肤老化,已成为皮肤医学领域的研究热点。杨梅黄酮(Myricetin,Myr)是杨梅树中提取的黄酮类抗氧化剂,可以与金属形成螯合物,减少金属离子对氧化作用的催化活性、抑制氧化酶活性、减少α-生育酚自由基等方式发挥抗氧化作用。此外,Myr还具有多种生物学活性,包括抗癌、防癌、抗菌、抗病毒、抗炎等[4]。但目前,Myr对紫外线诱导的皮肤光老化保护作用及潜在机制尚不清楚。本研究旨在通过建立小鼠和角质细胞光老化模型,初步探讨Myr对皮肤光老化及NF-κB通路表达的抑制作用,现报道如下。

1 材料和方法

1.1 实验动物和材料 选取雄性健康SPF级昆明小鼠50只,体重(30±2) g,鼠龄6~8周,购自温州医科大学实验动物中心。小鼠饲养在SPF级动物房内,每笼10只,自由摄食摄水,动物房室温控制在20~25 ℃,湿度60%,12 h明暗周期循环。本实验经温州医科大学动物伦理委员会批准。人皮肤角质形成(human keratinocytes,HaCaT)细胞购自中国典型培养物保藏中心;紫外线照射计和SS-03AB型紫外线光疗仪(UVA灯管6支≤20 J/cm^2;UVB灯管4支,≤5 J/cm^2)购自中国上海SIGMA高技术有限公司;细胞培养基和FBS均购自美国Gibco公司;Myr购自中国西安开来生物技术工程有限公司;核转录因子κB抑制蛋白(inhibitor of nuclear factor kappa-B kinase,IκB)-α和环氧化酶(cyclooxygenase,COX)-2抗体购自中国Proteintech公司;MTT试剂盒和BCA蛋白检测试剂盒购自中国碧云天生物技术有限公司;青霉素、链霉素和胰酶购自美国Sigma公司;抗氧化指标检测试剂盒购自中国Solarbio公司;引物序列由上海吉玛制药技术有限公司合成。

1.2 方法

1.2.1 小鼠光老化模型构建 光老化小鼠模型构建参考文献[5]:将光疗仪固定于暗室顶部,照射高度为30 cm;采用婴儿理发器剃除小鼠背部的绒毛,再用脱毛膏二次脱毛,然后采用随机数字表法分为5组,即空白对照组(未经紫外线照射和药物处理)、模型对照组(背部涂抹0.9%氯化钠溶液2 ml)、低剂量组(背部涂抹5 mg/ml Myr 2 ml)、中剂量组(背部涂抹20 mg/ml Myr 2 ml)、高剂量组(背部涂抹50 mg/ml Myr 2 ml),每组各10只;除空白对照组外,每组均隔日照射紫外线,照射前1 h涂抹药物或0.9%氯化钠溶液,30 min后用棉签拭去残留药物;照射强度为UVB 0.18 mW/cm^2,UVA 1.25 mW/cm^2,第1周至第7周每次照射时间为30 min,以后每周照射时间延长10 min,12周时每次照射时间为80 min,直至14周终止(建模期间UVA照射计量累计达148.5 J/cm^2,UVB照射计量累计达21.38 J/cm^2);定期观察各组小鼠背部皮肤皱纹生成、色素沉着、皮肤角化、毛细血管扩张等现象。

1.2.2 小鼠皮肤组织形态学检测 小鼠末次造模实验结束24 h后,采用戊巴比妥钠腹腔注射处死,取照射区部分皮肤组织,中性甲醛固定,乙醇系列脱水,二甲苯透明,常规石蜡包埋,制成切片后分别进行HE染色和胶原纤维染色。

1.2.3 HaCaT细胞光老化模型构建 采用含10% FBS,100 U/ml青霉素和100 μg/ml链霉素的DMEM培养基,在37 ℃、5%CO_2和20%O_2条件下培养HaCaT细胞。将HaCaT细胞按2.5×10^{6}个/ml接种在6孔板中,2 ml/孔,

(C)1994-2022 China Academic Journal Electronic Publishing House. All rights reserved. http://www.cnki.net

培养24 h后，用PBS清洗细胞2次后并向每孔加入2 ml PBS，分别采用0、15、20、25、30 mJ/cm^2 UVB（发射峰为313 nm）处理细胞，每种剂量设8个复孔。采用MTT试剂盒检测各组细胞活性（操作严格按照试剂盒说明书），确定细胞半数致死剂量。按5×10^4个/孔接种细胞，培养24 h后，分别采用含0、5、15、25、35、50 μM Myr的培养基（分别为0、5、15、25、35、50 μM Myr组）培养细胞24 h，每个Myr浓度组做8个复孔。用PBS清洗细胞2次后并加入1 ml PBS，采用半数致死UVB剂量处理细胞，并以未经紫外线照射和药物处理细胞作为对照组，检测各组细胞活性。

1.2.4 抗氧化能力检测 去除小鼠背部照射区皮肤皮下脂肪并称重，制成10%组织匀浆，3 000 r/min离心10 min，取上清液进行ROS水平测定。皮肤组织抗氧化能力检测指标包括超氧化物歧化酶（superoxide dismutasea，SOD）水平、总抗氧化力（total antioxidant capacity，T-AOC）和羟脯氨酸（hydroxyproline，HYP）水平，严格按照试剂盒说明书操作。采用流式细胞术测定细胞内ROS水平，即各组细胞经胰酶消化并用1ml PBS重悬后，添加ROS细胞渗透性指示剂CM-H2DCFDA至终浓度1 μM/ml，37 ℃暗室孵育30 min后，流式细胞仪检测活细胞内的ROS积聚情况。

1.2.5 IκBα和COX-2表达水平检测 采用Western blot法检测。使用细胞裂解液将细胞裂解后，取上清液，BCA法测定总蛋白浓度；30 μg蛋白上样，经15% SDS-PAGE电泳分离后将蛋白转移至PVDF膜上，5%脱脂奶粉封闭1.5 h；按照磷酸化（p）-IκBα、IκBα、COX-2和β-actin说明书中建议的使用浓度，4 ℃过夜孵育PVDF膜；漂洗PVDF膜，加入二抗，室温孵育1.5 h后漂洗，并进行显影，Quantity One软件扫描各条带的光密度并分析结果。

1.2.6 IκBα和COX-2 mRNA表达水平检测 提取细胞和组织RNA后，逆转录合成cDNA，以cDNA为模板，qRT-PCR检测样本中的COX-2表达量。IκBα上游引物：5'-TGAGGACCAGCAGTGTCTTG-3'，下游引物：5'-CATCGTTGATCACAAGTCGG-3'。COX-2上游引物：5'-TAAGTGCGATTGTACCCGGAC-3'，下游引物：5'-TTTGTAGCCATAGTCAGCATTGT-3'。

1.3 统计学处理 采用GraphPad Prism 8.0统计软件。计量资料以$\bar{x}\pm s$表示，多组间比较采用单因素方差分析，两两比较采用Dunnett-*t*检验。$P<0.05$为差异有统计学意义。

2 结果

2.1 紫外线照射后小鼠皮肤外观及组织形态 空白对照组小鼠背部皮肤细腻光滑，皮肤色泽正常，富有弹性；皮肤结构、层次清晰，毛囊汗腺等完整可见，胶原纤维呈波浪状紧密排列，整齐有序，分布均匀，胶原束的走向大多与皮面平行。其余各组经紫外线照射8周后，均出现不同程度的皮肤粗糙增厚，纹理加深加宽，鳞屑增加，皮肤皱褶明显，毛细血管扩张现象。模型对照组表皮不规则增厚，伴有角化过度，真皮胶原纤维排列紊乱，胶原束散开，弹力纤维增多、扭曲，以毛囊周围增多较明显；不同剂量的Myr处理后，表皮厚度不一，与模型对照组比较，真皮胶原纤维变性程度轻，排列呈现不同程度的紊乱，弹力纤维有所减少。其中，以中剂量组光老化反应和组织病变程度最轻，皮肤稍粗糙增厚，表面有少许鳞屑，皮肤可见浅皱褶，毛细血管轻微扩张。组织病理显示表皮不规则增厚，伴有角化过度，真皮胶原纤维排列较紊乱，胶原束部分散开。见图1（插页）。

2.2 紫外线照射后各组小鼠皮肤抗氧化能力及IκBα和COX-2 mRNA表达水平比较 见表1。

由表1可见，经紫外线照射后，各组小鼠皮肤组织抗氧化指标（SOD、T-AOC和HYP）及NF-κB信号通路

表1 紫外线照射后各组小鼠皮肤抗氧化能力及IκBα mRNA和COX-2 mRNA表达水平比较

组别	*n*	SOD（U/mg）	T-AOC（U/g）	HYP（μg/g）	IκBα mRNA	COX-2 mRNA
低剂量组	10	44.94±12.95	6.00±2.57*	494.20±164.30	1.49±0.53*	15.04±11.84*
中剂量组	10	68.00±18.16*△	8.28±1.55△	563.90±109.50△	0.37±0.19*△	7.33±6.10△
高剂量组	10	46.68±11.83	5.38±0.57*	483.80±80.79	1.22±0.13*	7.61±3.55△
空白对照组	10	50.74±17.64	10.04±3.73	561.80±249.50	0.17±0.12	0.88±0.68
模型对照组	10	31.26±11.00	4.45±1.68	328. 50±83.01	1.63±0.44	19.87±13.72
*F*值		8.16	10.00	3.67	10.19	7.22
*P*值		<0.05	<0.05	<0.05	<0.05	<0.05

注：SOD为超氧化物歧化酶；T-AOC为总抗氧化力；HYP为羟脯氨酸；IκBα为核因子κB抑制蛋白α；COX-2为环氧化酶2；与模型对照组比较，*$P<0.05$；与空白对照组比较，△$P<0.05$

(C)1994-2022 China Academic Journal Electronic Publishing House. All rights reserved. http://www.cnki.net

相关指标(IκBα mRNA、COX-2 mRNA)差异均有统计学意义(均 $P<0.05$)。与模型对照组比较，低剂量组和高剂量组 T-AOC 活性升高，中剂量组 SOD 活性升高，低剂量组 COX-2 mRNA 表达水平降低(均 $P<0.05$)；与空白对照组比较，中剂量组 SOD 活性、T-AOC 活性和 HYP 水平均升高，而 IκBα mRNA 表达水平降低（均 $P<0.05$）。

2.3 紫外线照射后各组小鼠 HaCaT 细胞活性和 ROS 水平比较 见图 2。

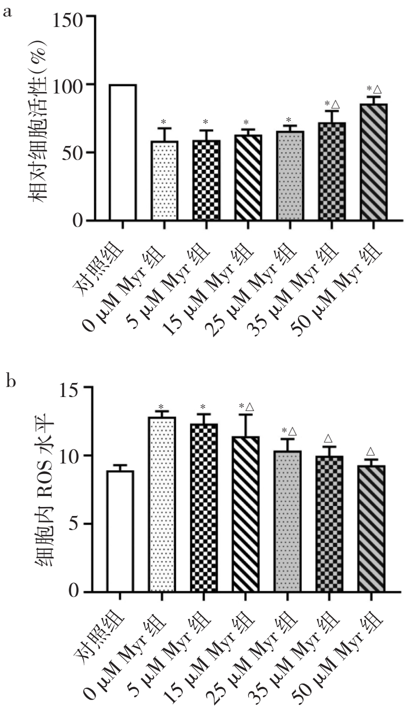

图2 紫外线照射后各组小鼠人角质形成(HaCaT)细胞活性和活性氧(ROS)水平比较(a:各组小鼠 HaCaT 细胞活性比较；b:各组小鼠细胞内 ROS 积聚水平比较；与对照组比较，$^*P<0.05$；与 0 μM Myr组比较，$^△P<0.05$)

由图 2 可见，随着 UVB 剂量的增加，HaCaT 细胞的活性逐渐降低，半数致死剂量约为 20 mJ/cm^2，其细胞活力为无 UVB 照射时的(58.60±9.07)%。在 20 mJ/cm^2 UVB 照射条件下，HaCaT 细胞的活力随着 Myr 处理浓度升高而上升，当 Myr 浓度达到 35、50 μM 时，细胞活性分别为对照组的(72.20±8.26)%和(85.80±4.97)%，与 0 μM Myr 组比较，差异均有统计学意义(均 $P<0.05$)。UVB 照射后，细胞内 ROS 水平均不同程度升高，但 Myr 可抑制 ROS 上调，与对照组比较，当 Myr 浓度为 35、50 μM时，组间 ROS 水平差异无统计学意义($P>0.05$)。

2.4 紫外线照射后各组小鼠 HaCaT 细胞 IκBα 和COX-2 表达水平比较 见表 2、图 3。

由表 2、图 3 可见，经紫外线照射后，与对照组比较，0 μM Myr 组 p-IκBα、IκBα 蛋白、IκBα mRNA、COX-2 蛋白及 COX-2 mRNA 表达水平均升高(均 $P<0.05$)，50 μM Myr 组 p-IκBα 蛋白、IκBα mRNA、COX-2 蛋白及 COX-2 mRNA 表达水平均降低(均 $P<0.05$)。

3 讨论

高达 80%~90%的皮肤老化是由于环境和外来生物制剂(外部老化)造成的，而长期紫外线照射是皮肤老化的主要原因[6-7]。紫外线照射皮肤数小时后，炎性细胞在皮肤表皮和真皮局部浸润，并产生大量的 ROS。ROS 细胞内聚积可以破坏细胞膜结构、引起 DNA 突变和蛋白变性，进而影响细胞结构并导致功能障碍[8]。而长期由 ROS 诱导的氧化应激反应不仅会导致机体氧化损伤，还将通过 DNA 损伤和激活癌基因的方式促进基底细胞癌、鳞状细胞癌、黑色素瘤等疾病发生[9]。寻找一种安全、高效的抗氧化剂，对于预防和延缓皮肤光老化有着重要意义。

Myr 又称 3,5,7,3',4',5'-六羟基黄酮，是一种天然安全的黄酮类化合物，相较于其他同类化合物，具有更强的抗菌、抗病毒、抗炎、抗氧化活性[10-11]。现有的 Myr 研究主要集中于肿瘤[12]、神经退行性病变[13]、心血管疾病[14]等领域，在皮肤光老化的防治方面研究仍较为缺乏。本文结果显示，Myr 可有效抑制紫外线对小鼠皮肤的组织学病变和胶原纤维变性，并上调胶原蛋白特征性氨基酸 HYP 水平。ROS 在皮肤聚集将持续消耗皮肤抗氧化物

表2 紫外线照射后各组小鼠 HaCaT 细胞 IκBα 和 COX-2 表达水平比较

组别	n	p-IκBα 蛋白	IκBα 蛋白	IκBα mRNA	COX-2 蛋白	COX-2 mRNA
0 μM Myr组	3	3.67±0.86*	1.17±0.10	2.75±0.27*	5.30±0.76*	10.47±2.01*
50 μM Myr 组	3	1.30±0.46$^△$	1.04±0.10	1.55±0.34$^△$	1.50±0.36$^△$	3.83±1.82$^△$
对照组	3	1	1	1	1	1
F值		20.14	3.65	38.50	71.10	28.92
P值		<0.05	>0.05	<0.05	<0.05	<0.05

注：IκBα 为核因子 κB 抑制蛋白 α；COX-2 为环氧化酶 2；HaCaT 细胞为人皮肤角质形成细胞；与对照组比较，$^*P<0.05$；与 0 μM Myr组比较，$^△P<0.05$

(C)1994-2022 China Academic Journal Electronic Publishing House. All rights reserved. http://www.cnki.net

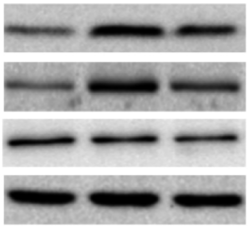

图3 加入杨梅黄酮(Myr)紫外线照射后皮肤磷酸化核转录因子κB抑制蛋白(p-IκBα)、核转录因子κB抑制蛋白(IκBα)和环氧化酶(COX)-2蛋白表达的电泳图

质,其中SOD是体内主要的抗氧化酶,其作用主要是清除超氧化物自由基,而T-AOC代表了机体清除ROS的能力[15-16]。本研究通过对不同剂量Myr处理后的皮肤组织检测发现,Myr不同程度提高组织SOD和T-AOC活性。其中,相比模型对照组,20 mg/ml Myr可显著提高小鼠皮肤抗氧化能力。角质形成细胞是皮肤抵抗紫外线的首要屏障,在光老化的发生和发展中起重要作用。本文通过角质形成细胞光老化模型也证实,Myr可有效抑制UVB对HaCaT细胞的杀伤作用和ROS细胞内聚集。提示Myr可通过提高皮肤抗氧化的能力来延缓皮肤光老化。

当细胞受到外界的刺激使其会发生反应,首先通过细胞表面的一些受体或者蛋白将细胞外信息传递到细胞内,随后细胞根据传递到细胞内部的信息,细胞内部一系列的蛋白分子与生理功能被激活,称这些蛋白分子构成的网络为信号通路。紫外线照射后NF-κB信号通路被激活是晒伤引起炎症反应的第一步。COX-2是NF-κB的一个下游靶点,因此,笔者推测Myr可能是通过NF-κB信号通路来发挥抑制紫外线辐射对人角质细胞的损伤作用。为了验证这一假设,本研究将HaCaT细胞用20 mJ/cm^{2}紫外线照射,然后用Myr处理细胞,之后收集细胞蛋白,用Western blot检测细胞p-IκBα和IκBα蛋白表达。结果发现,紫外线照射后细胞内的p-IκBα水平表达显著上调,IκBα未产生明显变化,当采用50 μM的Myr处理细胞,发现p-IκBα表达被抑制,IκBα仍未产生显著的变化。因此,笔者得出结论Myr可能是通过NF-κB信号通路来发挥抑制紫外线照射对细胞的损伤作用。多种信号通路都可通过降解IκBα的方式活化NF-κB,主要是使IκBα的丝氨酸残基发生磷酸化。活化后的NF-κB可以入核与DNA结合发挥其转录调控功能。IκBα首先是在IκBα激酶的作用下发生丝氨酸残基磷酸化,接下来IκBα在E3泛素化酶复合体催化下进行泛素化而被蛋白酶降解。活化后的NF-κB参与机体多种调控机制,主要是发挥转录活性功能,诱导下游基因的转录。经典的NF-κB信号通路主要由p50/p65介导,在未激活状态下,p50/p65与抑制因子IκBα结合形成蛋白复合体存在于细胞质中,当蛋白复合体受ROS、TNF、IL-1等刺激,将引起IκBα磷酸化并与p50/p65解离,进一步诱导p50/p65入核,启动下游炎症相关蛋白COX-2的表达[17-18]。本研究进一步检测Myr对小鼠和细胞IκBα和COX-2的作用表明,Myr可有效抑制紫外线对皮肤组织和细胞的IκBα和COX-2的上调作用,下调IκBα磷酸化水平,提示Myr可通过NF-κB信号通路抑制紫外线照射的损伤作用。

综上所述,本研究通过动物和细胞实验证实,Myr可提高皮肤抗氧化能力,通过调控NF-κB信号通路发挥抗炎活性,具有抑制紫外线诱导的皮肤光老化作用。

4 参考文献

[1] Wang M, Charareh P, Lei X, et al. Autophagy: multiple mechanisms to protect skin from ultraviolet radiation-driven photoaging[J]. Oxid Med Cell Longev, 2019:8135985. DOI:10.1155/2019/8135985.

[2] Petruk G, Del Giudice R, Rigano MM, et al. Antioxidants from plants protect against skin photoaging[J]. Oxid Med Cell Longev, 2018, 2018:1454936. DOI:10.1155/2018/1454936.

[3] Wang ML, Zhong QY, Lin BQ, et al. Andrographolide sodium bisulfate attenuates UV induced photo-damage by activating the keap1/Nrf2 pathway and downregulating the NF-κB pathway in HaCaT keratinocytes[J]. Int J Mol Med, 2020, 45(2):343-352. DOI:10.3892/ijmm.2019.4415.

[4] Song X, Tan L, Wang M, et al. Myricetin: A review of the most recent research[J]. Biomed Pharmacother, 2021, 134:111017. DOI:10.1016/j.biopha.2020.111017.

[5] 李张军, 牛新武, 肖生祥, 等. 小鼠皮肤光老化动物模型建立方法的改良[J]. 西安交通大学学报(医学版), 2016, 37(1):144-147. DOI:10.7652/jdyxb201601028.

[6] Lephart ED. Skin aging and oxidative stress: Equol's anti-aging effects via biochemical and molecular mechanisms[J]. Ageing Research Reviews, 2016, 31:36-54. DOI:10.1016/j.arr.2016.08.001.

[7] Lephart ED. Equol's anti-aging effects protect against environmental assaults by increasing skin antioxidant defense and ECM proteins while decreasing oxidative stress and inflammation[J]. Cosmetics, 2018, 5(1):16. DOI:10.3390/cosmetics5010016.

[8] Mohania D, Chandel S, Kumar P, et al. Ultraviolet radiations: skin defense-damage mechanism[J]. Adv Exp Med Biol, 2017, 996:71-87. DOI:10.1007/978-3-319-56017-5_7.

[9] Raimondi S, Suppa M, Gandini S. Melanoma epidemiology and sun exposure[J]. Acta Derm Venereol, 2020, 100(11):adv00136. DOI:10.2340/00015555-3491.

[10] Venturelli S, Burkard M, Biendl M, et al. Prenylated chalcones and flavonoids for the prevention and treatment of cancer[J].

(C)1994-2022 China Academic Journal Electronic Publishing House. All rights reserved. http://www.cnki.net

Nutrition, 2016, 32(11- 12):1171- 1178. DOI:10.1016/j.nut.2016.03.020.
[11] Jiang M, Zhu ML, Wang L, et al. Anti- tumor effects and associated molecular mechanisms of myricetin[J]. Biomed Pharmacother, 2019, 120:109506. DOI:10.1016/j.biopha.2019.109506.
[12] Afroze N, Pramodh S, Hussain A, et al. A review on myricetin as a potential therapeutic candidate for cancer prevention [J]. Biotech, 2020, 10(5):211. DOI:10.1007/s13205- 020- 02207- 3.
[13] Joshi V, Mishra R, Upadhyay A, et al. Polyphenolic flavonoid (myricetin) upregulated proteasomal degradation mechanisms: Eliminates neurodegenerative proteins aggregation[J]. J Cell Physiol, 2019, 234(11):20900- 20914. DOI:10.1002/jcp.28695.
[14] Wang L, Wu H, Yang F, et al. The protective effects of myricetin against cardiovascular disease[J]. J Nutr Sci Vitaminol (Tokyo), 2019, 65(6):470- 476. DOI:10.3177/jnsv.65.470.
[15] Li M, Lin XF, Lu J, et al. Hesperidin ameliorates UV radiation- induced skin damage by abrogation of oxidative stress and inflammatory in HaCaT cells[J]. J Photochem Photobiol B, 2016, 165:240- 245. DOI:10.1016/j.jphotobiol.2016.10.037.
[16] 雷波. 芦荟多糖对光老化大鼠皮肤中SOD、GSH- Px、Hyp、CAT、MDA水平的影响[J]. 宜春学院学报, 2020, 42(12):73- 75. DOI:10.3969/j.issn.1671- 380X.2020.12.016.
[17] Dolcet X, Llobet D, Pallares J, et al. NF- kB in development and progression of human cancer[J]. Virchows Arch, 2005, 446(5):475- 482. DOI:10.1007/s00428- 005- 1264- 9.
[18] Khan MA, Khan MJ. Nano- gold displayed anti- inflammatory property via NF- κB pathways by suppressing COX- 2 activity [J]. Artif Cells Nanomed Biotechnol, 2018, 46(sup1):1149- 1158. DOI:10.1080/21691401.2018.1446968.

（收稿日期:2021−04−22）
（本文编辑:马雯娜）

（上接第1482页）

sepsis. Correlation to CD64 and CD14 antigen expression [J]. Clin Exp Immunol, 2008, 154(1):87- 97. DOI:10.1111/j.1365- 2249.2008.03737.x.
[17] Li S, Huang X, Chen Z, et al. Neutrophil CD64 expression as a biomarker in the early diagnosis of bacterial infection: a meta- analysis[J]. Int J Infect Dis, 2013, 17(1):e12- 23. DOI:10.1016/j.ijid.2012.07.017.
[18] Wunschel SC, Jarman KH, Petersen CE, et al. Bacterial analysis by MALDI- TOF mass spectrometry: an inter- laboratory comparison[J]. Journal of the American Society for Mass Spectrometry, 2005, 16(4):456- 462. DOI:10.1016/j.jasms.2004.12.004.
[19] Long Y, Zhang Y, Gong Y, et al. Diagnosis of Sepsis with Cell- free DNA by Next- Generation Sequencing Technology in ICU Patients[J]. Archives of medical research, 2016, 47(5):365- 371. DOI:10.1016/j.arcmed.2016.08.004.
[20] Ferrer R, Martin- Loeches I, Phillips G, et al. Empiric antibiotic treatment reduces mortality in severe sepsis and septic shock from the first hour: results from a guideline- based performance improvement program[J]. Crit Care Med, 2014, 42(8):1749- 1755. DOI:10.1097/CCM.0000000000000330.
[21] Rivers E, Nguyen B, Havstad S, et al. Early goal- directed therapy in the treatment of severe sepsis and septic shock[J]. N Engl J Med, 2001, 345(19):1368- 1377. DOI:10.1056/NEJMoa010307.
[22] Rhodes A, Evans LE, Alhazzani W, et al. Surviving sepsis campaign: International guidelines for management of sepsis and septic shock: 2016[J]. Intensive Care Medicine, 2017, 43(3):304- 377. DOI:10.1007/s00134- 017- 4683- 6.
[23] Malbrain M, Van Regenmortel N, Saugel B, et al. Principles of fluid management and stewardship in septic shock: it is time to consider the four D's and the four phases of fluid therapy[J]. Annals of Intensive Care, 2018, 8(1):66. DOI:10.1186/s13613- 018- 0402- x.
[24] Stenson EK, Cvijanovich NZ, Allen GL, et al. Hyperchloremia is associated with acute kidney injury in pediatric patients with septic shock[J]. Intensive Care Medicine, 2018, 44(11):2004- 2005. DOI:10.1007/s00134- 018- 5368- 5.
[25] Yessayan L, Neyra JA, Canepa- Escaro F, et al. Effect of hyperchloremia on acute kidney injury in critically ill septic patients: a retrospective cohort study[J]. BMC Nephrol, 2017, 18(1):346. DOI:10.1186/s12882- 017- 0750- z.
[26] Rochwerg B, Alhazzani W, Gibson A, et al. Fluid type and the use of renal replacement therapy in sepsis: a systematic review and network meta- analysis[J]. Intensive Care Medicine, 2015, 41(9):1561- 1571. DOI:10.1007/s00134- 015- 3794- 1.
[27] Finfer S, Bellomo R, Boyce N, et al. A comparison of albumin and saline for fluid resuscitation in the intensive care unit[J]. N Engl J Med, 2004, 350(22):2247- 2256. DOI:10.1056/NEJMoa040232.
[28] Caironi P, Tognoni G, Masson S, et al. Albumin replacement in patients with severe sepsis or septic shock[J]. N Engl J Med, 2014, 370(15):1412- 1421. DOI:10.1056/NEJMoa1305727.
[29] Liu ZM, Chen J, Kou Q, et al. Terlipressin versus norepinephrine as infusion in patients with septic shock: a multicentre, randomised, double- blinded trial[J]. Intensive care medicine, 2018, 44(11):1816- 1825. DOI:10.1007/s00134- 018- 5267- 9.
[30] Minneci PC, Deans KJ, Eichacker PQ, et al. The effects of steroids during sepsis depend on dose and severity of illness: an updated meta- analysis[J]. Clin Microbiol Infect, 2009,15(4):308- 318. DOI:10.1111/j.1469- 0691.2009.02752.x.
[31] Kalil AC, Sun J. Low- dose steroids for septic shock and severe sepsis: the use of Bayesian statistics to resolve clinical trial controversies[J]. Intensive Care Medicine, 2011, 37(3):420- 429. DOI:10.1007/s00134- 010- 2121- 0.

（本文由浙江省医学会推荐）
（收稿日期:2020−10−21）
（本文编辑:严玮雯）

(C)1994-2022 China Academic Journal Electronic Publishing House. All rights reserved. http://www.cnki.net

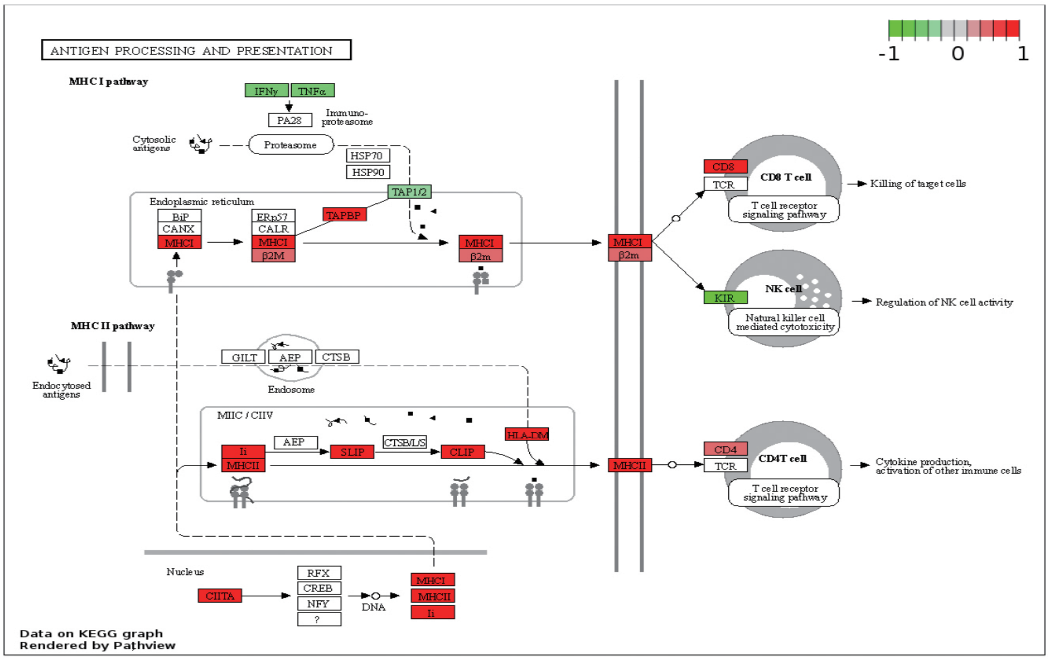

图 4　抗原处理和呈递信号通路

杨梅黄酮对皮肤光老化及 NF- κB 信号通路表达的抑制作用

（正文见第 1483 页）

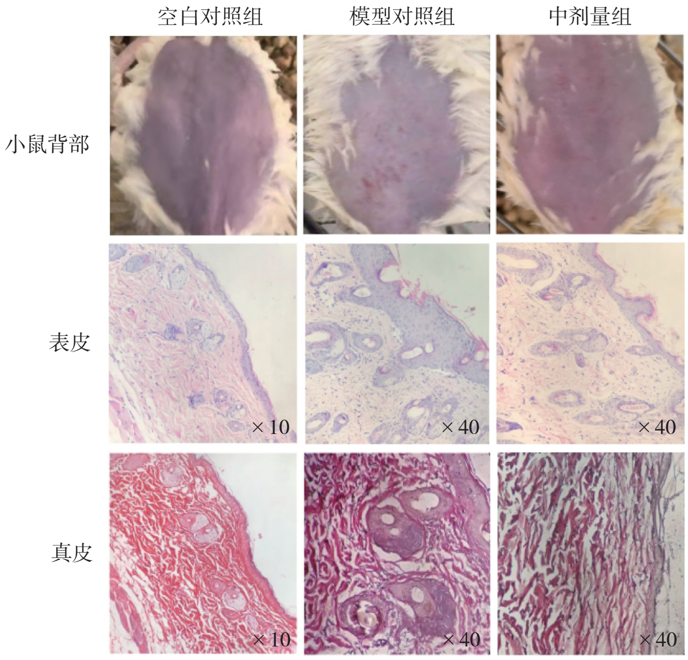

图 1　空白对照组、模型对照组及中剂量组小鼠经紫外线照射后皮肤的大体及组织形态学表现

(C)1994-2022 China Academic Journal Electronic Publishing House. All rights reserved.　http://www.cnki.net
